# Voice-Based Detection of Parkinson’s Disease Using Machine and Deep Learning Approaches: A Systematic Review

**DOI:** 10.3390/bioengineering12111279

**Published:** 2025-11-20

**Authors:** Hadi Sedigh Malekroodi, Byeong-il Lee, Myunggi Yi

**Affiliations:** 1 Industry 4.0 Convergence Bionics Engineering, Pukyong National University, Busan 48513, Republic of Korea; hadi_sedigh@pukyong.ac.kr; 2 Digital Healthcare Research Center, College of Information Technology and Convergence, Pukyong National University, Busan 48513, Republic of Korea; 3 Major of Human Bioconvergence, Division of Smart Healthcare, Pukyong National University, Busan 48513, Republic of Korea; 4 Major of Biomedical Engineering, Division of Smart Healthcare, Pukyong National University, Busan 48513, Republic of Korea

**Keywords:** parkinson’s disease, speech analysis, signal processing, machine learning, deep learning, early diagnosis

## Abstract

Parkinson’s disease (PD) is a progressive neurodegenerative disorder characterized by motor and non-motor symptoms, among which vocal impairment is one of the earliest and most prevalent. In recent years, voice analysis supported by machine learning (ML) and deep learning (DL) has emerged as a promising non-invasive method for early PD detection. We conducted a systematic review searching PubMed, Scopus, IEEE Xplore, and Web of Science databases for studies published between 2020 and September 2025. A total of 69 studies met the inclusion criteria and were analyzed in terms of dataset characteristics, speech tasks, feature extraction techniques, model architectures, validation strategies, and performance outcomes. Classical ML models such as Support Vector Machines (SVMs) and Random Forests (RFs) achieved high accuracy on small, homogeneous datasets, while DL architectures, particularly Convolutional Neural Networks (CNNs), Recurrent Neural Networks (RNNs), and Transformer-based foundation models, demonstrated greater robustness and scalability across languages and recording conditions. Despite these advances, persistent challenges such as dataset heterogeneity, class imbalance, and inconsistent validation practices continue to hinder reproducibility and clinical translation. Overall, the field is transitioning from handcrafted feature-based pipelines toward self-supervised, representation-learning frameworks that promise improved generalizability. Future progress will depend on the development of large, multilingual, and openly accessible datasets, standardized evaluation protocols, and interpretable AI frameworks to ensure clinically reliable and equitable voice-based PD diagnostics.

## 1. Introduction

Parkinson’s Disease (PD) is a prevalent and progressive neurodegenerative disorder, ranking as the second most common neurodegenerative condition after Alzheimer’s disease [1,2]. It arises from the gradual death or dysfunction of 60–80% of dopamine-producing neurons in the substantia nigra, an organic chemical vital for controlling movement. PD is typically characterized by motor symptoms such as tremors (shaking), rigidity (inflexibility), bradykinesia (slow movement), and impaired balance [3,4]. Non-motor symptoms, including cognitive impairment, depression, and sleep disturbances, are also common [5]. With a rising global aging population, the prevalence of PD, which is estimated at 1% among individuals over 60 years old, is expected to increase markedly [6,7].

Traditional diagnosis of PD is a complex process relying on clinical assessments, neurological examinations, and the observation of symptoms, often resulting in diagnostic delay. Clinical diagnosis is formalized using scales like the Movement Disorder Society-sponsored revision of the Unified Parkinson’s Disease Rating Scale (MDS-UPDRS) [8] and the Hoehn & Yahr (H&Y) scale [9]. Crucially, by the time motor dysfunctions manifest clinically, up to 50% of dopaminergic neurons may be irreversibly damaged [10,11]. In this context, recent advances in artificial intelligence (AI), cloud analytics, and the Internet of Things (IoT) are reshaping neurodegenerative diseases diagnosis by enabling earlier detection through real-time monitoring, large-scale data integration, and automated clinical decision support [12,13,14,15,16].

Vocal impairment is a prominent and early non-motor symptom of PD. Approximately 70% to 90% of PD patients experience voice disorders, which may appear years before motor symptoms, sometimes up to five years prior [17,18]. Voice analysis can provide a non-invasive, cost-effective, and easily accessible method for early PD detection and remote health monitoring [19,20]. In recent years, the increasing accessibility of digital recording devices and the rapid advancement of computational intelligence have opened new frontiers in automated voice-based PD detection. Beyond PD, speech-based deep learning frameworks have demonstrated strong potential for identifying other neurological and psychiatric conditions, such as depression. Studies have shown that vocal tone, rhythm, and prosodic changes can serve as reliable biomarkers of emotional state and mental health [21,22,23,24]. Machine learning (ML) and deep learning (DL) methods are now capable of analyzing subtle acoustic deviations beyond the perceptual limits of human clinicians.

Early research in this domain primarily relied on traditional ML techniques, such as Support Vector Machines (SVMs), Random Forests (RFs), and k-Nearest Neighbors (KNNs), trained on handcrafted acoustic features like Mel-Frequency Cepstral Coefficients (MFCCs), jitter, shimmer, and Harmonics-to-Noise Ratio (HNR) [25,26,27,28]. While these approaches demonstrated promising classification performance, they were limited by their dependency on manual feature design and their sensitivity to recording conditions [29,30].

More recently, DL architectures, including Convolutional Neural Networks (CNNs), Recurrent Neural Networks (RNNs), and Transformer-based models, have been introduced to capture temporal and spectral variations directly from raw or minimally processed audio signals [31,32,33,34]. These end-to-end frameworks can often be enhanced through self-supervised pretraining, enabling the automatic extraction of robust representations and holding potential for improved generalization across speakers, languages, and recording setups. Overall, the shift towards AI aims to provide objective, automated diagnostic tools to enhance diagnostic accuracy, reduce costs, and improve patient quality of life through timely intervention.

Despite these advancements, several challenges persist. The heterogeneity of datasets, small sample sizes, differences in recording protocols, and lack of standardized evaluation practices hinder the comparability and reproducibility of reported results. Moreover, few studies have assessed different aspects of methodological choices, such as feature extraction, model type, or validation strategy [30,35,36].

Given the growing body of literature and the diversity of experimental paradigms, this systematic review tries to address this need by providing a comprehensive examination of studies employing ML and DL techniques for PD detection through voice and speech analysis, covering research published between January 2020 and September 2025. Earlier reviews, such as those by Ngo et al. [37] and Rabie et.al [38], examined ML/DL approaches, including speech, gait, physiological signals, and neuroimaging. For example, Altham et al. [39] assessed the feasibility and impact of ML approaches, highlighting the versatility of these methods across various modalities like imaging, EEG, and speech, for detecting and diagnosing cognitive impairment in PD. In contrast, this review focuses solely on voice-based methods. The ML techniques included classical supervised algorithms such as SVM, RF, k-NN, and Decision Trees, among others. The DL approaches encompassed CNNs, RNNs, LSTM/GRU, Generative Adversarial Networks (GANs), and Transformer-based foundation models. It aims to summarize recent ML and DL approaches applied to PD detection using speech data, identify the most commonly used datasets, feature extraction methods, and model architectures, evaluate their diagnostic accuracy, robustness, and generalizability, and examine how current studies have addressed key challenges related to dataset bias and model transparency.

## 2. Methods

We conducted a systematic review of studies using ML or DL to detect or classify PD from speech or voice. The review followed the PRISMA 2020 guidelines [40]; however, no formal protocol was pre-registered. Details of the sources, search process, inclusion criteria, study selection, and data extraction are provided below.

### 2.1. Search Strategy and Databases Used

To investigate voice-based detection of PD using ML and DL, we searched four major databases, PubMed, Web of Science, IEEE Xplore, and Scopus, for studies published between 2020 and 2025, with the final search completed on 7 September 2025. A structured set of keywords and Boolean operators was employed to query the selected databases. The search strategy included terms related to PD (e.g., “Parkinson* disease”, “Parkinsonism”, “PD”), vocal characteristics (e.g., “speech”, “voice”, “vocal*”, “voice signal*”, “acoustic*”), and computational methods (e.g., “machine learning”, “deep learning”, “artificial intelligence”, “AI”, “neural network*”). These were combined with diagnostic-related terms such as “detect*”, “diagnos*”, “classif*”, and “screen*”. Filters were applied to limit results to full-text, English-language journal articles. Where possible, database-specific filters were used to include only English-language, full-text journal articles. Since metadata differ between databases, all records were later reviewed manually, and any eligible studies missing an “Article” tag were added. The specific search strings tailored for each database are provided in Supplementary Table S1. All retrieved records were exported in RIS format and managed using Zotero (v7.0) for organization and duplicate removal prior to screening.

### 2.2. Inclusion and Exclusion Criteria

To maintain alignment with the review objectives and provide a thorough overview of the research on PD, specific inclusion and exclusion criteria were established. Included studies focused on the classification, diagnosis, detection, or identification of PD and applied ML or DL techniques for data processing and modeling. They also involved datasets related to voice, speech, or language processing. Additionally, only peer-reviewed, full-text articles published in English were considered. Although the review explores cross-lingual robustness in PD voice analysis, the literature search was limited to English-language publications to ensure accurate methodological interpretation and consistent data extraction. Nevertheless, bilingual or multilingual datasets (e.g., non-English speech corpora accompanied by English documentation) were considered eligible and included.

At the full-text screening stage, studies were excluded for several specific reasons. Articles that did not include voice- or speech-based data were removed when the reported biomarkers were derived from non-acoustic modalities such as gait, EEG, handwriting, or imaging. Studies that lacked implementation of ML or DL techniques for PD detection were excluded when voice features were analyzed using only conventional statistical or signal-processing methods without predictive modeling. Works relying on non-original datasets without adequate methodological transparency were also excluded. In addition, studies focusing primarily on other neurological or neurodegenerative disorders without a distinct PD subgroup analysis were omitted. Finally, full-text papers that did not report essential methodological or dataset details, such as participant numbers, feature sets, or model configurations, were excluded.

Conference and workshop proceedings, reviews, meta-analyses, books, book chapters, and editorials were also excluded, as these sources typically lack full methodological transparency and peer-review rigor consistent with journal publications. When an item appeared as both an early-access article and a later indexed version, the record with the complete citation metadata was kept.

### 2.3. Screening and Selection Process

The study selection was carried out based on clearly defined inclusion and exclusion criteria. The final literature search was completed on 7 September 2025 across all four databases (PubMed, Scopus, Web of Science, and IEEE Xplore). In the first stage, titles and abstracts were screened, where exclusion criteria were applied with a relatively inclusive approach. Studies were excluded at this point if the title or abstract clearly indicated that the work was not related to PD, was unrelated to voice or speech, lacked ML or DL methods, or was not written in English. For records where relevance was unclear, the full texts were reviewed to determine eligibility. Full-text screening was then conducted to assess methodological and reporting completeness. Studies were included only if they analyzed human voice or speech data related to PD, employed ML or DL methods for detection, classification, or analysis, and provided sufficient methodological information to ensure transparency and reproducibility. Studies were excluded if they lacked full-text access, did not focus on PD or voice-based data, failed to employ ML/DL approaches, or provided insufficient methodological and dataset details. Screening was performed collaboratively by all authors, with any disagreements or uncertainties discussed and resolved collectively to ensure consistency and minimize bias.

### 2.4. Data Extraction

For each included study, relevant data were systematically extracted using a structured template. This process involved collecting detailed information about the voice and language resources employed, including the dataset name, data collection procedures, and participant demographics. Technical details such as the recording device, audio format with sampling rate, and average duration per sample were also recorded. Additionally, the type of voice task performed (such as sustained vowels, reading sentences), the noise environment during recording, diagnostic tools used to confirm PD, and the language of the recorded speech were documented. Lastly, dataset availability (public or private) and the number of studies utilizing each dataset in the review were noted. In parallel, methodological details were extracted to analyze how each study approached PD detection. This included the authors, year of publication, task type, dataset, voice features, applied ML/DL method, evaluation approach (e.g., cross-validation or train-test split), and best reported performance metrics such as accuracy, sensitivity, specificity, F1-score, and AUC. Performance values were recorded as reported in each study, typically representing average accuracy or AUC across folds or the final result for single-split validation. Confidence intervals or variance measures were rarely provided and thus not summarized. Task-specific sensitivity analyses (e.g., sustained vowels vs. reading) were not conducted due to inconsistent reporting.

Additional observations, such as feature selection methods, external validation, and limitations reported by the authors, were included where applicable.

### 2.5. Risk of Bias Assessment

This review focuses on diagnostic approaches using voice and machine learning, not clinical interventions, so conventional risk-of-bias tools are not applicable. Nevertheless, methodological biases are common in ML/DL studies, particularly from imbalanced or non-representative datasets, small sample sizes, overfitting due to improper data splitting, and overreliance on accuracy in skewed settings. Rather than applying a formal risk of bias instrument, we address these concerns in the Discussion section by evaluating key indicators such as dataset balance, validation rigor, and appropriateness of reported metrics.

## 3. Results

**Figure 1:**
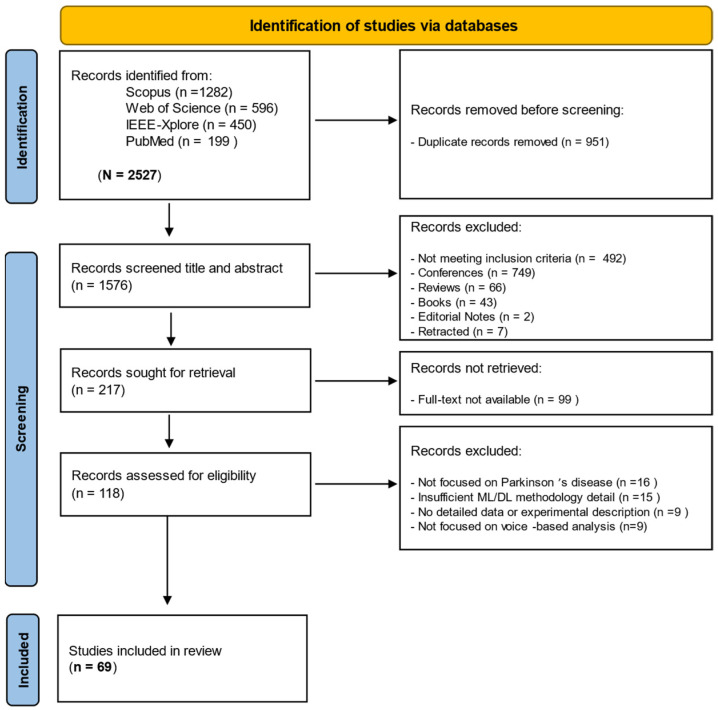
Description of the study selection process.

A literature search across four major databases, PubMed, Web of Science, IEEE Xplore, and Scopus, identified 2527 records. After removing 951 duplicates, 1576 studies remained for title and abstract screening. Of these, 1358 were excluded due to not meeting the eligibility criteria or being classified as review articles, systematic reviews, conference proceedings, books, or editorials. Full-text retrieval was attempted for 217 records, of which 99 could not be accessed despite institutional and interlibrary search efforts, leaving 118 for detailed eligibility assessment. For full-text review, the primary reasons for exclusion are summarized in Figure 1. Specifically, among the 49 studies excluded at the full-text stage, 9 lacked voice-based data, 15 did not apply ML/DL methods, 9 had insufficient methodological or dataset details, and 16 did not focus on PD. Ultimately, 69 studies met all inclusion criteria and were included in the final synthesis. The complete selection process is illustrated in Figure 1, following the PRISMA 2020 guidelines [40].

**Figure 2:**
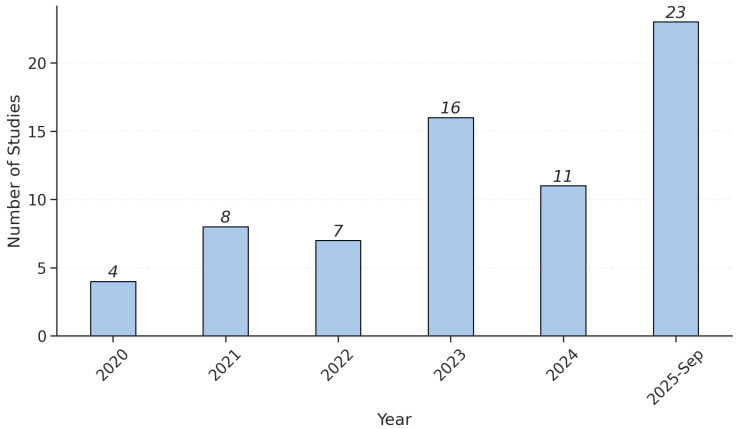
Annual distribution of selected studies from 2020 to 2025.

Figure 2 illustrates the annual distribution of studies included in this review from 2020 to 2025. A steady increase in the number of eligible publications is evident over time, rising from just 4 studies in 2020 to a peak of 23 in 2025. The most notable growth occurred between 2023 and 2025. This trend suggests increasing research activity in the field during the later years of the review period.

The Supplementary Material comprises two comprehensive tables designed to synthesize and organize critical data extracted from the 69 studies included in this review. The first table (Table S1) provides an overview of the datasets used across the selected studies, including their source, size, demographic composition, and whether they were publicly available or not. The second table (Table S2) outlines the methodological frameworks employed, detailing the ML or DL models applied, feature extraction techniques, evaluation metrics, and performance outcomes reported in each study. These tables serve as a structured reference for readers seeking to compare approaches, identify trends, or evaluate methodological consistency across the literature. Most studies employed binary classification frameworks to distinguish PD patients from healthy controls (HCs), with accuracy or AUC as the primary performance metric.

### 3.1. Dataset Characteristics

Over the last five years, a diverse collection of publicly and privately curated speech datasets has supported the development of ML and DL models for PD detection. Across all studies in this review, a total of 29 distinct datasets were identified, many of which were repeatedly used across publications. These datasets differ in size, participant demographics, and data collection methodologies, with sample sizes ranging from under 50 to several hundred participants (including both PD patients and HCs), though often exhibiting class imbalance. Most participants are aged 50–75, and the gender distribution is often uneven. Data was collected in clinics, labs, or via mobile/home devices. Supplementary Table S2 provides a detailed summary of these datasets. In the following section, we introduce the most commonly used dataset in the literature, providing a closer examination of its structure and role in PD-related voice research.

**Figure 3:**
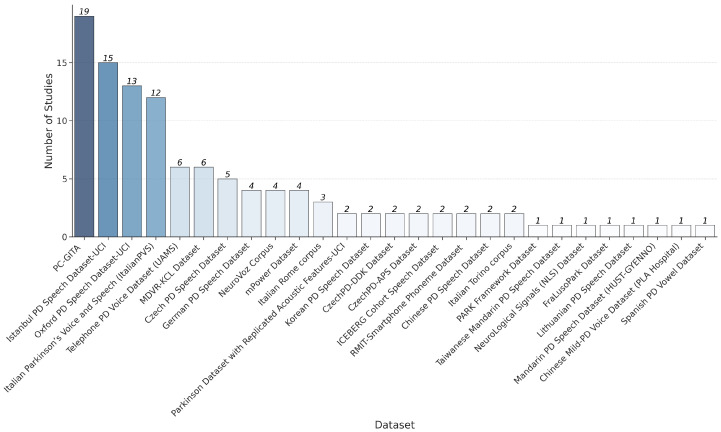
Frequency of PD voice datasets used in reviewed studies. PC-GITA [41], Istanbul PD Speech (UCI) [42], Oxford PD Speech (UCI) [43], and ItalianPVS [44] were the most frequently used datasets.

As shown in Figure 3, some datasets appear repeatedly across the literature reviewed: PC-GITA [41] (Spanish), the Istanbul PD Speech Dataset [42] (UCI, Turkish), the Oxford PD Speech Dataset [43] (UCI, English), the Italian Parkinson’s Voice and Speech [44,45] (Italian), and both the Telephone PD Voice Dataset (UAMS) [46] and Mobile Device Voice Recordings at King’s College London (MDVR-KCL) [47] (English), which occur with equal frequency.

Early benchmark datasets from the UCI Machine Learning Repository continue to serve as methodological cornerstones. The Oxford PD Speech Dataset [43] (“parkinsons”; 31 participants, 195 sustained /a/ phonations) remains widely used in baseline and proof-of-concept studies due to its clean acoustic design and public availability. Its companion, the Parkinson’s Telemonitoring Dataset [48] (42 participants, 5875 home-recorded phonations), uniquely links voice features with longitudinal motor and total UPDRS scores, establishing it as the go-to resource for symptom severity regression rather than binary classification. The dataset appeared in 14 of the studies reviewed here. The main limitations are limited demographic diversity, small sample sizes, and a lack of linguistic or environmental variability. The Istanbul PD Speech Dataset [42] (252 participants: 188 PD, 64 HC) significantly expands scale and documentation. Recorded in a quiet clinical setting using a standardized sustained-vowel protocol, it includes detailed metadata on age, sex, and Hoehn & Yahr staging. Its scale, accessibility, and acoustic consistency make it a frequent choice for comparative ML and feature-engineering studies, appearing in 15 reviewed works. However, its class imbalance (≈3:1 PD:HC ratio) can constrain its utility for generalization-focused research.

PC-GITA [41] (Spanish; 100 participants, ~6300 recordings) is the most frequently cited dataset in this review (19 studies). It offers a rich variety of speech tasks, including sustained vowels, diadochokinetic (DDK) syllables, read passages, and spontaneous monologues, recorded under controlled conditions with professional equipment. Its demographic balance (50% female in both PD and HC groups), clinical validation, and multi-task design support both traditional ML pipelines (e.g., prosodic or cepstral features) and end-to-end DL. While its scale and internal consistency make it ideal for cross-task and cross-model comparisons, its exclusive use of Colombian Spanish limits cross-lingual transferability.

To address linguistic generalization, several language-specific datasets have become influential. The Italian Parkinson’s Voice and Speech (ItalianPVS) dataset [44] (50 participants, sustained vowels and phonetically balanced sentences) provides carefully annotated recordings from Italian speakers; its open availability has made it a frequent inclusion in cross-lingual DL comparisons. It appeared in 12 studies and provides a valuable resource for European languages. Complementary private collections, such as the Italian Torino Dataset [49,50] (home- and clinic-recorded /a/ phonations), enable investigations into real-world recording variability and medication-state effects. Similarly, the German PD Speech Dataset (GermanPD) [51] (Bochum; 88 PD/88 HC) and Czech PD Speech (CzechPD) [52] (Prague; 50 PD/50 HC) dataset contributes European-language diversity, offering structured recordings (sustained vowels, DDK syllables, monologues) with standardized microphones for inter-task robustness studies. Newer Spanish resources further enrich the landscape. NeuroVoz [53] (≈112 participants, Castilian Spanish) offers clinically annotated, ON-medication recordings across multiple speech tasks, with UPDRS metadata, making it valuable for early biomarker discovery and interpretable modeling. The FraLusoPark dataset [54] (140 participants across France and Portugal) incorporates bilingual speech to assess dopaminergic medication effects, providing material for ON/OFF state classification using deep architectures. Its structured protocol, including storytelling, reading, and prosody tasks, has made it useful for comparison. The ICEBERG dataset [11] (247 participants, French) offers rich multimodal speech tasks, including free monologue, sentence repetition, and DDK syllables, recorded both in-clinic and via telephone, supporting early PD detection research in two studies.

Reflecting a broader shift toward real-world applicability, recent efforts emphasize mobile-collected speech. The Telephone PD Voice Dataset (UAMS) [46] (81 participants) captures 3 s /a/ phonations via standard telephone lines (8 kHz, narrowband). Despite limited spectral fidelity, CNNs trained on mel-spectrograms achieve AUC ≈ 0.95–0.97, underscoring their viability for remote, low-resource screening, particularly in rural or underserved populations [55]. The MDVR-KCL Dataset [47] (37 participants) extends this paradigm using smartphone-recorded spontaneous dialogue and read speech in naturalistic settings, enabling studies of device heterogeneity and ambient noise robustness. In this review, both datasets were used in six different studies. At the frontier of scale and diversity is the PARK Framework Dataset [56] (1306 participants), which uses the English pangram “*The quick brown fox jumps over lazy dog*” recorded across home, clinical, and care-facility environments via consumer devices (iPhones, laptops). Its multi-environment design supports generalizability studies across acoustic conditions. Even more expansive is the mPower Dataset [57] (>5800 participants, >65,000 recordings), which aggregates smartphone-captured /a/ phonations in completely uncontrolled settings. Its massive scale, longitudinal structure, and real-world noise profile make it indispensable for transfer learning, domain adaptation, and developing noise-resilient digital biomarkers, effectively bridging the gap between laboratory-controlled studies and population-scale monitoring.

Recent efforts have extended PD speech research into East Asian languages, addressing a critical gap in global representation. The Korean PD Speech Dataset [58,59] (291 participants) includes sustained vowels, syllable repetition, and reading tasks recorded via smartphone in clinical settings, and has been used in two studies to validate cross-task and cross-cultural acoustic biomarkers. Similarly, multiple Mandarin Chinese datasets, including the Mandarin PD Speech Dataset [25] (100 participants), the Chinese Mild-PD Voice Dataset [60] (278 participants, with a focus on early-stage, OFF-medication recordings), and the smaller GYENNO Sentence Dataset [17,33] (45 participants), enable investigation of tonal and articulatory features unique to Chinese. These datasets, used in 1–2 studies each, demonstrate that vowel/o/ and tongue twisters carry strong discriminative power in tonal languages. Despite their clinical value, none are publicly available, which limits reproducibility.

The usage frequency of the datasets in this review, as shown in the bar chart in Figure 3, reveals a clear hierarchy. One dataset stands out as the most frequently used, with several others following closely behind. This pattern underscores their established role as foundational benchmarks, recognized for their comprehensive documentation, open accessibility, and standardized protocols. Notably, smartphone-collected datasets like MDVR-KCL [47] and Telephone PD Voice Dataset (UAMS) [46] appear in six studies each, while emerging resources such as the Korean PD Speech Dataset [58,59] (two studies) and Taiwanese Mandarin PD Speech Dataset [58] (one study) signal growing interest in East Asian languages. In summary, the field is evolving from small, clean, controlled datasets toward large, multilingual, mobile, noisy datasets, better reflecting the heterogeneity and constraints of real-world clinical deployment.

While we focus on the most widely used datasets, further comprehensive details on other datasets [30,61,62,63,64,65,66,67], including participant counts, tasks, recording conditions, and usage frequency, are provided in the Supplementary Table S2.

### 3.2. Voice Tasks and Recording Protocols

Across the reviewed studies, voice data were collected using a range of phonatory and speech-based tasks designed to capture the vocal impairments characteristic of PD. The most common protocol involved sustained phonation of a single vowel sound, typically /a/, held for several seconds at a comfortable pitch and loudness. This task isolates fundamental voice parameters such as jitter, shimmer, and harmonic-to-noise ratio, which are sensitive to the hypophonia and dysarthria associated with PD. For instance, the Istanbul PD Speech Dataset [42] and Oxford PD Speech Dataset [43] both rely exclusively on sustained /a/ phonations recorded in controlled acoustic environments, making them widely used benchmarks for ML validation. Beyond sustained vowels, many datasets incorporated more complex speech tasks to assess articulatory precision, prosody, and fluency. DDK syllable repetition tasks, such as /pa/, /ta/, /ka/, or /pa-ta-ka/, were widely used to evaluate motor speech speed and coordination [41,53,59]. Reading tasks, including phonetically balanced sentences, standardized passages (e.g., “*The North Wind and the Sun*” in the MDVR-KCL dataset [47]), or culturally specific texts, enabled analysis of connected speech under controlled conditions. Some datasets further included spontaneous monologues, picture description tasks (e.g., the Cookie Theft picture in NeuroLogical Signals (NLS) dataset [30]), or free conversation to capture naturalistic speech patterns, prosodic variation, and discourse-level features.

**Figure 4:**
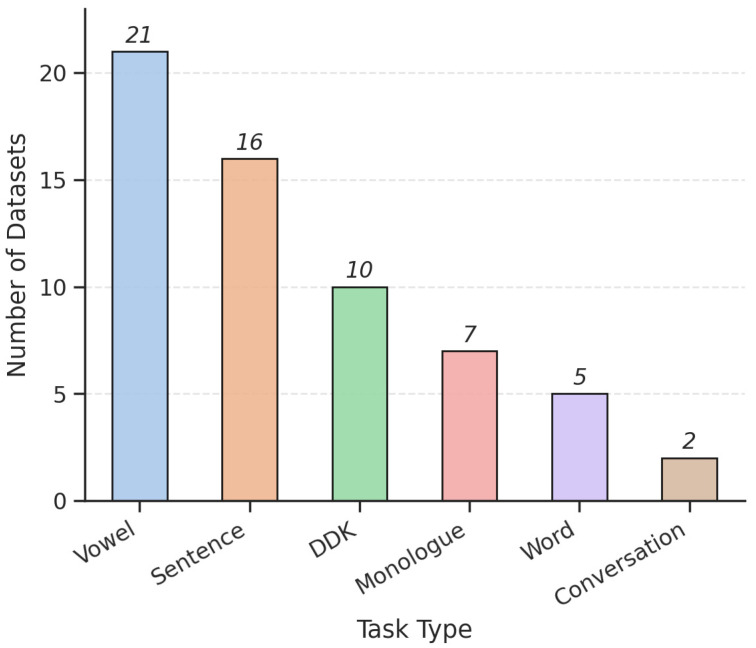
Distribution of datasets by speech task type used in PD voice research, showing a predominance of vowel phonation tasks, followed by sentence reading and DDK repetition.

The bar chart in Figure 4 illustrates the distribution of datasets according to the type of speech task used in voice-based PD research. Vowel phonation tasks are the most prevalent, appearing in 21 datasets, followed by sentence reading in 16 datasets and diadochokinetic (DDK) repetition in 10. This distribution highlights a strong preference for controlled, standardized speech protocols in current research. Vowel phonation is favored for its simplicity and sensitivity to core vocal impairments like jitter and reduced loudness, making it ideal for consistent acoustic analysis. Sentence reading allows assessment of prosody and fluency in connected speech, offering richer linguistic data. The dominance of these structured tasks highlights a focus on reproducibility, but also indicates limited use of spontaneous speech, which could better capture real-world communication challenges in PD.

Task duration varied from brief 2–5 s vowel phonations to multi-minute monologues or dialogues, reflecting a trade-off between clinical practicality and real-world richness. Examples include the PC-GITA [41], which features a comprehensive collection of tasks in Spanish (vowels, DDK, sentences, reading, and monologues), and the FraLusoPark Dataset [54], which includes storytelling, prosody-specific sentences, and 3-min free conversations in French and European Portuguese. The mPower [57] and PARK Framework [56] datasets exemplify real-world approaches, using smartphone-recorded sustained /a/ phonations or pangram utterances (“*The quick brown fox jumps over the lazy dog*”) collected in uncontrolled environments to support scalable, remote monitoring.

**Figure 5:**
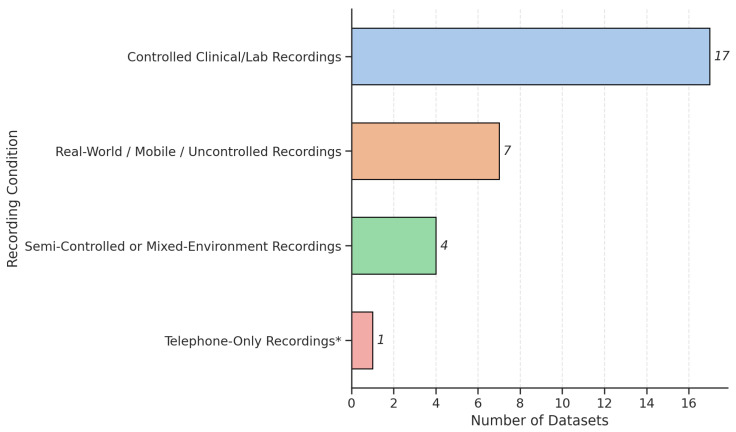
Distribution of PD Voice Datasets by Recording Condition. * The Telephone PD Voice Dataset (UAMS) is the only dataset exclusively telephone-based; ICEBERG includes it as a modality alongside high-quality recordings.

Recording protocols also differed significantly across studies, influenced by setting, equipment, and research goals. Controlled environments, such as soundproof booths or quiet clinical rooms, were typical in lab-based datasets (e.g., PC-GITA [41], Oxford PD Speech Dataset [43]), ensuring high signal quality for acoustic analysis. In contrast, real-world or telemonitoring datasets (e.g., mPower [57], PARK Framework [56] datasets) embraced uncontrolled environments, using smartphones or telephone systems to enhance accessibility and scalability, albeit at the cost of increased acoustic variability. The bar chart, as shown in Figure 5, shows the distribution of datasets across four recording conditions. Most datasets (17) were collected in controlled clinical or lab settings to ensure high acoustic quality. Real-world or mobile recordings (8) reflect growing interest in remote monitoring, despite background noise challenges. Semi-controlled environments (4) offer a balance between control and realism, while one dataset (Telephone PD Voice Dataset (UAMS) [46]) represents only telephone-based recordings. This highlights a trade-off between data quality and real-world application.

Microphone types ranged from professional head-mounted condensers placed 5–10 cm from the mouth to built-in smartphone mics, with sampling rates from 8 kHz to 96 kHz. These setups reflect a spectrum from controlled clinical protocols to real-world data collection for broader applicability. Some studies used studio-quality equipment in treated environments (16–44.1 kHz), while others, like the PARK Framework, relied on consumer devices in uncontrolled settings [56].

**Figure 6:**
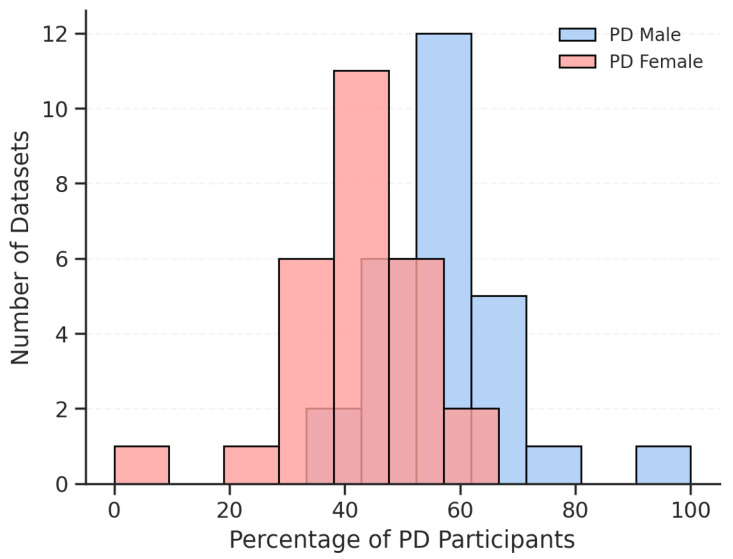
Distribution of male and female PD patients across datasets, showing a higher prevalence of males in most studies.

Demographic characteristics also varied. Age ranges typically spanned from approximately 60 to 70 years, aligning with the peak PD incidence window. Gender distribution was moderately balanced in some datasets (e.g., PC-GITA: 25M/25F per group [41]) but skewed in others (e.g., Oxford PD Dataset: 16M/7F PD group [43]). Such imbalances may bias model performance if gender-related vocal characteristics are not properly controlled [68]. Figure 6 shows the gender distribution of PD patients across datasets. Most datasets have a higher percentage of male participants, peaking between 50–60%, while female representation is more varied, with a peak between 40–50%. This male predominance likely reflects the higher incidence of PD observed in men compared to women [69,70].

**Figure 7:**
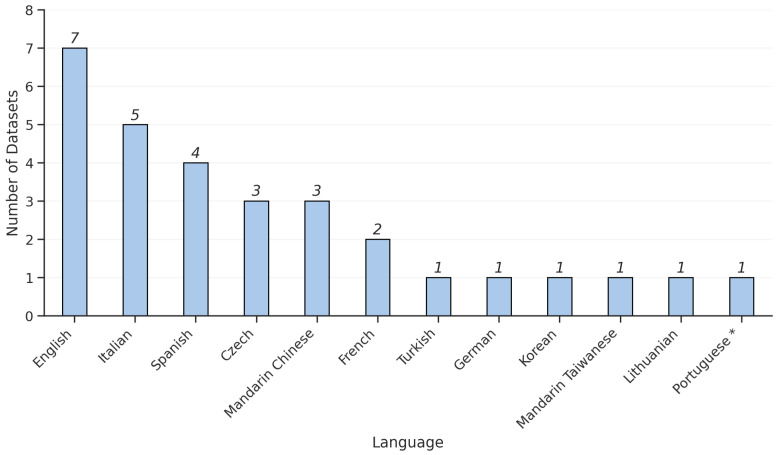
Number of PD voice datasets by language. English-based datasets dominate (7 studies), followed by Italian (5), Spanish (4), and Chinese, Czech (3). Other languages are represented by only one or two datasets. * The FraLusoPark Dataset is a bilingual resource that includes both French and Portuguese.

In addition, as depicted in Figure 7, the datasets collectively represent three major languages, English, Spanish, and Italian, reflecting the international scope of PD voice research. However, language-specific phonetic structures introduce variability in the acoustic correlates of PD symptoms, which may complicate cross-dataset generalization. For instance, the PC-GITA dataset (Spanish) and the Oxford PD dataset (English) differ markedly in vowel duration and articulation norms, potentially influencing feature distributions.

### 3.3. Feature Extraction and Input Data

**Figure 8:**
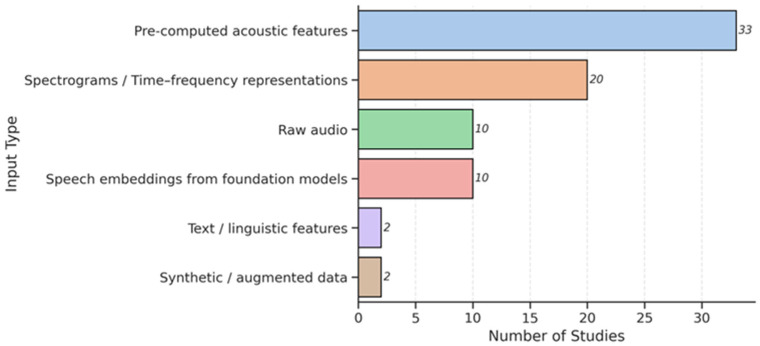
Distribution of input types used in reviewed studies for PD detection, with pre-computed acoustic features being the most commonly used. Some studies fall into multiple categories.

Across the reviewed studies, two primary paradigms emerged for representing speech signals: handcrafted (feature-based) approaches and learned (raw-audio-based) representations. As illustrated in Figure 8, approximately two-thirds of the analyzed studies employed handcrafted acoustic descriptors derived from sustained vowel phonations or controlled reading tasks. A growing subset, particularly those published from 2023 onward, leveraged DL models that learn representations directly from raw waveforms, spectrograms, or self-supervised embeddings.

Handcrafted features are typically extracted using established toolkits such as Praat [71], openSMILE [72], or DisVoice [73]. These features draw from well-documented acoustic biomarkers associated with PD-related dysphonia, including perturbation measures (jitter, shimmer), harmonics-to-noise ratios (HNR, NHR), nonlinear dynamics (RPDE, DFA, PPE), and spectral descriptors (MFCCs, formants, RASTA-PLP). These features are commonly paired with classical ML classifiers, such as SVM, RF, or XGBoost, and often enhanced through techniques like Recursive Feature Elimination (RFE), Principal Component Analysis (PCA), or class balancing (e.g., SMOTE).

In contrast, learned representations bypass manual feature engineering by feeding raw audio or time–frequency representations directly into DL architectures. This includes convolutional neural networks (CNNs; e.g., ResNet, VGG, Inception), recurrent models (LSTM, GRU), Transformers, and self-supervised foundation models such as Wav2Vec 2.0 [74], HuBERT [75], Whisper [76], and WavLM [77]. Input modalities under this paradigm vary widely; some studies use raw waveform segments, others transform audio into spectrograms (Mel, STFT, Superlet) or leverage pretrained embeddings generated via fine-tuning or mean-pooling over intermediate layers. Notably, the adoption of speech embeddings from foundation models has grown significantly in recent years, reflecting a broader trend toward leveraging large-scale pretraining for downstream clinical tasks.

A few works explored generative approaches, primarily to address data scarcity and class imbalance [78,79]. For instance, Xu et al. [78] introduced an S-DCGAN (Spectrogram Deep Convolutional Generative Adversarial Network) to synthesize high-fidelity spectrograms for data augmentation, while Rey-Paredes et al. [79] used a GAN called BigVSAN to generate realistic raw waveform segments. These approaches aim to improve model robustness by expanding the training distribution without collecting additional clinical data, though concerns remain about the fidelity and clinical validity of synthetic samples. Another emerging direction involves the integration of text or linguistic features, typically derived from automatic speech recognition (ASR) transcriptions [58,80]. Escobar-Grisales et al. [80] combined Wav2Vec 2.0 embeddings with Spanish BERT (BETO) embeddings from transcribed spontaneous speech, though the multimodal fusion underperformed compared to audio-only models. Similarly, Lim et al. [58] extracted linguistic descriptors, such as speech rate, pause percentage, and word error rate, from ASR (Automatic Speech Recognition) outputs (via Google Speech-to-Text) and fused them with acoustic features, finding that longer, linguistically rich tasks like reading full passages yielded stronger diagnostic signals than isolated vowels.

Figure 8 summarizes the input data types across the reviewed studies. “Pre-computed acoustic features” were most prevalent (33 studies), reflecting their established role in voice pathology research. Spectrograms or time-frequency representations followed (20 studies), aligning with the widespread use of CNNs. Speech embeddings from foundation models appeared in 10 studies, signaling rapid adoption. Raw audio was used in 10 studies, typically within end-to-end DL frameworks. Less common were text/linguistic features (2 studies), synthetic/augmented data (2 studies). Overall, the field is shifting from interpretable, task-specific features toward flexible, data-driven representations.

### 3.4. Machine-Learning and Deep-Learning Models

**Figure 9:**
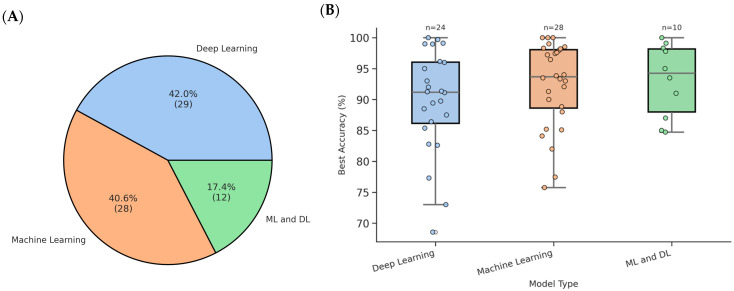
(**A**) Distribution of Model Types. The pie chart illustrates the distribution of model types used, with classical ML accounting for 40.6% (28 studies), DL for 42.0% (29 studies), and hybrid approaches combining both techniques representing 18.8% (12 models). (**B**) Distribution of the best accuracy (%) of model types used reported by reviewed studies. Boxplots show median and spread points represent individual studies.

In the studies selected, voice-based PD shows a balanced use of ML and DL methods, with some adopting hybrid models combining both. As illustrated in Figure 9A, among the 69 reviewed studies, 40.6% (n = 28) relied exclusively on classical ML models, 42.0% (n = 29) employed DL architectures, and 17.4% (n = 12) utilized hybrid systems that used DL for feature extraction and ML for classification or ensembles/hybrids of both. This distribution underscores a field in transition, while handcrafted features and interpretable classifiers remain foundational, DL, particularly foundation models and transformer-based architectures, is rapidly gaining ground, driven by advances in self-supervised representation learning [56,81,82,83]. In addition, the box plot in Figure 9B compares the best accuracy (%) achieved by these three types of models across studies reviewed. Each model type shows high median accuracy, with all medians above 90%. 

ML methods continue to dominate in settings where interpretability, computational efficiency, and performance on small datasets are important. The most frequently used classifiers include SVM, RF, KNN, Gradient Boosting Machines (XGBoost, LightGBM), and Decision Trees. SVM was the single most prevalent model, appearing in over half of all ML-based studies, such as Alalayah et al. [84], Qasim et al. [85], Amato et al. [49], and Karapinar Senturk [86], often paired with RBF kernels and enhanced through feature selection and class balancing techniques like SMOTE. These models were typically, as explained in the previous section, trained on handcrafted acoustic features extracted from sustained vowel phonations or controlled reading tasks [28,87]. When applied to benchmark feature-based datasets [42,43,61], these pipelines consistently achieved high accuracy, often exceeding 90–99% under subject-independent cross-validation [27,84,85,88,89]. However, this success is largely confined to small, controlled, and homogeneous datasets. Performance tends to degrade significantly when models are tested on external or in real-world conditions with variable recording environments [17,29,35,90,91].

**Figure 10:**
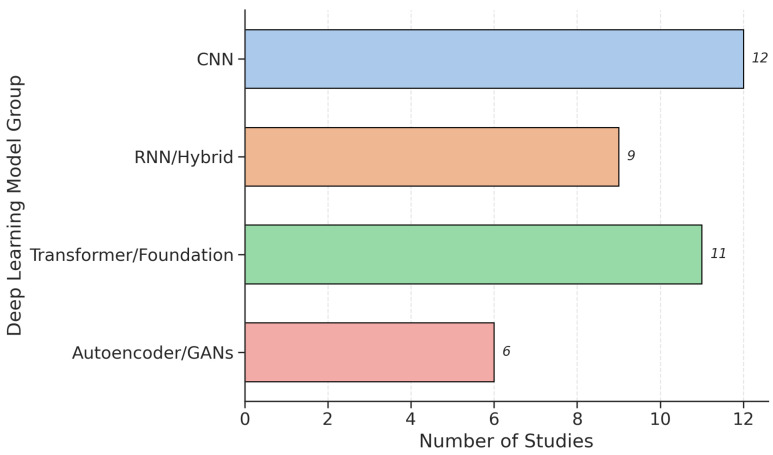
Distribution of studies employing different DL model groups. Convolutional Neural Networks (CNNs) were the most frequently used (12 studies), followed closely by Transformer/Foundation models (11 studies). Note that several studies incorporated multiple model architectures within the same work.

DL models have emerged as powerful alternatives, capable of automatically learning hierarchical representations directly from raw waveforms, spectrograms, or pre-trained embeddings. The dominant architectures include CNNs for spectral pattern recognition, Long Short-Term Memory (LSTM) networks for modeling temporal dynamics, and, more recently, transformer-based models for capturing long-range dependencies across speech segments. As shown in Figure 10, CNNs remain the most frequently used architecture in the current review, followed closely by transformer-based models, while RNN/Hybrid and Autoencoder/GAN approaches are less common.

Studies using CNNs and LSTMs, often applied to log-Mel spectrograms or time-frequency representations, have reported high performance, with several achieving >95% accuracy in subject-independent validation [34,55,88,92,93,94,95,96]. For instance, Bhatt et al. [96] introduced a High-Resolution Superlet Transform (SLT) to generate time–frequency inputs for VGG-16 and ResNet50V2, achieving 96% accuracy on the ItalianPVS dataset. Similarly, Iyer et al. [97] used an Inception V3 CNN on spectrogram images derived from telephone recordings, attaining an AUC of 0.97, demonstrating feasibility even with low-resolution audio. However, when evaluated across different languages and recording conditions, performance frequently declines. For example, Quan et al. [17] found that a federated CNN model achieved only 67–82% accuracy in cross-lingual scenarios (Spanish → Chinese, Italian → Chinese), underscoring the challenge of generalization. Moreover, DL models can be computationally intensive and prone to overfitting without careful regularization, data augmentation, or transfer learning strategies [78,79].

A notable evolution in recent years is the adoption of transformer-based architectures, which leverage self-attention mechanisms to model global context in speech sequences more effectively than CNNs or LSTMs. Adnan et al. [56] proposed a Transformer–Projection Fusion Model that integrates WavLM [77] and ImageBind [98] embeddings, achieving high accuracy on the large-scale PARK Framework Dataset (N = 1306). Similarly, Tougui et al. [81] fine-tuned an Audio Spectrogram Transformer (AST) on the mPower dataset [57], reaching 91.35% accuracy using smartphone-recorded vowels. Dao et al. [36] fine-tuned an ensemble of wav2vec 2.0, Whisper, and SeamlessM4T [99], attaining an AUC of 91.35% and strong correlation with clinical scores like MDS-UPDRS on the ICEBERG dataset [11]. In another study, Nijhawan et al. [100] proposed a transformer-based deep neural network, termed Vocal Tab Transformer, that classifies PD using complex dysphonia measures from voice recordings, outperforming gradient-boosted decision trees by about 1% AUC and improving precision and recall through XGBoost-based feature selection. These models offer several advantages, including the ability to capture both local and global phonatory variations related to dysarthria and tremor, adaptability to PD detection using limited labeled data through large-scale pretraining, and seamless integration of diverse acoustic, linguistic, and clinical features within a unified latent representation.

A small fraction of studies specifically used hybrid frameworks that aim to combine the strengths of both paradigms. These models typically use deep networks for feature extraction or representation learning and classical ML classifiers for decision-making, thereby balancing discriminative power with interpretability. Notable examples include Ali et al. [101], who developed an L1-regularized SVM → Deep Neural Network (L1SVM-DNN) cascade, achieving 100% accuracy on the Oxford PD dataset and 96.42% on Istanbul PD. The SVM first pruned redundant features before deep classification, enhancing both performance and stability. Celik et al. [102], who proposed a SkipConNet + RF hybrid, where a CNN extracted multidimensional feature vectors that were then classified by RF, yielding 99.11% accuracy on the Oxford PD dataset and 98.30% on the Istanbul PD. Other studies integrate clinically informed features with self-supervised embeddings via stacked ensembles that fuse acoustic, prosodic, and linguistic descriptors [30,59,83,103,104,105,106,107]. These hybrid approaches suggest a promising middle ground, leveraging DL for feature discovery while retaining classical ML for transparent, stable decision-making. They are particularly valuable in clinical settings where model explainability and reproducibility are critical for adoption.

In summary, while traditional ML remains highly effective for small, well-controlled datasets and offers superior interpretability, DL, especially foundation models and hybrid pipelines, offers a path toward scalable, multilingual, and real-world applicable systems. The near-parity in usage between ML and DL reflects a field at an inflection point, where researchers are increasingly moving beyond manual feature engineering toward self-supervised, representation-learning paradigms that can generalize across diverse populations and recording conditions.

### 3.5. Model Validations

**Figure 11:**
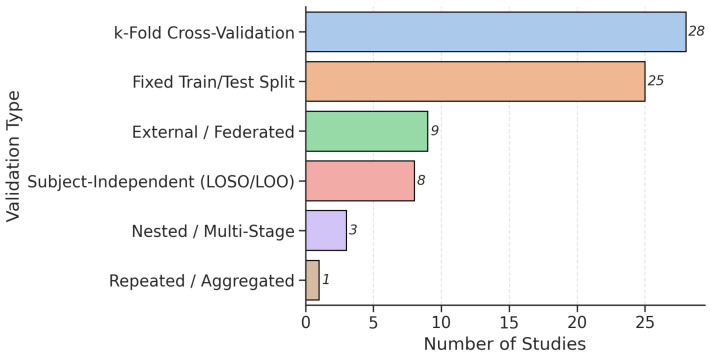
The frequency of different validation strategies across studies. k-Fold Cross-Validation is the most common approach (28 studies), followed by Fixed Train/Test Split (25). Less frequently used methods include External/Federated (9), Subject-Independent (LOSO/LOO) (8), Nested/Multi-Stage (3), and Repeated/Aggregated (1). Some studies used several approaches.

The validation strategies employed across the reviewed studies exhibit substantial heterogeneity, reflecting differences in dataset scale, model complexity, and research objectives. As illustrated in Figure 11, k-fold cross-validation was the most prevalent approach, adopted in 28 studies, followed by fixed train/test splits (25 studies). A small fraction employed subject-independent methods such as Leave-One-Subject-Out (LOSO) or Leave-One-Out Cross-Validation (LOOCV) (eight studies) and external/federated validation (nine studies), nested/multi-stage validation (three studies), or specifically repeated/aggregated evaluation (one study). This distribution underscores a field still dominated by internal validation paradigms but also highlights a critical gap in external generalizability assessment.

Internal validation remains the norm, with k-fold CV being the preferred method for its balance between statistical robustness and computational feasibility. Studies like Chintalapudi et al. [92] and Bhatt et al. [96] used a 10-fold CV to evaluate LSTM and CNN architectures on the Oxford dataset, achieving high accuracy (98.97% and 96%, respectively). Similarly, Hireš et al. [32,90] applied a 10-fold CV to compare XGBoost and Xception CNN across four datasets, reporting within-dataset accuracies above 90%.

Fixed train/test splits (e.g., 70/30, 80/20) were commonly used when datasets were already balanced or when researchers sought to preserve sufficient training samples for complex models. For instance, Adnan et al. [56] employed a 70/15/15 split for their transformer-based fusion model on the large PARK Framework dataset, while Alshammri et al. [107] used a 70/30 split with SMOTE oversampling on the small Oxford dataset [43]. However, these approaches carry inherent risks. When applied to small datasets, particularly those with fewer than 50 subjects, the apparent performance gains may reflect overfitting rather than true generalizability. For example, Rehman et al. [88] reported 100% accuracy using a hybrid LSTM-GRU model on the Oxford dataset under random oversampling, but this result is unlikely to hold in larger, more heterogeneous cohorts. Similarly, Ali et al. [101] achieved 100% accuracy with an L1SVM-DNN cascade on the Oxford dataset, yet performance dropped to 96.42% on the larger Istanbul dataset, underscoring the fragility of high scores on small benchmarks. Another example, Velu and Jaisankar [108] achieved an AUC of 0.99 on the Istanbul PD Speech Dataset (N = 252, 188 PD), but this result must be interpreted in context; the dataset is highly imbalanced (75% PD, 25% HC), and evaluation used a single 80/20 train–test split without external validation. While the high AUC confirms excellent separation of classes within this specific cohort, it does not necessarily imply generalizability.

Nine studies [17,29,30,35,56,59,90,109] explicitly evaluated their models on independent, external datasets, a clear underrepresentation given the importance of real-world generalizability. Hireš et al. [90] conducted cross-dataset testing across CzechPD, PC-GITA, ItalianPVS, and RMIT-PD, finding that both XGBoost and CNN models experienced dramatic performance drops (accuracy falling to 33–74%) when tested outside their training corpus. This confirms that models trained on one dataset often fail to generalize to others due to differences in recording conditions, language, demographics, and task design. Federated learning, a privacy-preserving approach that trains models across decentralized datasets without sharing raw data, was also explored in a study by Quan et al. [17] using the FedOcw framework. These works demonstrated that dynamic client weighting can improve cross-lingual performance, but even federated models showed reduced performance (≈50–71%) when evaluated across linguistically distant datasets, highlighting persistent challenges in cross-dataset alignment. A smaller (n = 8) yet methodologically distinct set [26,49,58,84,94,110,111] implemented subject-independent validation, including LOSO or speaker-excluded designs, which better approximate real-world generalizability. For example, Alalayah et al. [84] reported 99% accuracy using RF with LOSO CV, while Qasim et al. achieved 98.2% accuracy after reducing 753 features to 18 via RFE and PCA. Nested or multi-stage validation, used in only three studies [30,35,56], offered a more rigorous approach by incorporating inner loops for hyperparameter tuning and feature selection, followed by outer loops for unbiased performance estimation. Favaro et al. [30] employed nested CV across six multilingual datasets, reporting mean F1 scores of 85% for multi-lingual tasks and 79% for cross-lingual tasks, providing a more realistic benchmark for real-world deployment. One study [11] stood out for its repeated/aggregated validation approach, using ensemble voting over numerous random subsamples.

Note that performance was primarily assessed using accuracy, precision, recall, F1-score, and AUC. Accuracy measures the overall correctness of predictions, while recall and precision assess the identification and correctness of positive cases, respectively. The F1-score balances precision and recall. Reported accuracies ranged widely, from 68.56% for the CNN + MLP end-to-end system [112] to 100% for classical ML models like RF or SVM on small datasets [84]. However, these near-perfect scores should be interpreted with caution, as mentioned earlier, they frequently originate from small, homogeneous datasets and are not accompanied by external validation. The best accuracy reported by reviewed studies is shown in Figure 9B for different model types. AUC evaluates a model’s ability to distinguish between classes and is especially reliable in imbalanced or diverse datasets. In PD studies, AUC was not favored over accuracy, with 38 studies reporting values between 0.73, as reported from a study using a generative model [79], and 1.00 based on the classical ML model [113], highlighting its value in assessing model quality.

Interpretability for clinical validation remains a critical yet underdeveloped aspect in voice-based PD detection models [114]. While the majority of high-performing studies rely on the black-box nature of ML and DL models, only a limited number integrate formal explainability methods to support clinical trust and adoption. Notable exceptions include the use of Shapley Additive Explanations (SHAPs) [115] in studies by Momeni et al. [68], Velu & Jaisankar [108], and Xu et al. [116], which identified clinically meaningful features like jitter, shimmer, and monotone speech patterns as key predictors, aligning with known PD-related dysarthria. Grad-CAM visualizations in Sedigh Malekroodi et al. [82] further localized decision-relevant segments within speech utterances, while Gimeno-Gómez et al. [35] introduced an interpretable cross-attention framework that fused self-supervised embeddings with 35 clinician-informed acoustic features, enabling transparent, multilingual PD detection. Despite these advances, most models still lack mechanisms to trace predictions back to pathophysiological speech impairments, and clinical validation of interpretability outputs remains rare.

## 4. Discussion

This systematic review of 69 studies reveals a rapidly evolving landscape in voice-based PD detection, marked by a near-equal split between traditional ML and DL approaches, and emphasis on hybrid and self-supervised paradigms. While high in-sample accuracies demonstrate the potential of vocal biomarkers for early screening, a critical gap persists between controlled experimental settings and real-world clinical applicability. The following discussion synthesizes key findings across feature representation, model architecture, dataset limitations, and outlines a path toward robust, generalizable, and clinically meaningful voice-based PD detection systems.

### 4.1. Dataset Design, Recording Variability, and Accessibility

**Figure 12:**
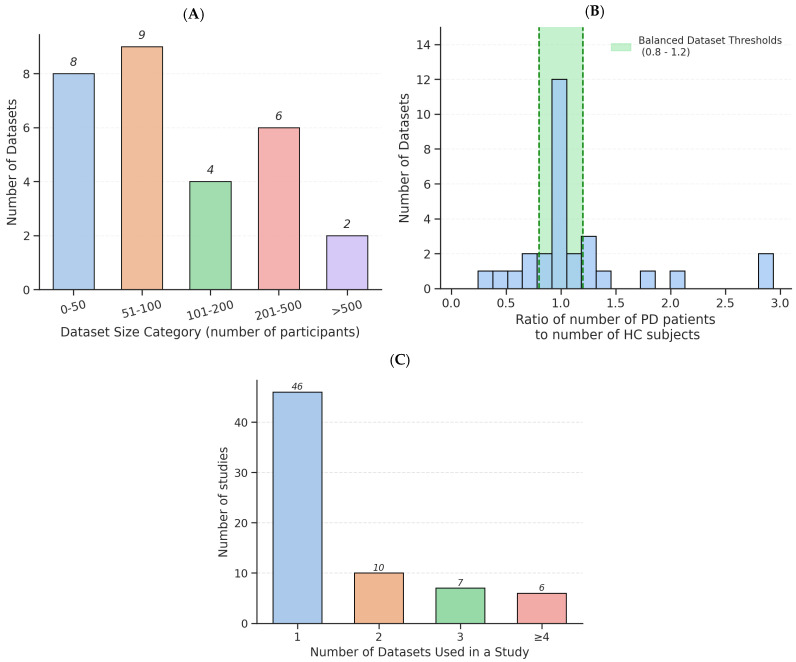
(**A**) Distribution of datasets by participant size category, showing that most datasets contain fewer than 100 participants. (**B**) Distribution of the ratio of PD patients to HC across datasets. The green shaded area (0.8–1.2) indicates the threshold for balanced datasets. (**C**) The number of studies by the number of datasets used shows that the majority relied on a single dataset.

The design, recording conditions, and accessibility of PD speech datasets reveal a field shaped by competing priorities between clinical depth, real-world applicability, and open science. Reviewed datasets differ markedly in speech task complexity and clinical annotation. High-quality collections such as PC-GITA [41], FraLusoPark [54], and NeuroVoz [53] employ linguistically rich protocols, ranging from sustained vowels and diadochokinetic syllables to reading passages, spontaneous monologues, and prosody-specific sentences, often developed in collaboration with speech-language pathologists and neurologists to capture dysarthria across phonatory, articulatory, and prosodic dimensions. In contrast, large-scale mobile or telemonitoring datasets like mPower [57], PARK Framework [56], and Telephone PD Voice Dataset (UAMS) [46] favor simpler tasks, such as a single vowel or short sentence, to enhance participant compliance and scalability, albeit at the cost of reduced sensitivity to subtle or context-dependent impairments. Clinical metadata follow a similar pattern, with some datasets containing detailed clinical scores and medication states, while others depend on self-reported diagnoses or lack key variables. As shown in Figure 12A, most datasets include fewer than 100 participants, highlighting a persistent trade-off: smaller studies offer richer clinical detail, whereas larger ones prioritize scale over annotation depth and task diversity.

This trade-off extends to recording environments, which span a spectrum from acoustically controlled clinical settings to uncontrolled real-world contexts. Datasets such as PC-GITA [41] and ItalianPVS [44] used professional microphones in quiet or sound-treated rooms, ensuring high signal fidelity. Conversely, mobile- and telephone-based collections [47,57,65,97] rely on built-in device microphones in ambient conditions, introducing variability from background noise, microphone quality, and speaker distance. While this shift supports remote monitoring and real-world relevance, it challenges model robustness and cross-dataset generalization. The ICEBERG dataset [11] stands out by providing parallel recordings under both high-fidelity and telephone conditions, offering rare insight into device-related performance gaps. However, inconsistent reporting of signal-to-noise ratios, preprocessing methods, or microphone specifications continues to impede systematic evaluation.

Limited dataset accessibility further compounds these issues. Despite growing emphasis on open science, many valuable resources, including PC-GITA [41], FraLusoPark [54], ICEBERG, and nearly all Chinese [17,25,33,60], Korean [58,59], and CzechPD [52] datasets, remain restricted or available only upon request. Publicly accessible datasets are largely limited to legacy UCI repositories that provide only extracted features [42,43] or a handful of newer collections offering raw audio [44,45,47,53,97]. Critically, several studies, including the Lithuanian PD Speech Dataset [64], PARK Framework [56], and various Chinese [17,25,33,60] and Italian [49,50,66] datasets, share only precomputed features or de-identified summaries, restricting independent validation, feature re-engineering, and multimodal analysis. This access bottleneck hinders reproducibility and cross-study comparisons and limits the development of standardized benchmarks [49,90].

The dataset used greatly affects both accuracy and generalizability. Controlled and homogeneous datasets such as the Oxford [43] or Istanbul PD Speech [42] datasets often yield very high accuracies due to limited variability, yet these models rarely generalize to new speakers or environments. In contrast, larger and more diverse datasets like PC-GITA [41] and PARK Framework [56] provide more realistic variability that slightly lowers accuracy but improves robustness across languages, microphones, and demographics. This highlights that dataset diversity, not only algorithm choice, is key to building clinically reliable voice-based PD detection systems.

### 4.2. Advancements in Machine Learning

Early studies predominantly relied on traditional ML pipelines, where handcrafted acoustic features were fed into classifiers like SVMs, RF, or logistic regression. These approaches remain prevalent, particularly in studies using small, clinically rich datasets where interpretability and feature transparency are prioritized [31,84,85,86,108,117,118,119,120]. However, this strength becomes a liability in heterogeneous settings, as models trained in controlled environments show sharp performance drops on home recordings, revealing the fragility of handcrafted features to recording conditions, noise, and demographics [49,90].

In recent years, however, there has been a noticeable shift toward deep learning architectures, including CNNs, RNNs, and hybrid models, which can automatically learn representations from raw or minimally processed audio. Such models are more commonly applied to larger, less clinically annotated datasets, where the abundance of data compensates for limited metadata and enables end-to-end training [32,56,81]. In contrast, deep architectures learn hierarchical and highly nonlinear feature representations that often operate as black boxes. The inability to trace how specific acoustic features contribute to model outputs limits clinician trust and hinders regulatory approval for AI-assisted diagnostic tools [30,36]. To bridge this divide, a growing number of studies [62,101,102] propose hybrid architectures that combine the best of both paradigms. These hybrid approaches demonstrate that feature refinement and representation learning need not be mutually exclusive; instead, they can be integrated to balance discriminative power, robustness, and clinical interpretability.

Concurrently, the field is shifting toward foundation models [30,35,56,82]. These models, pretrained on thousands of hours of unlabeled general-domain speech, can be fine-tuned on modest clinical datasets to achieve strong discriminative performance without manual feature engineering [74,75]. Dao et al. [36] demonstrated this potential using an ensemble of fine-tuned Wav2Vec 2.0, Whisper, and SeamlessM4T on the ICEBERG dataset [11], reporting an AUROC of 91.35% under subject-independent 5-fold cross-validation. Similarly, Sedigh Malekroodi et al. [82] achieved an AUROC of 0.92 with Wav2Vec 2.0 enhanced by supervised contrastive learning on the NeuroVoz dataset, outperforming traditional acoustic features. Despite their promise, foundation models have key clinical limitations. Their embeddings lack interpretability, making it difficult to identify which vocal biomarkers drive predictions, critical for clinician trust [30]. Moreover, most studies fine-tuned only the top layers of foundation models due to computational limits, which may hinder adaptation to pathological speech. Without careful domain adaptation and robust validation, these models risk overfitting or failing to generalize, especially when the target PD dataset differs acoustically or linguistically from the pretraining data. While foundation models enable cross-lingual PD detection, their clinical deployment depends on improving interpretability, addressing data scarcity, and ensuring rigorous validation.

### 4.3. Methodological and Reporting Limitations

A critical limitation across the current literature is the pervasive reliance on internal validation strategies that fail to reflect real-world deployment conditions. Of the 69 reviewed studies, approximately 70% employed internal methods, such as k-fold or fixed train–test splits, while less than 15% of studies conducted external or cross-dataset validation. This overreliance on internal evaluation inflates confidence in model performance [36,90]. Without subject-independent or external validation, reported metrics may reflect overfitting to dataset-specific artifacts, such as recording equipment, language, or demographic composition, rather than genuine disease-related biomarkers.

Demographic bias further limits fairness and external validity. Most datasets are heavily skewed toward older males; for example, the Istanbul PD dataset includes 188 male versus only 64 female participants. Nevertheless, only a few studies explicitly evaluate model performance across demographic subgroups [29,33,36,56,68,103,121]. Momeni et al. [68] demonstrated that by applying group-wise normalization based on age and gender in the mPower dataset [57], they improved classification accuracy by 9.5%, highlighting that demographic factors are not mere confounders but physiologically meaningful variables that must be explicitly modeled. Klempíř et al. [103] reported that Wav2Vec embeddings generalized well across languages but performed poorly on gender-imbalanced subsets.

Class imbalance can be considered another issue across studies, as shown in Figure 12B, with some clustering around a PD-to-HC ratio near 1.0 but almost half within the balanced range (0.8–1.2). Many datasets are heavily skewed [42,43,44,56,57], leading researchers to use synthetic oversampling to compensate. However, while such methods can inflate accuracy, they often mask weak real-world performance, particularly low specificity. For example, Hawi et al. [10] achieved 88.8% accuracy using Random Forest with SMOTE on the Istanbul dataset but only 71.1% specificity, indicating a high false-positive rate and limited clinical reliability. Similar trends in studies by Alshammri et al. [107], Rehman et al. [88], and Velu & Jaisankar [108] show that high reported accuracy often coincides with poor sensitivity–specificity balance, raising concerns about the robustness of oversampled evaluations.

Of all reviewed studies, as shown in Figure 12C, almost two-thirds rely exclusively on a single dataset, most commonly the small, homogeneous. Ten studies used two datasets [33,49,55,58,96,101,102,111,113,122], seven used three [62,68,83,94,103,123,124], and only six studies (Hireš et al. [90]; Scimeca et al. [50], Favaro et al. [30]; Ibarra et al. [29]; Quan et al. [17]; Gimeno-Gómez et al. [35]) performed rigorous cross-dataset validation using four or more datasets. This heavy reliance on single datasets severely constrains generalizability. These studies illustrated the consequences; models achieving >90% accuracy within individual datasets saw performance collapse when evaluated across datasets, revealing that many high-performing systems are effectively overfit to dataset-specific artifacts rather than true disease biomarkers. However, even these multi-dataset studies faced challenges due to heterogeneity in recording protocols, medication state, and task design, which introduced bias and reduced performance in cross-dataset scenarios.

Furthermore, most datasets are monolingual, predominantly in English, Spanish, or Italian. This linguistic homogeneity introduces language-specific biases, as articulatory patterns and phonetic structures vary across languages. For example, Wang et al. [25] found that articulation features (F1, F2, BBE, and MFCC) were more predictive than phonation features in Mandarin-speaking PD patients, while Favaro et al. [30] demonstrated that non-interpretable embeddings outperformed interpretable features in cross-lingual experiments.

Across the reviewed studies, model performance was primarily evaluated using accuracy, F1-score, AUC, sensitivity, and specificity, reflecting the models’ classification precision and clinical discriminative power. Despite high in-sample accuracies, several recurring challenges were observed, including dataset imbalance, small participant size, limited cross-dataset validation, and inconsistent metric reporting. These gaps highlight the need for standardized evaluation frameworks and external benchmarking to improve the reliability and generalizability of voice-based Parkinson’s detection models.

Compounding these issues is a striking lack of standardization across the field. There is no consensus on speech tasks (e.g., sustained /a/ vs. DDK vs. free speech), feature sets (e.g., MFCCs vs. RPDE vs. Wav2Vec embeddings), or performance metrics (e.g., accuracy vs. AUROC vs. F1), making cross-study comparisons difficult and reproducibility elusive. For instance, while some studies report AUC to account for imbalance [36,68], others rely solely on accuracy, even on highly skewed datasets [84,88]. Similarly, validation protocols vary widely, from LOSO to single 70/30 splits, with little justification for the chosen approach. This methodological heterogeneity fragments the literature and impedes the establishment of reliable benchmarks, ultimately slowing progress toward clinically deployable systems. Additionally, most studies reported only mean or best-run results without confidence intervals, which limits statistical comparability and hinders meaningful cross-study comparison. Future work should report variance, fold-wise averages, and task-specific analyses.

Addressing these gaps requires community-wide adoption of standardized protocols, including mandatory external validation, transparent reporting of sensitivity–specificity trade-offs, and shared task definitions, to ensure that advances in voice-based PD translate into trustworthy, real-world tools.

### 4.4. Limitations of the Current Review

This review, while comprehensive, has several limitations. It was conducted without a pre-registered protocol, which may affect reproducibility. It primarily focuses on binary classification tasks (PD vs. HC), with limited coverage of studies on disease staging, progression tracking, or differential diagnosis. The inclusion of only English-language, full-text studies may have excluded valuable research in other languages, introducing linguistic and geographic bias. While the review adhered to PRISMA 2020 guidelines, it did not include a formal meta-analysis due to the high heterogeneity of datasets, model architectures, and evaluation metrics among studies. Consequently, the results emphasize qualitative trends rather than quantitative effect sizes. Moreover, the literature search was limited to four databases and specific search terms, which might have narrowed the study coverage. Performance metrics across studies are not fully comparable due to heterogeneous validation protocols, dataset sizes, and class distributions, with many relying solely on internal validation. Finally, the emphasis on sustained phonation tasks may not capture the full complexity of spontaneous or conversational speech in real-world scenarios.

### 4.5. Future Directions

Advancing voice-based PD detection toward real-world clinical utility requires addressing key challenges in scalability, generalizability, and regulatory integration. Many studies report high in-sample accuracy but rely on small, controlled datasets lacking external validation, which limits generalizability. Models trained on clean recordings often degrade in real-world conditions due to background noise, variable microphone quality, and uncontrolled acoustics. Most studies use cross-sectional data, single snapshots that cannot capture individual disease trajectories, missing opportunities for longitudinal tracking of progression or treatment response. Current research is also constrained by demographic and linguistic biases. Most datasets overrepresent a few languages (English, Spanish, Italian), and subgroup metrics by gender, age, or language are rarely reported, limiting fairness and cross-population evaluation. Compounding these issues is the scarcity of large-scale, publicly available, and clinically annotated speech datasets, which fragments the field and impedes reproducible benchmarking. To move toward clinically viable systems, future research must prioritize standardized validation protocols, including subject-independent and external testing, develop multilingual and multi-environment datasets that reflect real-world diversity, adopt fairness- and diversity-aware modeling practices with standardized reporting and subgroup analysis, and establish open-access benchmarks with shared tasks and evaluation metrics. Crucially, beyond performance metrics, the lack of interpretable models remains a major barrier; clinicians need transparency into why a prediction is made to trust, validate, and act on it. Moreover, integrating complementary modalities, such as applying deep learning to map speech signals to 3D facial expressions, could further enhance interpretability and offer visual biomarkers of motor decline [125,126].

Overall, Voice-based PD detection has shown technical maturity but limited clinical adoption. The field should shift from maximizing in-sample accuracy to ensuring real-world robustness, fairness, and utility. Only through such coordinated efforts can it evolve from a promising research prototype into a reliable, scalable, and equitable tool for both clinical and at-home use.

## 5. Conclusions

This systematic review highlights the shift from traditional ML to DL models for voice-based PD detection. While these models show strong performance in controlled environments, they struggle to generalize across languages, datasets, and real-world conditions. Key challenges include a lack of diverse, publicly accessible speech datasets and inconsistent evaluation standards. To enable clinical use, future research needs standardized testing frameworks, fairness-aware models to address demographic diversity, and explainability methods to build clinician trust. These improvements are essential for making voice-based PD detection reliable and effective in practice. Finally, AI-driven voice analysis holds great promise as a cost-effective, accessible, and non-invasive tool for early PD detection and longitudinal disease monitoring, provided methodological rigor and interpretability remain at the forefront of innovation.

## Data Availability

No original data were produced in this study.

## Supplementary Materials

The following supporting information can be downloaded: https://www.mdpi.com/article/10.3390/bioengineering12111279/s1, Table S1: Detailed search queries applied in each database for article retrieval.; Table S2: Summary of Parkinson’s speech datasets reviewed.; Table S3: Summary of Methods and Findings from the reviewed studies.

## Abbreviations

The following abbreviations are used in this manuscript:

AI	Artificial Intelligence
ASR	Automatic Speech Recognition
AST	Audio Spectrogram Transformer
AUC/AUROC	Area Under the Receiver Operating Characteristic Curve
CNN	Convolutional Neural Network
CV	Cross-Validation
DDK	Diadochokinetic
DL	Deep Learning
GAN	Generative Adversarial Network
Grad-CAM	Gradient-weighted Class Activation Mapping
GRU	Gated Recurrent Unit
HC	Healthy Control
HNR	Harmonics-to-Noise Ratio
H&Y	Hoehn & Yahr scale
KNN (k-NN)	k-Nearest Neighbors
LOOCV	Leave-One-Out Cross-Validation
LOSO	Leave-One-Subject-Out
LSTM	Long Short-Term Memory
MFCCs	Mel-Frequency Cepstral Coefficients
ML	Machine Learning
PCA	Principal Component Analysis
PD	Parkinson’s Disease
RF	Random Forest
RFE	Recursive Feature Elimination
RNN	Recurrent Neural Network
SHAP	Shapley Additive Explanations
SLT	Superlet Transform
SMOTE	Synthetic Minority Oversampling Technique
SVM	Support Vector Machine
UPDRS/MDS-UPDRS	(Movement Disorder Society)-Unified Parkinson’s Disease Rating Scale
Wav2Vec 2.0	Self-supervised speech model developed by Facebook AI
