# Supplementary material for: Voice-Based Detection of Parkinson’s Disease Using Machine and Deep Learning Approaches: A Systematic Review
Source: Bioengineering (Basel). 2025 Nov 20;12(11):1279. doi: 10.3390/bioengineering12111279 (PMC12649940; doi:10.3390/bioengineering12111279)
Supplement: Supplementary file 1 [file bioengineering-12-01279-s001.zip › bioengineering-3983979-supplementary.pdf]

Review

Voice-Based Detection of Parkinson's Disease Using Machine and Deep Learning Approaches: A Systematic Review

Hadi Sedigh Malekroodi [1], Byeong-il Lee [1,2,3,*] and Myunggi Yi [1,2,4,*]

[1] Industry 4.0 Convergence Bionics Engineering, Pukyong National University, Busan 48513, Republic of Korea; hadi_sedigh@pukyong.ac.kr
[2] Digital Healthcare Research Center, College of Information Technology and Convergence, Pukyong National University, Busan 48513, Republic of Korea
[3] Major of Human Bioconvergence, Division of Smart Healthcare, Pukyong National University, Busan 48513, Republic of Korea
[4] Major of Biomedical Engineering, Division of Smart Healthcare, Pukyong National University, Busan 48513, Republic of Korea
* Correspondence: bilee@pknu.ac.kr (B.-i.L.); myunggi@pknu.ac.kr (M.Y.)

Supplementary materials include:

Table S1. Detailed search queries applied in each database for article retrieval. Searches were conducted on September 7, 2025, across PubMed, Web of Science, IEEE Xplore, and Scopus.
Table S2. Summary of Parkinson's speech datasets reviewed.
Table S3. Summary of Methods and Findings from the reviewed studies

Table S1. Detailed search queries applied in each database for article retrieval. Searches were conducted on September 7, 2025, across PubMed, Web of Science, IEEE Xplore, and Scopus.

Source	Search Query Used
PubMED	("Parkinson* disease"[tiab] OR Parkinsonism[tiab] OR PD[tiab])AND (speech[tiab] OR voice[tiab] OR vocal*[tiab] OR "voice signal*"[tiab] OR acoustic*[tiab])AND ("machine learning"[tiab] OR "deep learning"[tiab] OR "artificial intelligence"[tiab] OR AI[tiab] OR "neural network*"[tiab]) AND(detect*[tiab] OR diagnos*[tiab] OR classif*[tiab] OR screen*[tiab]) AND ("2020/01/01"[dp] : "2025/09/01"[dp])
Scopus	(TITLE-ABS-KEY ("Parkinson* disease" OR Parkinsonism OR PD) AND TITLE-ABS-KEY (speech OR voice OR vocal* OR "voice signal*" OR acoustic*) AND TITLE-ABS-KEY ("machine learning" OR "deep learning" OR "artificial intelligence" OR AI OR "neural network*") AND TITLE-ABS-KEY (detect* OR diagnos* OR classif* OR screen*)) AND PUBYEAR > 2019 AND PUBYEAR < 2026 AND (LIMIT-TO (LANGUAGE,"English"))
IEEE Xplore	("All Metadata":Parkinson* OR "All Metadata":Parkinsonism OR "All Metadata":PD)AND ("All Metadata":speech OR "All Metadata":voice OR "All Metadata":vocal* OR "All Metadata":"voice signal*" OR "All Metadata":acoustic*)AND ("All Metadata":"machine learning" OR "All Metadata":machine-learn* OR "All Metadata":"deep learning" OR "All Metadata":deep-learn* OR "All Metadata":"artificial intelligence" OR "All Metadata":AI OR "All Metadata":"neural network*") AND ("All Metadata":detect* OR "All Metadata":diagnos* OR "All Metadata":classif* OR "All Metadata":screen*) AND (Publication Year:2020-2025)
Web of Science	("Parkinson* disease" OR Parkinsonism OR PD) (All Fields) and (speech OR voice OR vocal* OR "voice signal*" OR acoustic*) (All Fields) and ("machine learning" OR "deep learning" OR "artificial intelligence" OR AI OR "neural network*") (All Fields) and (detect* OR diagnos* OR classif* OR screen*) (All Fields)

Table S2. Summary of Parkinson's speech datasets reviewed.

Dataset Name	Recordings & Participants	Language	PD∣HC	Gender Distribution	Age Range
**PC-GITA [1]**	6300 rec, 100 p	Spanish (Colombian)	50 ∣50	25M/25F PD, 25M/25F HC	PD: M 61.5±11.6, F 60.7±7.3 / HC: M 60.5±11.6, F 61.4±7.0
**PARK Framework Dataset [2]**	1854 rec, 1306 p	English	392∣ 914	218M/171F/1NB/2Unk PD, 390M/524F HC	PD: 62.2±12.7 / HC: Not reported
**Parkinson Dataset with Replicated Acoustic Features-UCI [3]**	240 rec, 80 p (3×)	Spanish	40 ∣ 40	22M/18F PD, 27M/13F HC	PD: 69.58±7.82 / HC: 66.38±8.38
**Istanbul PD Speech Dataset-UCI [4]**	756 rec, 252 p	Turkish	188∣ 64	107M/81F PD, 23M/41F HC	PD: 65.1±10.9 / HC: 61.1±8.9
**Oxford PD Speech Dataset-UCI [5]**	195 rec, 31 p	English	23 ∣ 8	16M/7F PD, 3M/5F HC	PD: 65.8±9.8 / HC: Not reported
**Parkinson's Telemonitoring Dataset-UCI [6]**	5875 rec, 42 p	English	20 ∣ 22	15M/5F PD, 11M/11F HC	PD: 65.0±9.8 / HC: 62.0±10.1
**NeuroVoz Corpus [7]**	2977 rec, 112 p	Spanish + Castilian	54 ∣ 58	34M/20F PD, 27M/31F HC (+1 sex not reported)	PD: M 71.9±11.8, F 70.8±8.0 / HC: M 61.6±7.4, F 66.4±12.3
**Italian Parkinson's Voice and Speech [8,9]**	845 rec, 65 p	Italian	28∣ 37 (22 HC + 15 YHC)	19M/9F PD, 10M/12F HC, 13M/2F YHC	PD: 62.3±10.5 / HC: 63.1±12.2
**mPower Dataset [10]**	65022 rec, 5826* p	English	965∣3952	629M/336F PD, 3205M/747F HC	PD: Not reported / HC: Not reported
**Taiwanese Mandarin PD Speech Dataset [11]**	360 rec, 360 p	Taiwanese	186∣174	62M/124F PD, 61M/113F HC	PD: 71.2±7.2 / HC: 44.0±17.5
**Korean PD Speech Dataset [11,12]**	2068 rec, 291 p	Korean	161∣125	64M/97F PD, 23M/40F HC	Early PD: 65.7±9.8 / HC: 60.1±15.7 ; Advanced PD: 71.2±7.2 / HC: 44.0±17.5
**NeuroLogical Signals (NLS) Dataset [13]**	50 p	English	23∣27	14M/9F PD, 11M/16F HC	PD: 67.16 / HC: 64.44
**FraLusoPark Dataset [14]**	140 p	French + European Portuguese	75∣65	37M/38F PD, 31M/34F HC	PD: M 66.9±8.5, F 64.6±11.9 / HC: M 66.9±14.4, F 62.4±12.4
**German PD Speech Dataset [15]**	176 p	German	88∣88	47M/41F PD, 44M/44F HC	PD: M 66.7±8.7, F 67.2±9.7 / HC: M 63.8±12.7, F 62.6±15.2
**Czech PD Speech Dataset [16]**	100 p	Czech	50∣50	30M/20F PD, 30M/20F HC	PD: M 65.6±9.6, F 60.1±8.7 / HC: M 60.3±11.5, F 63.7±10.8
**Czech PD-DDK Dataset [17]**	120 rec, 60 p	Czech	30∣30	30M/0F PD, 30M/0F HC	PD / HC: Age matched (details not reported)
**Czech PD-APS Voice Dataset [18]**	44 rec, 44 p	Czech	22∣22	10M/12F PD, 11M/11F HC	PD: 65.40±9.10 / HC: 54.50±17.70
**ICEBERG Cohort Speech Dataset [19]**	221 p (206 HQ mic, 162 tel)	French	121∣100	74M/41F PD, 48M/43F HC (high-quality mic); 63M/38F PD, 36M/25F HC (telephone)	PD: 63.8±9.3 / HC: 59.1±10.0 (high-quality mic); PD: 63.5±9.0 / HC: 62.6±8.5 (telephone)
**Telephone PD Voice Dataset (UAMS) [20]**	81 p	English	40∣41	21M/19F PD, 16M/24F HC	PD: 66.6±9.0 / HC: 47.9±14.5
**Lithuanian PD Speech Dataset [21]**	592 rec, 99 p	Lithuanian	64∣35	30M/34F PD, 11M/24F HC	PD: Not reported / HC: Not reported
**MDVR-KCL Dataset [22]**	37 p	English	16∣21	9M/7F PD, 19M/2F HC	PD: Not reported / HC: Not reported
**RMIT-Smartphone Phoneme Dataset [23]**	72 p	English + (phoneme-based, language-independent)	36∣36	20M/16F PD, 18M/18F HC	PD: 68.2±7.5 / HC: 67.9±6.9
**Mandarin PD Speech Dataset [24]**	1200 rec, 100 p	Mandarin Chinese	50∣50	25M/25F PD, 25M/25F HC	PD: 63.57±11.31 / HC: Not reported
**Chinese Mild-PD Voice Dataset [25]**	278 p (139 PD/139 HC)	Mandarin Chinese	139∣139	67M/72F PD, 58M/81F HC	PD: 65.96±7.55 / HC: 64.70±9.54
**Chinese PD Speech Dataset (GYENNO) [26,27]**	266 rec, 45 p (132 /a/, 134 sent; 30 PD/15 HC)	Mandarin Chinese	30∣15	16M/14F PD, 7M/8F HC	PD / HC: 37–75 years (mean±SD not reported)
**Italian Torino corpus-1 [28,29]**	330 rec, 30 p	Italian	17∣13	12M/5F PD, 8M/5F HC	PD: 70.35±7.23 / HC: 63.62±5.80
**Italian Torino corpus-2 [28,29]**	30 rec, 30 p	Italian	15∣15	11M/4F PD, 11M/4F HC	PD: 70.38±7.7 / HC: 59.93±15.15
**Italian Torino corpus-3 [28]**	44 p	Italian	26∣18	Gender not reported	PD: 71.7±7.39 / HC: 65.5±8.42
**Italian Rome corpus [30]**	426 p	Italian	160 ∣ 266	Gender not reported (groups age-, gender-, and BMI-matched)	PD / HC: Age and BMI matched (numerical values not reported)
**Spanish PD Vowel Dataset [31]**	119 p (55 PD/64 HC)	Spanish	18∣64	31M/24F PD, gender not reported HC	PD / HC: 38–79 years (mean±SD not reported)

Table S2. (Continue of columns)

Dataset Name	Voice Task(s) & Duration	Noise Env / Setup	Device & Format	Annotation Method	Source / Institution	Publicly available?	Used?
**PC-GITA [1]**	sustained vowels (/a/, /e/, /i/, /o/, /u/), pitch glides, diadochokinetic tasks (/pa/, /ta/, /ka/, /pa-ta-ka/ etc.), 45 words, 10 sentences, phonetically balanced dialogue, emphasized sentences, reading text, spontaneous monologue	Soundproof booth (s), Real-world €	Shure SM63L dynamic omnidirectional microphone + M-Audio Fast Track C400 audio card, WAV, 44.1 kHz, 16-bit	Clinical diagnosis with neurologist-confirmed	Universidad de Antioquia + Friedrich-Alexander-Universität Erlangen-Nürnberg	No, Available upon request from the authors	19
**PARK Framework Dataset [2]**	English pangram "The quick brown fox jumps over the lazy dog" ~3–5 seconds (pangram utterance)	home environment, clinical setup, PD care facility (varied noise)	iPhone/laptop/varied webcams WAV, 16 kHz	Clinical domain (URMC + InMotion cohorts), self-reported (home cohort)	University of Rochester + InMotion PD Care Facility + parktest.net	No (only de-identified features available on GitHub)	1
**Parkinson Dataset with Replicated Acoustic Features-UCI [3]**	Sustained vowel /a/ (3 repetitions per subject), at least 5 seconds and on one breath	Controlled acoustic environment (quiet room, clinical/lab setting)	Headband microphone (AKG 520) with cardioid pattern + TASCAM US322 external sound card connected to a laptop;44.1 kHz, 16-bit	Clinical domain — subjects diagnosed by neurologists; PD participants recruited from Regional Association for Parkinson's Disease in Extremadura; ethical approval obtained	Department of Mathematics & Department of Computer and Communication Technologies, University of Extremadura, Spain (hosted on UCI Machine Learning)	Yes (UCI ML Repository: https://archive.ics.uci.edu/dataset/489/parkinson%2Bdataset%2Bwith%2Breplicated%2Bacoustic%2Bfeatures)	2
**Istanbul PD Speech Dataset-UCI [4]**	Sustained vowel /a/ (3 repetitions per subject), Varied (~2–5 seconds per sample)	quiet clinical room	Trust MC-1500 microphone;WAV, 44.1 kHz	Clinical diagnosis with neurologist-confirmed UPDRS + Hoehn & Yahr staging	Department of Neurology, Cerrahpaşa Faculty of Medicine, Istanbul University, Turkey (in collaboration with Bahçeşehir University & Boğaziçi University) hosted on UCI Machine Learning Repository	Yes (UCI ML Repository: https://archive.ics.uci.edu/ml/datasets/parkinsons)	15
**Oxford PD Speech Dataset-UCI [5]**	Sustained vowel phonation (/a/ at comfortable pitch) ~6 phonation recordings per subject (average ≈ 6 seconds)	Sound-treated IAC acoustic booth, controlled environment	KG C420 head-mounted microphone (8 cm from lips); WAV, 44.1 kHz 16-bit	Clinical domain, confirmed PD diagnoses (Hoehn & Yahr stages 1–4, 0–28 years since diagnosis); dataset originally curated by Little et al. (2007, 2008)	University of Oxford / NCVS Parkinson's Voice Dataset; hosted on UCI Machine Learning Repository	Yes (UCI ML Repository: https://archive.ics.uci.edu/ml/datasets/parkinsons)	13
**Parkinson's Telemonitoring Dataset-UCI [6]**	Six sustained vowel /a/ phonations per session, Up to 30 seconds	Home acoustic environments — uncontrolled (telemonitoring condition), device contained signal-triggered capture to minimize noise	Intel At-Home Testing Device (AHTD) — included a head-mounted microphone (5 cm from lips). WAV, 24 kHz, 16-bit	Clinical telemonitoring domain — UPDRS (motor and total) scored at baseline, 3 months, and 6 months by neurologists; scores linearly interpolated between visits for regression mapping	University of Oxford / NCVS Parkinson's Voice Dataset, in collaboration with Intel Corporation (hosted on UCI Machine Learning Repository)	Yes (UCI Repository: https://archive.ics.uci.edu/ml/datasets/Parkinsons+Telemonitoring)	0*
**NeuroVoz Corpus [7]**	sustained vowels (/a/, /e/, /i/, /o/, /u/), diadochokinetic /pa-ta-ka/, 16 Listen-and-Repeat sentences, free spontaneous monologue, Sustained vowels: ~3.9 s PD / 4.4 s HC; LR sentences: ~11 s PD / 13 s HC; DDK: ~3.3 s PD / 3.7 s HC; Monologues: ~31 s PD / 47 s HC	quiet clinical room (non-acoustically isolated)	AKG C420 headset microphone + Sound Blaster Live sound card, WAV, 44.1 kHz	Clinical diagnosis by neurologists (HGUGM/HUF), UPDRS + Hoehn & Yahr staging, GRBAS perceptual evaluation	Universidad Politécnica de Madrid (UPM) + Hospital General Universitario Gregorio Marañón (HGUGM) + Hospital Universitario de Fuenlabrada (HUF), Madrid, Spain	Yes Upon request (Zenodo: https://doi.org/10.5281/zenodo.10777656)	4

Dataset Name	Voice Task(s) & Duration	Noise Env / Setup	Device & Format	Annotation Method	Source / Institution	Publicly available?	Used?
**Italian Parkinson's Voice and Speech [8,9]**	sustained vowels (/a/, /e/, /i/, /o/, /u/), diadochokinetic /pa/, /ta/, phonemically balanced text, phonemically balanced words and phrases, Varied: sustained vowels ~5 s, text/phrases ~70–95 s	quiet echo-free room, ~22°C	Laptop/PC with external microphone (15–25 cm distance), WAV, Not reported kHz	Clinical diagnosis by neurologists; severity rated with UPDRS and Hoehn & Yahr staging	University of Bari "Aldo Moro" + Polytechnic University of Bari + ASL Bari	Yes (data available on IEEE)	2
**mPower Dataset [10]**	sustained vowel /a/, 10 seconds (sustain vowel phonation task)	Uncontrolled real-world conditions (home, work, outdoors); no acoustic filtering during collection	iPhone 4S or newer (iOS ≥8), M4A (iPhone mic), 44.1 kHz	Self-reported PD diagnosis (via app survey); subset validated with MDS-UPDRS + PDQ-8 questionnaires	Sage Bionetworks + University of Rochester Medical Center	Yes (Request in Synapse: https://www.synapse.org/mPower)	4
**Taiwanese Mandarin PD Speech Dataset [11]**	Standardized 500-character article reading, Reading a sentence (~2–3 min)	quiet clinic rooms	Smartphone microphone (5–35 cm distance) WAV (PCM), 44.1 kHz, 16-bit	Clinical diagnosis by neurologists using UK PD Society Brain Bank Criteria; Hoehn & Yahr staging; ON-medication phase	National Taiwan University Hospital, Taiwan	No (data not public, but mentioned available on request)	1
**Korean PD Speech Dataset [11,12]**	reading tasks; sustained vowels; syllable repetition (/pa-ta-ka/); sentence repetition; reading texts Short (<40 chars) and long (>40 chars)	quiet clinic rooms (not fully soundproof)	Smartphone microphone (5–35 cm distance) WAV (PCM), 44.1 kHz, 16-bit	Clinical diagnosis by neurologists using UK PD Society Brain Bank Criteria; Hoehn & Yahr staging; ON-medication phase	Seoul National University Hospital /Sangmyung University, Korea	No (data not public, mentioned available on request)	2
**NeuroLogical Signals (NLS) Dataset [13]**	Cookie Theft Picture description (SS), reading two short passages (RP), 60s limit for SS task; not reported for RP tasks	acoustically and visually controlled conditions	computer and microphone WAV format, 16 kHz, 16-bit, mono	Clinical diagnosis by neurologists using	Johns Hopkins Medicine (JHM), Baltimore, Maryland, USA	No (No Detail provided)	1
**FraLusoPark Dataset [14]**	Sustained vowel /a/ (≥5 s ×3), maximum phonation time, DDK /pataka/ (30 s), reading 10 words + 10 sentences, short text ("The North Wind and the Sun"), storytelling ("Frog, Where are you?"), prosody-specific sentences, 3-min free conversation	Quiet room, clinical setting (controlled acoustic environment)	EVA2 system (SQLab, Aix-en-Provence, France) + Marantz PMD661 MKII recorder, head-mounted mic, WAV, 44.1 kHz, 16-bit	UK PD Brain Bank criteria, neurologist confirmed; subgroups stratified by disease duration and L-Dopa response; OFF vs ON medication states	Aix-Marseille University (France) + University of Lisbon (Portugal) + multiple clinical centers (Centre Hospitalier du Pays d'Aix, Hospital de Santa Maria Lisbon, Campus Neurológico Sénior)	No (data archived for preservation, future access intended via SLDR repository)	1
**German PD Speech Dataset [15]**	Spontaneous speech, phonetically rich text, question–answer pairs with stress, sentence reading, word reading, sustained vowel, rapid syllable repetition (/pa/, /pa-ta-ka/, /pa-pe pa-pi pa-po pa-pu/) Not fixed; multiple tasks 5–30 s each	Quiet room, low ambient noise	External condenser microphone, WAV, 16 kHz	Clinical neurologist diagnosis; PD severity scored by UPDRS-III (motor section)	University of Erlangen-Nürnberg + partners (per Bocklet et al., 2013)	No (used internally for research, not publicly hosted)	4
**Czech PD Speech Dataset [16]**	Rapid syllable repetition (/pa-ta-ka/ ≥7 per breath), spontaneous monologue (~90 s) /pa-ta-ka/: ~7–8 s; Monologue: ~2 min	Quiet room, controlled acoustic conditions (per dysarthria recording guidelines)	Head-mounted condenser mic (Beyerdynamic Opus 55, ~5 cm from mouth), WAV, 48 kHz 16-bit	Clinical diagnosis by neurologists, MDS-UPDRS III	Charles University Prague + collaborators (per Rios-Urrego et al., 2024)	No, upon request can be available	5
**Czech PD-DDK Dataset [17]**	Repetition of syllable /pa/ (DDK task) ~30 s per recording (2 per subject)	Clinical diagnosis confirmed by neurologists	Not reported, WAV, 48 kHz	Neurologist clinical diagnosis (criteria not detailed)	Czech Technical University in Prague (Faculty of Biomedical Engineering)	No	2
**Czech PD-APS Voice Dataset [18]**	Sustained vowels /A/, /I/ Not reported	Low ambient noise room	Headset condenser microphone (Beyerdynamic Opus 55, ~5 cm from lips) WAV, 48 kHz, 16-bit	PD diagnosed by Parkinson's Disease Society Brain Bank criteria; APS (MSA, PSP) diagnosed by consensus/NINDS criteria; neurologist confirmed, UPDRS/NNIPPS scored	Czech Technical University in Prague + Charles University, First Faculty of Medicine (Department of Neurology)	No	2

Dataset Name	Voice Task(s) & Duration	Noise Env / Setup	Device & Format	Annotation Method	Source / Institution	Publicly available?	Used?
**ICEBERG Cohort Speech Dataset [19]**	Quiet consultation rooms (ICM, hospital), home/office quiet rooms for HC; self-recorded telephone with IVM server Reading (1 min), sentence repetition (10–20 s), free speech (1 min), diadochokinesia (DDK, 1–1.5 min)		Beyerdynamic Opus 55 mkII head-mounted mic + Focusrite Scarlett 2i2 (96 kHz, 24-bit); Telephone (8 kHz, PCM 16, G711 codec)	UK Parkinson's Disease Society Brain Bank criteria, neurologist-confirmed, Hoehn & Yahr, MDS-UPDRS III, ICEBERG clinical trial (NCT02305147)	Paris Brain Institute (ICM), Sorbonne University, Inserm, CNRS, Hôpital Pitié-Salpêtrière, France	No	2
**Telephone PD Voice Dataset (UAMS) [20]**	Sustained vowel /a/, ~3 s (sustained vowel /a/)	Uncontrolled home/remote environments (telephone lines)	Telephone (voicemail system), WAV, 8 kHz, 16-bit	PD confirmed by movement disorders neurologist (UK Brain Bank + MDS diagnostic criteria); HC self-reported absence of neurological/speech disorders	University of Arkansas for Medical Sciences (UAMS), USA	Yes (Figshare DOI:10.6084/m9.figshare.23849127)	6
**Lithuanian PD Speech Dataset [21]**	(1) Sustained phonation — vowel /a/ voiced at comfortable pitch and loudness for at least 5 s × 3 repetitions.<br>(2) Text-dependent speech — a short phonetically balanced Lithuanian sentence: "turėjo senelė žilą oželį" ("Granny had a little greyish goat").<br>Sustained vowel /a/: ~5 s (repeated 3 times)<br>Sentence reading: ~2–4 s	Sound-proof booth, controlled acoustic conditions.	Acoustic cardioid microphone (AKG Perception 220) (frequency range 20–20,000 Hz)<br>Smartphone microphone (Samsung Galaxy Note 3)<br>Both positioned ~10 cm from the mouth WAV, 44.1 kHz 16-bit	Clinical diagnosis and recorded at Lithuanian University of Health Sciences.	Kaunas University of Technology (KTU), Lithuania & Lithuanian University of Health Sciences (Department of Otorhinolaryngology)	No, Only extracted feature sets (not raw audio) are provided as Supporting Information	1
**MDVR-KCL Dataset [22]**	Reading passage ("The North Wind and the Sun"), optional technical text snippet, spontaneous dialogue, ~2 min per subject	Quiet examination room (~10 m², ~500 ms reverberation time)	Motorola Moto G4 smartphone (Toggle Recording App), WAV, 44.1 kHz, 16-bit	Clinical diagnosis by neurologists; Hoehn & Yahr, UPDRS II-5, UPDRS III-18 ratings annotated	King's College London (KCL) Hospital, Denmark Hill, UK; Fraunhofer IAIS, Germany (i-PROGNOSIS project)	Yes (Zenodo DOI:10.5281/zenodo.2867216)	6
**RMIT-Smartphone Phoneme Dataset [23]**	Sustained phonemes /a/, /o/, /m/, Variable (~3–10 s sustained)	Typical clinical/office settings with ambient noise (~20 dB)	iPhone 6S Plus (built-in mic, default settings), WAV, 48.1 kHz, 16-bit	UK Parkinson's Disease Society Brain Bank criteria; neurologist-confirmed PD diagnosis (≤10 years since onset); exclusion of advanced PD symptoms	RMIT University + Monash Health (Melbourne, Australia)	No (available on request)	2
**Mandarin PD Speech Dataset [24]**	Sustained vowels (/a/, /e/ in Chinese Pinyin), tongue twisters ("si shi si zhi shi shi zi", "yi zhi da hua wan kou zhe yi zhi da hua ha ma") 6 s stable segments (sustained vowels), ~3–5 s per tongue twister	hospital/clinical environment, noise reduced using spectral subtraction	Rode NT-USB microphone (10 cm from mouth) WAV, 96 kHz	Clinical diagnosis by neurologists; (Hoehn & Yahr stage 1–3, MDS-UPDRS III motor score 36.43±17.39), ethics-approved	Huazhong University of Science and Technology (HUST) + Tongji Hospital + Gyenno Science Co., Ltd., China	No (authors state raw data available on request)	1
**Chinese Mild-PD Voice Dataset [25]**	Sustained vowels [a], [o], [i] at comfortable pitch and loudness, ≥6 s per sustained vowel, 3 repetitions each	Quiet consulting room (low ambient noise)	External condenser microphone coupled to smartphone (5 cm from mouth), WAV, 44.1 kHz, 16-bit	2015 MDS diagnostic criteria, confirmed by two movement disorder experts + DaT scan for all mild PD (mH&Y ≤1.5)	Chinese PLA General Hospital (Beijing) + Gyenno Science Co., Ltd. + HUST-GYENNO CNS Intelligent Digital Medicine Technology Center	No (authors state raw data available on request)	1
**Chinese PD Speech Dataset (GYENNO) [26,27]**	Sustained vowel /a/, short sentence "si shi si zhi shi shi zi" (Mandarin tongue twister) ~3–6 s sustained vowel; ~5–8 s sentence	Quiet room	Smartphone (within 10 cm from mouth), WAV, 96 kHz	Clinical diagnosis confirmed by two neurologists; Hoehn & Yahr stage 1–5; PD recorded in ON state	GYENNO Science Parkinson's Disease Research Center, Shenzhen, China	No	1
**Italian Torino corpus-1 [28,29]**	/a/, phrases Not reported	Unsupervised, home/web app	Smartphone (web application)	Clinical diagnosis	Polytechnic University of Turin + A.O.U Città della Salute e della Scienza di Torino	No	2

Dataset Name	Voice Task(s) & Duration	Noise Env / Setup	Device & Format	Annotation Method	Source / Institution	Publicly available?	Used?
**Italian Torino corpus-2 [28,29]**	/a/ Not reported	Quiet room (clinic, supervised)	iPhone 12	Clinical diagnosis	Polytechnic University of Turin + A.O.U Città della Salute e della Scienza di Torino	No	2
**Italian Torino corpus-3 [28]**	Same as main dataset: sentences, words, vowels, syllables (/pa/, /ta/), Variable (~5–15 s per sentence)	Home / domestic environments (sub-optimal, uncontrolled)	Non-professional microphones (via web application, unspecified) WAV, 16 kHz	Clinical diagnosis ; exclusion of major cognitive impairment	Politecnico di Torino + A.O.U. Città della Salute e della Scienza di Torino + Associazione Amici Parkinsoniani Piemonte Onlus + Imperia Hospital, Italy	No (restricted, collected for study)	2
**Italian Rome corpus [30]**	Sustained vowel /e/, standardized Italian sentence ≥5 s sustained vowel, ~10–15 s sentence	Quiet, echo-free recording rooms; controlled hospital/lab environments	Voice recordings were collected by using a high-definition audio-recorder H4n Zoom (Zoom connected with a Shure WH20 Dynamic Headset Microphone (Shure Incorporated, USA), which was placed at a distance of 5 cm from the mouth. Corporation, Tokyo, Japan),WAV, 44.1 kHz, 16-bit	Clinical diagnosis by neurologists; includes ON/OFF L-Dopa medication status, UPDRS and H&Y motor scores	Sapienza University of Rome + IRCCS Neuromed Institute	No – available upon reasonable request to corresponding author (privacy restrictions)	3
**Spanish PD Vowel Dataset [31]**	Sustained vowel /a/	Not reported	Not reported WAV, 44.1 kHz	Clinical diagnosis by neurologists (disease duration 1–16 years)	Universidad Nacional de La Matanza (UNLaM) + Hospital Posadas + Hospital Rivadavia, Argentina	Yes (Figshare: https://figshare.com/articles/dataset/Audios_vowel_A_PD/21453867)	1

* The Parkinson's Telemonitoring Dataset from UCI [6], although not used in any studies, was included for completeness as it serves as a companion to the Oxford PD dataset [5].

Table S3. Summary of Methods and Findings from the reviewed studies

Author (Year)	Datasets used	Voice Task	Model Type	Specific Model	Input Data Type ǀFeatures Ext. Method	Training/validation Method	Best Performance Metrics	Limitations
**Adnan et al. (2025) [2]**	PARK Framework Dataset, N=1306 (392 PD)	English pangram "the quick brown fox ..." recited in Home, Clinical Setup, and PD Care Facility recordings	Deep Learning	Transformer-Projection-based Fusion Model (WavLM→ ImageBind)	Raw audio pangram utterances ǀ Wav2Vec 2.0, WavLM, ImageBind embeddings	70/15/15 train/validation/test split + 10-fold cross-validation	Transformer: Accuracy: 85.37%, AUROC: 90.86%, Sensitivity: 81.88%, Specificity: 89.53%, F1: 81.08%	Limitations: dataset imbalance (fewer PD participants), reliance on English pangram only, limited explainability of embeddings, underrepresentation of Non-White groups, performance drop in external validation, higher false negatives
**Alalayah et al. (2023) [32]**	Parkinson's Telemonitoring Dataset (UCI), N=42 (20 PD)	Sustained phonation of vowel /a/	Machine Learning	SVM, KNN, Decision Tree, Random Forest, Naïve Bayes	Acoustic features (e.g., jitter, shimmer, NHR, HNR, RPDE, DFA, PPE) ǀ Extracted from sustained vowel phonation	Leave-One-Subject-Out Cross-Validation (LOSO)	Random Forest: Accuracy: 99% , Precision: 95%, Recall: 98 %, F1: 96%	Limitations: small dataset size (42 subjects), limited to sustained phonation, no external validation, limited demographic diversity
**Ali et al. (2024) [33]**	Two datasets:<br>Oxford PD Speech Dataset (UCI), N=31 (23 PD)<br>Istanbul PD Speech Dataset (UCI), N=252 (188 PD)	Sustained phonation of vowel /a/<br>Sustained phonation of vowel /a/	Machine Learning and Deep Learning	L1-regularized SVM + Deep Neural Network (L1SVM-DNN hybrid)	Acoustic voice features (e.g., jitter, shimmer, NHR, pitch, UPDRS-related features) ǀ Extracted with Praat and MDVP software	Leave-One-Subject-Out (LOSO) cross-validation, 10-fold cross-validation, independent test set	CNN-LSTM:<br>Dataset 1: Accuracy: 100% (LOSO, 10-fold), Sensitivity: 100%, Specificity: 100%<br>Dataset 2: Accuracy: 96.42% (LOSO testing), 97.5% (10-fold), Sensitivity: up to 95%, Specificity 100%	Limitations: missing information on disease severity and ON/OFF state in test set, no analysis by disease duration
**Alshammri et al. (2023) [34]**	Oxford PD Speech Dataset (UCI), N=31 (23 PD)	Sustained phonation of vowel /a/	Machine Learning and Deep Learning	Multi-Layer Perceptron (MLP), Support Vector Machine (SVM)	Input features (22 dysphonia measures): Jitter (%), Shimmer (dB), RAP, PPQ, DDP, APQ3/5, NHR, HNR, RPDE, DFA, D2, spread1/2, PPE.	70/30 train/test split with SMOTE, GridSearchCV for hyperparameter tuning, SelectKBest for feature selection	MLP:<br>Accuracy 98.31%, Recall 98%, Precision 100%, F1 99%<br>SVM:<br>Accuracy 95%, Recall 96%, Precision 98%, F1 97%	Limitations: small dataset size (31 subjects), class imbalance requiring SMOTE, limited to sustained phonation only, no external validation
**Amato et al. (2021) [28]**	Two datasets:<br>Italian Parkinson's Voice and Speech (ItalianPVS), N=50 (28 PD)<br>Italian Torino-3 Corpus, N=44 (26 PD)	including reading of a phonetically balanced text, execution of the syllables /pa/ and /ta/, phonation of the vowels /a/, /e/, /i/, /o/, /u/, reading of a list of phonetically balanced words, and finally reading of a list of phonetically balanced sentences<br>Reading of phonetically balanced Italian sentences (subset with unvoiced consonant–voiced transitions)	Machine Learning	Support Vector Machine (SVM with RBF kernel, optimized C and gamma)	Acoustic features (RASTA-PLP, MFCCs, DFA, spectral moments, intensity ratio, duration ratio, ETS) ǀ Extracted with Praat, statistical summarization per transition region	80/20 subject-independent train/test split with model selection and optimization on training/validation, 10-fold cross-validation repeated over 20 iterations	Experiment 1 (controlled conditions): Accuracy 98% ±1.2, AUC=100 ±0.0, Precision 98%±1.6 , Specificity=97% ±2.4, F1 98% ±1.1<br>Experiment 2 (home conditions): Accuracy 86% ±2.5, AUC=94 ±1.9, Precision 90%±2.9 , Specificity=86% ±3.9, F1 87% ±2.3, stable generalization	Limitations: small dataset sizes, heterogeneity of corpora, manual segmentation required, limited generalizability to larger populations

Author (Year)	Datasets used	Voice Task	Model Type	Specific Model	Input Data Type ∣Features Ext. Method	Training/validation Method	Best Performance Metrics	Limitations
**Bhatt et al. (2023) [35]**	Two datasets: PC-GITA Dataset, N=100 (50 PD) Italian Parkinson's Voice and Speech (ItalianPVS), N=50 (28 PD)	natural and modulated phonation of vowels A subset of sustained phonation of vowel /a/ and /o/ recorded in controlled acoustic conditions	Deep Learning	InceptionResNetV2, ResNet50V2, and VGG-16 (transfer learning with DNNs).	Speech signals transformed via High-Resolution Superlet Transform (SLT) ∣ Features extracted from SLT time–frequency representations	10-fold cross-validation	VGG-16: Dataset 1: Accuracy: 92% , Sensitivity: 92%, Specificity: 91% , F1 93% Dataset 2: Accuracy: 96% , Sensitivity: 94%, Specificity: 100% , F1 96%	limited to sustained phonation, no external validation, no analysis of disease severity
**Celik et al. (2023) [36]**	Two datasets: Oxford PD Speech Dataset (UCI), N=31 (23 PD) Istanbul PD Speech Dataset (UCI), N=252 (188 PD)	Sustained vowel /a/ phonations	Machine Learning and Deep Learning	SkipConNet + Random Forest (proposed hybrid model)	Raw speech signals→ frames (25 ms) ∣ Features extracted via CNN layers (SkipConNet) then classified with RF using multidimensional feature vectors	70/30 train/test split, 100 epochs, batch size 16	Dataset 1: Accuracy: 99.11 % , AUC: 98.77%, Specificity: 98.77% , F1 99% Dataset 2: Accuracy: 98.30 % , AUC: 95.83%, Specificity: 95.83% , F1 99%	Limitations: relatively small datasets, class imbalance, restricted to sustained phonation, no external validation
**Chintalapudi et al. (2022) [37]**	Oxford PD Speech Dataset (UCI), N=31 (23 PD)	Sustained vowel /a/ phonations	Deep Learning	LSTM (best) compared with RNN and MLP	Acoustic biomedical voice features (Fo, Fhi, Flo, jitter, shimmer, RAP, PPQ, APQ, NHR, HNR, RPDE, DFA, PPE, Spread1/2, D2) ∣ Min–max normalization + SMOTE oversampling	67/33 train–test split with 100-epoch training and early stopping (batch size = 16)	Accuracy: 98.97%, Precision: 100%, Recall: 97.95%, F1: 98.96%	Limitations: Small dataset (31 subjects), no external validation, overfitting risk due to oversampling, no comparison with multimodal or clinical variables
**Costantini et al. (2023) [38]**	Italian Rome Corpus, N=426 (160 PD)	Sustained vowel /e/ phonation for 5 s at comfortable volume	Machine Learning and Deep Learning	kNN, SVM, Naïve Bayes (best ML: kNN), Custom CNN (2 conv layers + dense layers)	Acoustic features (jitter, shimmer, HNR, MFCCs, wavelet, formants, tremor features) ∣ Voice Analysis Toolbox, Praat scripts, Parselmouth	10-fold cross-validation with Bayesian hyperparameter optimization, data augmentation for CNN	Mid-Advanced PD vs. HC: KNN = 0.80 ± 0.01 acc; CNN = 0.82 ± 0.07 acc. Early PD vs. HC: SVM = 0.83 ± 0.02 acc; CNN = 0.70 ± 0.06 acc. Mid-Advanced PD vs. Early PD: KNN = 0.85 ± 0.02 acc; CNN = 0.74 ± 0.09 acc. ON vs. OFF L-Dopa: KNN = 0.79 ± 0.01 acc; CNN = 0.53 ± 0.08 acc.	Limitations: dataset limited to sustained phonation only, moderate size (though larger than most prior works), mixed recording devices (smartphone vs professional mic), no external validation, CNN underperformed in ON vs OFF tasks, multiclass accuracy relatively low (~61%)
**Dao et al. (2025) [39]**	Extended ICEBERG Cohort Speech Dataset, N=247 (148 PD)	sustained vowels, DDK (syllable repetition slow/fast), reading (1 min), sentence repetition (10 s), and 1-min free-speech monologue	Deep Learning	wav2vec 2.0, Whisper, SeamlessM4T (foundation models, individually and ensemble-finetuned)	Raw waveform / log-Mel spectrogram / Mel-filter banks ∣ Spectral subtraction denoising + SpecAugment + mean pooling on pretrained layers + fine-tuning with classification head	5-fold subject-independent stratified CV (PD/HC + gender balanced)	Best (ensemble finetuned models): AUC 91.35%, Precision 80.84, Recall 91.22, F1 85.71	Limitations: controlled hospital recordings only, limited gender balance (more males), no real-world/noisy validation, slight calibration bias, no cross-language testing

Author (Year)	Datasets used	Voice Task	Model Type	Specific Model	Input Data Type ∣Features Ext. Method	Training/validation Method	Best Performance Metrics	Limitations
**Escobar-Grisales et al. (2023) [40]**	Extended PC-GITA Dataset, N=165 (80 PD)	Spontaneous 90-second description of daily activities, recorded at 44.1 kHz, 16-bit, downsampled to 8 kHz, PD patients in ON state (≤3 h after medication)	Deep Learning	1D-CNN + LSTM, 2D-CNN (ResNet), Wav2Vec 2.0, CNN for NLP (with W2V, BERT, BETO embeddings), SVM for fusion	Speech audio (time–frequency reps, Wav2Vec embeddings) + Text transliterations (W2V, BERT, BETO embeddings) ∣ STFT-mel spectrograms (128 mel filters, 500 ms windows, 250 ms shift), Wav2Vec2.0 embeddings, Word embeddings (W2V trained on Spanish WikiCorpus, multilingual BERT, BETO Spanish BERT)	Speaker-independent 10-fold cross-validation with grid search (SVM) and Adam optimizer (DL models, 200 epochs, early stopping)	Speech (Wav2Vec2.0): Accuracy 88.5%, F1: 88.3%, Sensitivity: 82.5%, Specificity: 94.0% Language (BETO+CNN): Accuracy 77.9%, F1: 76.9% ∣ Fusion (joint): Accuracy 77.2%, balanced sensitivity/specificity	Limitations: relatively small dataset, inability to fine-tune large pre-trained models (Wav2Vec, BETO), loss of information in multimodal fusion (GMM supervectors, mean pooling), reduced performance in multimodal vs speech-only
**Favaro et al. (2023) [13]**	Six datasets: NLS (American English, N=50),Neurovoz (Castilian Spanish, N=79), PC GITA (Colombian Spanish, N=100), GermanPD (German, N=176), ItalianPVS (Italian, N=78), subset of CzechPD (Czech, N=36)	Spontaneous speech (monologue, picture description), Reading passages, Text-dependent utterances (short sentences)	Machine Learning and Deep Learning	Interpretable models: SVM, KNN, RF, XGBoost, Bagging ∣ Non-interpretable models: PLDA with PCA on embeddings (x-vectors, TRILLsson, Wav2Vec 2.0, HuBERT)	Interpretable features (prosodic, linguistic, cognitive descriptors) ∣ DisVoice, Parselmouth, DigiPsychProsody, ∣ Non-interpretable features ∣ Pre-trained embeddings (x-vectors, TRILLsson, Wav2Vec2.0, HuBERT)	Nested cross-validation (10-fold, stratified inner folds, outer test folds)	Mono-lingual best (ItalianPVS, F1=0.92, AUC=0.98)∣ Multi-lingual mean (F1=85%, AUC=0.88) Cross-lingual mean (F1=79%, AUC=0.91)<br><br>Best performer: TRILLsson model (F1 = 88%, AUC = 0.95) showed highest robustness across languages.	Limitations: small and heterogeneous dataset sizes, differences in medication state across corpora, imbalance in task availability, task variants introducing bias, classifier mismatch between interpretable and non-interpretable features, lack of external validation
**Gimeno-Gómez et al. (2025) [41]**	Five Datasets: NeuroVoz (Castilian Spanish, N=79), PC GITA (Colombian Spanish, N=100), FraLusoPark (French and European Portuguese, N=74), GermanPD (German, N=176), CzechPD (Czech, N=100)	Speech tasks: sustained vowels, DDK syllable repetition, isolated words, phonetically balanced sentences, reading passages, and spontaneous monologues	Deep Learning	Interpretable Cross-Attention Framework (using Wav2Vec2.0 XLS-R 300M embeddings + 35 clinically informed features)	Raw audio (resampled 16 kHz) + SSL embeddings ∣ Wav2Vec2.0 XLS-R (7th layer, 1024-dim, 20 ms stride) + DisVoice informed features (35 dimensions: articulation, glottal, phonation, prosody)	Nested cross-validation (5 outer folds, inner folds for validation, repeated 5 times with random seeds, speaker-independent)	Average F1 ≈ 74–79 % for SSL baseline, ≈ 73 % for proposed Cross-Attention (competitive accuracy with added interpretability).<br><br>Performance per task (F1 %): Vowels 62.1±4.8 ; Words 72.9±2.9; DDK 72.7±7.1; Sentences 78.2±5.3; Read-text 74.5±2.9; Monologue 73.1±3.9.	Limitations: interpretability not medically validated, occasional performance drops vs SSL-only, task/dataset variability, reliance on attention scores which may not directly reflect feature severity
**Hadjaidji et al. (2025) [42]**	Oxford PD Speech Dataset (UCI), N=31 (23 PD)	Sustained phonation vowel	Machine Learning	KNN (best), also SVM, Gradient Boosting, Naïve Bayes	Acoustic features (23 voice measures: jitter, shimmer, NHR, HNR, RPDE, DFA, PPE, etc.) ∣ Pre-computed MDVP-based features from OPD dataset	5-fold stratified cross-validation with SMOTE balancing and z-score normalization	Best: KNN (k=1) Accuracy: 97.44%, Sensitivity: 98.02%, F1: 0.974, MCC: 0.933, AUC: 0.972	Limitations: very small dataset size, lack of raw recordings (only pre-computed features), high computation time when many features used, no external validation

Author (Year)	Datasets used	Voice Task	Model Type	Specific Model	Input Data Type ǀFeatures Ext. Method	Training/validation Method	Best Performance Metrics	Limitations
**Hawi et al. (2022) [43]**	Istanbul PD Speech Dataset (UCI), N=252 (188 PD)	Sustained phonation of vowel /a/	Machine Learning	Random Forest	Acoustic features (long-term: jitter, shimmer, formants, intensity, DFA ǀ short-term: MFCCs 1–13) ǀ Extracted with Praat-based tools	75/25 train/test split with 5-fold cross-validation	Accuracy: 88.84%, Sensitivity: 98.51%, Specificity: 71.08%	Limitations: imbalanced dataset (75% PD vs 25% HC), low specificity due to bias toward PD class, lack of early-stage PD cases, no external validation, gender differences not modeled
**He et al. (2024) [44]**	Two datasets:<br>MDVR-KCL Dataset, N=37 (16 PD)<br>mPower Voice Dataset (subsample: N=60, 30 PD)	Read-text ("North Wind and the Sun", technical passage) and spontaneous dialog via smartphone calls<br><br>Sustained vowel /a/ samples via Smartphone-based reading, dialog, and sustained vowel /a/ phonation, 10 s per task-1500	Machine Learning	Gradient Boosting (GB), Support Vector Machine (SVM with RBF), Logistic Regression (LR)	Smartphone audio fragments (10 s) ǀ openSMILE IS09 (384 LLDs + functionals) + GeMAPS (62 LLDs), 446 features total	70/30 train/test split, grid search with 5-fold cross-validation for hyperparameters	MDVR-KCL spontaneous dialog: GB-Var Accuracy 97.1%, AUC 0.989, F1 0.968<br>MDVR-KCL read text: SVM-RFECV Accuracy 100%, AUC 1.0, F1 1.0<br>MDVR-KCL combined: SVM-Ken Accuracy 98.4%, AUC 0.996, F1 0.979<br>mPower subset: SVM-RFECV Accuracy 90.4%, AUC 0.959, F1 0.902	Limitations: smartphone variability, environmental noise, imbalance in mPower dataset, limited sample sizes in MDVR-KCL, no deep learning due to data scarcity, cross-database generalization not feasible
**Hireš et al. (2022) [45]**	PC-GITA dataset, N=100 (50 PD)	Sustained vowel phonations /a/, /e/, /i/, /o/, /u/	Deep Learning	CNN ensemble (ResNet50 and Xception backbones with Multiple Fine-Tuning and ensemble voting)	Raw voice recordings ǀ STFT spectrograms (40 ms window, log-spectra, Gaussian blurring applied)	10-fold cross-validation (subject-independent)	Best results (PC-GITA vowels):<br>/a/ Accuracy 99 ± 2.13%, AUC 89.8<br>/e/ Accuracy 96.67 ± 3.33%, AUC 89.9<br>/i/ Accuracy 92 ± 7.78%, AUC 83.1<br>/o/ Accuracy 92 ± 6.18%, AUC 83.8<br>/u/ Accuracy 91.33 ± 7.02%, AUC 88.6	Limitations: no medication state info, limited to sustained vowels, language- and corpus-specific, no external validation
**Hireš et al. (2023) [46]**	Four datasets:<br>CzechPD (Czech, N=32), PC-GITA (Spanish, N=100), ItalianIPVS (Italian, N=50), subsample of RMIT-Smartphone Phoneme Dataset (N=41)	Sustained vowel /a/ phonation across datasets	Machine Learning and Deep Learning	XGBoost (shallow ML) and Xception CNN (deep learning)	Acoustic features (formants, jitter, shimmer, MFCCs, spectral, prosodic, PPE, DFA, etc.) ǀ Handcrafted features (Praat-based + statistical measures)<br>CNN input: log-frequency power spectrograms (via STFT)	10-fold cross-validation (speaker-independent), cross-dataset training/testing	Within-dataset: ITA (CNN Accuracy 97.8%, AUC 94.9%), RMIT-PD (CNN Accuracy 94.8%, AUC 94.99%), PC-GITA (CNN Accuracy 90.5%, AUC 89.09%), CzechPD (CNN Accuracy 90.8%, AUC 90.79%) , Cross-dataset: accuracy dropped to 33–74% depending on dataset pair	Limitations: limited to sustained vowels, small dataset sizes, lack of gender balance (CzechPD only males), no dysarthria from other disorders considered, poor cross-dataset generalization, recording device/environment variability
**Hoq et al. (2021) [47]**	Istanbul PD Speech Dataset (UCI), N=252 (188 PD)	Sustained phonation of vowel /a/	Machine Learning and Deep Learning	PCA-SVM and SAE-SVM (Sparse Autoencoder + Support Vector Machine, polynomial kernel)	Acoustic features (baseline: jitter, shimmer, HNR, PPE, DFA, RPDE MFCCs, wavelet, TQWT, vocal fold measures) ǀ Extracted with Praat, MFCC pipeline, Wavelet, TQWT	70/30 train/test split, 10-fold cross-validation, SMOTE balancing	SAE-SVM: Accuracy 93.5%, F1-score 0.951, MCC 0.788<br>PCA-SVM: Accuracy 88.9%, F1-score 0.928, MCC 0.70	Limitations: dataset imbalance (188 PD vs 64 HC), limited to sustained vowels only, no external validation, feature-engineered dataset may not generalize

Author (Year)	Datasets used	Voice Task	Model Type	Specific Model	Input Data Type |Features Ext. Method	Training/validation Method	Best Performance Metrics	Limitations
**Hossain et al. (2023) [48]**	Istanbul PD Speech Dataset (UCI), N=252 (188 PD)	Sustained phonation of vowel /a/	Machine Learning	Best model: AdaBoost with LSVC pipeline	Acoustic features (baseline jitter, shimmer, RPDE, DFA, PPE, MFCCs, formants, wavelet, TQWT, vocal fold features) | Extracted with Praat, Wavelet, TQWT, MFCC pipelines	70/30 patient-level train/test split with 10-fold cross-validation, SMOTE applied, LSVC for feature selection in pipelines	Best supervised model: AdaBoost Accuracy 84.21%, Precision 0.91, Sensitivity 0.87, F1: 0.89, AUC 0.87 Best pipeline (AdaBoost+LSVC): Accuracy 85.09%, Precision 0.92, Sensitivity 0.90, F1: 0.91, AUC 0.90	Limitations: dataset lacks disease stage/treatment/duration info, limited to sustained vowels only, no external validation
**Ibarra et al. (2023) [49]**	Four Dataset: PC-GITA (Spanish, N=100), Neurovoz Dataset (Spanish, N=91), PD-German (German, N=176), PD-Czech (Czech, N=100)	Sustained vowel /a/ phonation and diadochokinetic (DDK) syllable repetition (/pa-ta-ka/)	Deep Learning	2D-CNN, Time-CNN-LSTM, 1D-CNN with and without Domain Adversarial (DA) training	Speech audio | Mel spectrograms (65 mel bands, 40 ms windows for vowels, 15 ms frames for DDK, 10 ms hop, z-score normalization)	Stratified speaker-independent 10-fold cross-validation with transfer learning from Saarbrücken Voice Disorders Database	Baseline within-corpus: Acc 64–80%, F1 up to 80%, Sen. up to 92% Cross-corpus baseline: Acc often <65% With DA: higher intra-class cohesion, reduced domain divergence, accuracies up to 82.6% (DDK, PD-Neurovoz, 1D-CNN DA)	Limitations: relatively small corpora, variability in language, recording equipment, and environments, sustained vowels less informative, DA models still rely on non-interpretable DL
**Iyer et al. (2023) [50]**	Telephone PD Voice Dataset (UAMS), N=81 (40 PD)	Sustained vowel /a/ (~3 s) recorded via telephone line (8 kHz, 16-bit), natural home settings	Machine Learning and Deep Learning	Inception V3 CNN with transfer learning (best) vs. Random Forest, Logistic Regression	Raw audio | CNN: spectrogram images (32 ms window, 50% overlap, FFT=1024, 600×600 px color/grayscale) ML: 23 phonatory features (F0, formants, jitter, shimmer, HNR) + LPC, LAR, Cepstral, MFCC features via Parselmouth, R packages	70/30 train/test split repeated 100 times, 3-fold CV for ML classifiers	CNN: AUC 0.97 (color), 0.96 (grayscale) RF: AUC up to 0.73 LR: AUC up to 0.66	Limitations: CNN lacks interpretability, HC group only self-reported health status (not neurologist confirmed), telephone audio low resolution (8 kHz), small dataset size, no external validation
**Jeancolas et al. (2021) [19]**	ICEBERG Cohort (French), N=221 (121 PD)	reading (1 min), sentence repetition (10–20 s), free speech monologue (1 min), diadochokinetic (DDK) fast syllable repetition (1–1.5 min)	Deep Learning	X-vector embeddings from Time Delay Neural Network (TDNN) with cosine similarity, LDA, PLDA classifiers (compared against MFCC-GMM baseline)	Input: MFCCs (23 or 30 + log energy) | Extraction with Kaldi, VAD, cepstral mean subtraction, DNN pretrained on VoxCeleb (16 kHz) or SRE16 (8 kHz) for x-vectors	repeated random subsampling with ensemble aggregation (40 runs, balanced male/female PD vs HC groups), segment duration matched (1–5 s)	Best performance: Early PD, females, free speech, high-quality mic, x-vector+PLDA with data augmentation, EER=30% (15% improvement vs MFCC-GMM)	Limitations: limited to cepstral features, no OFF-state recordings (all PD ON-medication), gender differences, text-dependent tasks less suited for x-vectors, lack of interpretability of DNN embeddings, non-public dataset

Author (Year)	Datasets used	Voice Task	Model Type	Specific Model	Input Data Type ∣Features Ext. Method	Training/validation Method	Best Performance Metrics	Limitations
**Jeong et al. (2024) [12]**	Korean PD Speech Dataset (subsample), N=200 (100 PD)	Speech tasks: sustained vowels (/a/, /e/, /i/, /o/, /u/ repeated twice), consonant repetitions (/ga/, /na/, /da/, /ha/ ×3), diadochokinetic (DDK) /pa-ta-ka/ for 10s	Machine Learning and Deep Learning	Voting-based ensemble (CatBoost, LightGBM, XGBoost, ExtraTrees, Random Forest) and compared with Transformer Audio Spectrogram Transformer (AST, Transformer-based DL model).	Acoustic features (89 total: 88 eGeMAPSv2 + gender) ∣ Extracted with openSMILE (eGeMAPSv2) ∣ Data segmented into 5-s windows (1-s overlap), z-score normalization	10-fold cross-validation and held-out test set	Voting ML Model: Accuracy = 84.73 %, F1 = 85.08 %, AUC = 92.18 %, Specificity = 84.22 %, EER = 15.27 %.<br><br>AST Model: Accuracy = 79.30 %, AUC = 87.30 %.	Limitations: dataset limited to Korean only, not publicly available, small sample size, AST (transformer baseline) underperformed due to data scarcity, gender differences influence classification, no external validation
**Karabayir et al. (2020) [51]**	Parkinson Dataset with Replicated Acoustic Features (UCI), N=80 (40 PD)	Sustained phonation of vowel /a/ for 5 s, repeated three times	Machine Learning	Light Gradient Boosting (best), also Extreme Gradient Boosting, RF, SVM, KNN, LASSO, LR	Acoustic features (44 baseline + replicated across 3 runs: jitter, shimmer, HNR, MFCCs, delta coefficients, RPDE, DFA, PPE) ∣ Extracted via VOICESAUCE and Praat-based pipelines	4-fold cross-validation repeated 100 times, also 5- and 10-fold CV for sensitivity analysis	Best: LightGBM Accuracy 0.841 [0.833–0.849], F1=0.839 [0.831–0.847], Sensitivity 0.839 [0.827–0.850], Specificity 0.844 [0.832–0.855], AUC=0.898 [0.892–0.905]	Limitations: small sample size, all PD subjects from single study, early untreated PD only, no external validation, no comparison with other neurological disorders
**Karaman et al. (2021) [52]**	mPower Voice Dataset, subsample of 33,877 audio files (10,589 PD)	Sustained phonation of vowel /a/ ("aaaah") for up to 10 seconds at steady pitch and loudness.	Deep Learning	Transfer Learning CNNs (DenseNet-161 best, also ResNet-50, SqueezeNet1_1)	Raw audio ∣ Preprocessing: DCT (type II) + 320 Mel bins → Mel-spectrograms (448×448 px PNG, log-scaled colormap) ∣ Classified via CNN fine-tuning	Training: 18,660 HC + 8442 PD, Validation: 4628 HC + 2147 PD, Independent Test: 200 HC + 200 PD	Best (DenseNet-161): Validation: Accuracy = 91.17%, Sensitivity = 84.47%, Precision = 89.25%.<br>Testing: Accuracy = 89.75%, Sensitivity = 91.50%, Precision = 88.40%.	Limitations: potential bias from self-reported diagnosis, no medication state consistency, excluded DBS patients, lack of external validation, sustained vowels only
**Karapinar Senturk (2020) [53]**	Oxford PD Speech Dataset (UCI), N=31 (23 PD)	Sustained vowel /a/ phonations	Machine Learning	Support Vector Machine (SVM with Recursive Feature Elimination, best), also CART and ANN	Acoustic features (23 voice measures: jitter, shimmer, HNR, NHR, RPDE, DFA, PPE, etc.) ∣ Precomputed features from sustained vowels, selected via RFE and Feature Importance	10-fold cross-validation, grid search for hyperparameters, ANN trained with 150 epochs, batch size 50	SVM+RFE: Accuracy 93.84%,<br>ANN+RFE: Accuracy 91.54%,<br>CART+FI: Accuracy 90.76%	Limitations: very small dataset (31 subjects), no external validation, only sustained vowels used, limited demographic information
**Kiran Reddy et al. (2025) [54]**	PC-GITA Dataset, N=100 (50 PD)	Speech tasks: (i) diadochokinetic (DDK) syllable repetitions (/pa-ta-ka/, /pe-ta-ka/, etc.), (ii) spontaneous monologue (daily routine), (iii) text reading (doctor–patient dialogue), (iv) sentence reading (6 sentences)	Machine Learning and Deep Learning	Feed-Forward Neural Network (FFNN, best) and Support Vector Machine (SVM with RBF kernel)	Raw audio ∣ Two-layer Wavelet Scattering Network features (164 coefficients per frame) → Fisher Vector encoding (656-dim) → Feature Selection via Neighborhood Component Analysis (reduced to 64 features)	10-fold speaker-independent cross-validation with Bayesian Hyperparameter Optimization	Best :Text Reading, FFNN: Accuracy 87.0%, MCC 0.76, AUC 0.95<br>Monologue (FFNN): Accuracy 85.0%, AUC 0.93<br>DDK (FFNN): Accuracy 80.0%, AUC 0.88	Limitations: single-language dataset, not public, performance lower in fusion of tasks, no cross-database validation, generalization to accents/emotions untested

Author (Year)	Datasets used	Voice Task	Model Type	Specific Model	Input Data Type ǀFeatures Ext. Method	Training/validation Method	Best Performance Metrics	Limitations
**Klempíř et al. (2023) [17]**	Three datasets: Czech PD-DDK /pa/ dataset, N=60 (30 PD, male speakers) Italian Parkinson's Voice and Speech (ItalianPVS) N=50 (28 PD, 22 HC +15 young HC), MDVR-KCL English dataset, N=37 (16 PD)	Tasks: rhythmic syllable repetition (/pa/, Czech), text reading (Italian, English)	Machine Learning and Deep Learning	Wav2Vec embeddings + Random Forest classifier (ensemble)	Raw audio (resampled 16 kHz) ǀ Wav2Vec-large pre-trained embeddings (512-dim, mean and sum pooling)	5-fold cross-validation with 5 repeats, leave-group-out (Italian vs. English)	Dataset-1 (/pa/): AUROC 0.81, Accuracy 0.71, Precision 0.73, Recall 0.73 Dataset-2 (Italian reading): AUROC 0.98, Accuracy 0.95, Precision 0.94, Recall 0.97 Dataset-3 (English reading): leave-group-out AUROC 0.81, Accuracy 0.68, Precision 0.63, Recall 0.93)	Limitations: demographic imbalance (male-only Czech dataset, Italian dataset skewed male), small datasets, lack of hyperparameter tuning, wav2vec embeddings not easily interpretable, varied recording devices/environments
**Klempíř et al. (2024) [55]**	Three datasets: Czech PD-DDK /pa/ dataset, N=60 (30 PD, only male speakers) Italian Parkinson's Voice and Speech (ItalianPVS), N=50 (28 PD, 22 HC +15 young HC) MDVR-KCL English dataset, N=37 (16 PD)	rhythmic syllable repetition (/pa/, Czech), text reading (Italian, English)	Machine Learning and Deep Learning	Wav2Vec 1.0 embeddings + Random Forest, Logistic Regression, XGBoost, Lasso Regression	Raw audio (16 kHz resampled) ǀ Wav2Vec 1.0 embeddings (512-dim, mean/std/sum pooling, PCA 10 comps) + MFCCs (50 coefficients via Librosa, PCA 10 comps)	5-fold cross-validation with 5 repeated fits	Intra-dataset: Italian AUROC=0.98–0.99 (wav2vec-mean and MFCC mean), English AUROC=0.80 (wav2vec-std), Czech /pa/ AUROC=0.84 (MFCC-PCA)<br><br>Cross-database: English→Italian AUROC=0.90 (RF), 0.98 (XGBoost) Italian→English AUROC=0.72–0.78 Czech→Italian/English AUROC≈0.46–0.68 Regression (Italian): age R=0.56, articulation rate R=0.74, loud region duration R=0.84	Limitations: focused only on wav2vec 1.0 (not 2.0), limited datasets, weak performance for simple /pa/ tasks, gender/age imbalance in Italian dataset, regression age gaps
**Klempir et al. (2025) [56]**	Three datasets: MDVR-KCL English dataset, N=37 (16 PD) PC-GITA dataset, Spanish, N=100 (50 PD) Telephone PD Voice Dataset (UAMS), English, N=81 (40 PD)	Voice tasks: rhythmic syllable repetition (/pa/, Czech), text reading (Italian, English), spontaneous dialogue (English), recorded at 44.1–48 kHz, resampled to 16 kHz	Machine Learning	Random Forest, SVM, DT, KNN, LR (best varied by dataset/task)	Wav2Vec 1.0 and Wav2Vec 2.0 embeddings (FE, 1T, LH layers) ǀ Aggregated embeddings (sum/mean), PCA-reduced to 30 components	5-fold cross-validation with 5 repeats, leave-group-out for cross-language evaluation	English read text (W2V2-1T+SVM): Accuracy 86%, AUC 0.89 English dialogue (W2V1-FE+DT): Accuracy 90%, AUC 0.91 Spanish PC-GITA monologue (W2V2-1T+SVM): Accuracy 85%, AUC 0.91 PC-GITA vowels (W2V2-FE+RF): Accuracy 75%, AUC 0.85 Fusion vowels (W2V1+W2V2): Accuracy 80%, AUC 0.86	Limitations: small dataset sizes, demographic imbalance (Czech male-only, Italian skewed male), wav2vec embeddings not interpretable, varied recording conditions, no hyperparameter tuning, limited external validation

Author (Year)	Datasets used	Voice Task	Model Type	Specific Model	Input Data Type ǀFeatures Ext. Method	Training/validation Method	Best Performance Metrics	Limitations
**Kumar et al. (2025) [57]**	Istanbul PD Speech Dataset (UCI), N=252 (188 PD)	Sustained phonation of vowel /a/	Machine Learning	Best models: KNN, AdaBoost, ANN	Acoustic features (jitter, shimmer, F0, HNR, RPDE, DFA, PPE, MFCCs, WT coefficients, glottal/vocal fold measures) ǀ Extracted from sustained vowels using Praat and wavelet transform	Multiple train/validation/test splits with resampling and normalization, evaluated with 5 experimental trials, average and maximum metrics reported	KNN: Accuracy 98.52%, Avg. Accuracy 97.33%, AUROC 0.973, Precision 0.98, Recall 0.98, F1 0.97 AdaBoost: Accuracy 94.08%, Avg. Accuracy 91.56%, AUROC 0.9752, Precision 0.94, Recall 0.89, F1 0.91 ANN: Accuracy 95.39%, Avg. Accuracy 93.16%	Limitations: dataset imbalance (75% PD vs 25% HC), small dataset size, limited to sustained vowels, no external validation, need for hyperparameter optimization
**Kumari et al. (2025) [58]**	MDVR-KCL Dataset, N=37 (16 PD)	read text passages and spontaneous dialogues recorded via smartphone sliced into 5–8 s segments	Deep Learning	Deep Convolutional Neural Network (DCNN, tapering hidden layers)	Speech spectrum images ǀ LSF spectrums (for detection), STFT spectrums (for severity grading), MFCC spectrums (comparison)	Speaker-dependent 80/20 train/test split, additional speaker-independent experiments	PD detection (binary, LSF): Validation Accuracy 87.5% PD severity grading (multiclass, STFT): Validation Accuracy 93.75%	Limitations: relatively small dataset size, speaker-dependent setup may inflate performance, reduced accuracy in speaker-independent setting, lack of external validation, only English language
**Lim et al. (2024) [11]**	Two datasets: Korean Speech Dataset, N=291 (161 PD), 2068 audio files Taiwanese Speech Dataset, N=360 (186 PD), 360 audio files	Korean: sustained vowels, syllable repetition, sentence repetition, reading tasks (short ≤40 chars, long >40 chars) Taiwanese: standardized fixed 500-character article reading all recordings with smartphones in clinic	Machine Learning	Random Forest, AdaBoost, SVM (best: Random Forest, AdaBoost)	Acoustic + linguistic speech features (volume variance, pitch variance, average pitch, pause %, speech rate, WER, API confidence) ǀ Extracted using Python pysptk + Google Speech-to-Text API	Leave-One-Out Cross-Validation (LOOCV)	Advanced PD vs HC (validation, RF): AUROC 0.90 Korean-only: AUROC 0.87 Taiwanese-only: AUROC 0.88 Short-speech merged: AUROC 0.72 (early PD), AUROC 0.56 (advanced PD)	Limitations: imbalance in languages, lack of dialectal variation, sex imbalance (more females in HC), no OFF-medication data for Koreans, excluded qualitative features (e.g., timbre), limited generalizability
**Meral et al. (2025) [59]**	Istanbul PD Speech Dataset (UCI), N=252 (188 PD)	Sustained phonation of vowel /a/	Machine Learning	Stacking ensemble with DT, SVM, k-NN, NN, and Ensemble as base learners	Acoustic features (Baseline: Jitter, Shimmer, Fundamental frequency, Harmonicity, RPDE, DFA, PPE Time-frequency: Formants, Bandwidth MFCCs: 84 features Wavelet transform: 182 features Vocal fold: GQ, GNE, VFER, EMD) ǀ Chi-Square feature selection (threshold >20) with 156 features retained from original 753	5-fold cross-validation with 70/30 train-test split and stratified sampling	Accuracy: 92.07%, Precision: 96.69%, Recall: 93.58%, F1: 95.11%, AUC: 0.95	Not reported

Author (Year)	Datasets used	Voice Task	Model Type	Specific Model	Input Data Type ǀFeatures Ext. Method	Training/validation Method	Best Performance Metrics	Limitations
**Mohammadi et al. (2021) [60]**	Istanbul PD Speech Dataset (UCI), N=252 (188 PD)	Sustained phonation of vowel /a/	Machine Learning	Logistic Regression stacking ensemble (SVM with 23-degree polynomial kernel, XGBoost, MLP, Autoencoder+SVM)	Acoustic features (Baseline: 21 features, Time-frequency: 11 features, MFCCs: 84 features, Vocal fold: 22 features, TQWT: 615 features) ǀ Min-max normalization with autoencoder feature extraction for dimensionality reduction	5-fold cross-validation with subject-level voting strategy	Accuracy: 97.22%, F1: 98.16%	Low number of samples compared to features, imbalanced dataset with fewer early-stage PD patients, 7-10% prediction error in detecting non-PD patients remains
**Mohapatra et al. (2025) [61]**	Istanbul PD Speech Dataset (UCI), N=252 (188 PD)	Sustained phonation of vowel /a/	Machine Learning	Grid Search Optimized CatBoost (GSO-CatBoost)	Multi-feature subsets (Baseline: 21 features, MFCCs: 84 features, Time-frequency: 11 features, WT: 182 features, Vocal fold: 22 features, TQWT: 432 features) ǀ Min-max normalization with RReliefF feature selection for dimensionality reduction	80/20 train-test split with 5-fold cross-validation and SMOTE oversampling for class imbalance	Accuracy: 92.61% (50 features),96.33%, Sensitivity: 93.75%, F1-score: 95.02%, Specificity: 89.47%, AUC: 95.49% Accuracy: 93.33% (100 features), Precision: 96.33%, Sensitivity: 94.64%, F1-score: 95.54%, Specificity: 89.47%, AUC: 97.68%	Small dataset size limiting generalizability, imbalanced dataset with fewer early-stage PD patients, model evaluated on clean voice recordings without considering real-world noise conditions or recording quality variations
**Momeni et al. (2025) [62]**	Three datasets:<br>mPower dataset, N=5,826 (968 PD, >65k recordings)<br>Lithuanian (Vaiciukynas) PD Speech Datase, N=99 (64 PD),<br>Telephone PD Voice Dataset (UAMS) , N=81 (40 PD)	Sustained phonation of vowel /a/ recorded via mobile phone (10 seconds for mPower, 5 seconds for Vaiciukynas, 3 seconds for UAM)	Machine Learning	XGBoost with RFECV feature selection	eGeMAPS features (88 features), emobase (988 features), ComParE (6373 features), KTU (1267 features) ǀ OpenSMILE framework for feature extraction with group-wise RobustScaler normalization	10-fold cross-validation with GroupShuffleSplit (subject-independent) and 20% test split for unseen individuals	Accuracy: 82% (mPower ComParE), 81.6% (best unseen data), AUC: 90%, F1: 80.0%	Age and gender imbalances between PD and HC groups, uncontrolled recording environments in mPower dataset leading to poor quality recordings, limited to sustained phonation tasks not representative of everyday speech
**Motin et al. (2022) [63]**	RMIT-Smartphone Phoneme Dataset, N=72 (36 PD)	Sustained phonemes /a/, /o/, /m/ recorded using iOS smartphone (iPhone 6S Plus) held in typical phone call position for comfortable duration in real-world clinical setting with ambient noise (16-24 dB SNR)	Machine Learning	SVM with RBF kernel	Acoustic features (Jitter: 6 parameters, Shimmer: 6 parameters, TKEO, HNR, NHR, pitch statistics, GQ, GNE, VFER, MFCCs: 22 features, DWT: energy/entropy coefficients) ǀ Relief-F feature selection (top 15 features) with Mann-Whitney U statistical testing	Leave-one-out cross-validation with stratified sampling	Accuracy: 100%, Sensitivity: 100%, Specificity: 100%, F1: 100% (combination /a/+/o/+/m/)	Participants from single geographic region (suburban Melbourne) may limit accent generalizability, single smartphone device used limiting device variability testing, PD participants >2 years post-diagnosis not representing very early stage disease

Author (Year)	Datasets used	Voice Task	Model Type	Specific Model	Input Data Type ǀFeatures Ext. Method	Training/validation Method	Best Performance Metrics	Limitations
**Naeem et al. (2025) [64]**	Oxford PD Speech Dataset (UCI), N=31 (23 PD)	Sustained phonation of vowel /a/	Machine Learning	Random Forest (RF), Support Vector Machine (SVM), Logistic Regression (LR), Decision Tree (DT)	Vocal features (MDVP: F0, Fhi, Flo, Jitter parameters, Shimmer parameters, NHR, HNR, RPDE, D2, DFA, Spread1, Spread2, PPE) ǀ Standard Scaler normalization with SMOTE for class imbalance and PCA for feature selection	80/20 train-test split with 5-fold cross-validation	RF: Accuracy: 94%, Precision: 94%, F1: 96% SVM: Accuracy: 92%, Precision: 91% F1: 95%	Small dataset size (N=31) limiting generalizability, imbalanced classes with fewer early-stage PD patients, potential issues with background noise and recording quality variations in real-world applications
**Narendra et al. (2021) [65]**	PC-GITA Dataset, N=100 (50 PD)	Continuous speech tasks (DDK exercises, reading words/sentences aloud, monologue) downsampled to 16 kHz	Deep Learning	CNN+MLP end-to-end system with traditional SVM pipeline comparison	Voice source waveforms (IAIF, QCP glottal inverse filtering, ZFF) + baseline acoustic features (articulation, phonation, prosody) ǀ Glottal flow estimation using IAIF (q=10, p=24) and QCP analysis with 30ms frames, 15ms shift	10-fold cross-validation with speaker-independent splits (90% training, 10% validation per fold)	Traditional pipeline: 67.93% (baseline+QCP glottal features) End-to-end: 68.56% (QCP-based glottal flow signals)	Modest classification accuracies for all systems, limited to Spanish language recordings, small dataset size constraining deep learning performance, glottal inverse filtering methods may introduce estimation errors
**Nijhawan et al. (2023) [66]**	Istanbul PD Speech Dataset (UCI), N=252 (188 PD)	Sustained phonation of vowel /a/ recorded three times following physician's examination using microphone at 44.1 kHz	Deep Learning	Vocal Tab Transformer (modified transformer with feature embedding and MLP head)	Dysphonia measures (753 features: Baseline, MFCCs, TQWT, Vocal fold, Wavelet transform features) ǀ XGBoost-based feature selection (top 96 features) with standardization and ADASYN oversampling	10-fold cross-validation with stratified splits ensuring subject independence	Vocal Tab Transformer: AUC = 0.914 ± 0.0037, Precision = 0.882, Recall = 0.904.<br><br>XGBoost: AUC = 0.903; MLP = 0.873; RF = 0.894.	Limited to smaller datasets constraining deep learning performance, imbalanced dataset requiring oversampling techniques, transformer architecture not widely explored for tabular vocal data
**Noaman Kadhim et al. (2024) [67]**	Oxford PD Speech Dataset (UCI), N=31 (23 PD)	Voice recordings with 23 extracted vocal features from sustained phonation	Machine Learning	Gower distance-based classifier with Cuckoo Search optimization outperform MLP and RF	Acoustic features (23 features: Amplitude measures, Jitter variations, Shimmer variations, Frequency measures, Nonlinear measures, HNR, NHR, DFA, RPDE) ǀ Maximum absolute value normalization with Cuckoo Search feature selection	70/30 train-test split	Accuracy: 98.3% (with feature selection), 94.92% (without feature selection), Precision: 99.14%, Recall: 91.76%, F1: 95.28%	Not reported

Author (Year)	Datasets used	Voice Task	Model Type	Specific Model	Input Data Type ∣Features Ext. Method	Training/validation Method	Best Performance Metrics	Limitations
**Oliveira et al. (2025) [68]**	PC-GITA Dataset, N=100 (50 PD)	Tasks: six diadochokinetic (DDK) tasks (/pa/, /ta/, /ka/, /pa-ta-ka/, /pa-ka-ta/, /pe-ta-ka/) categorized into 4 class	Machine Learning	Ensemble (stacked) model combining Logistic Regression, Random Forest, and Gradient Boosting	Acoustic features (phonation, articulation, prosody, and fusion) ∣ DisVoice toolkit (jitter, shimmer, F0 contour, MFCCs onset/offset, Bark band energies, formants, energy, voiced/unvoiced durations)	Leave-one-out cross-validation, stacked ensemble for 4-class (Normal–Slight–Mild–Moderate)	Normal vs Not Normal (LR, "ka-ka-ka", Prosody): Accuracy = 0.88 ± 0.09, Sensitivity = 0.86, Specificity = 0.88, MCC = 0.62.<br><br>Slight vs Not Slight (RF, "pe-ta-ka", Articulation): Accuracy = 0.78 ± 0.12, Sensitivity = 0.74, Specificity = 0.81, MCC = 0.56.<br><br>Mild vs Not Mild (RF, "ka-ka-ka", Fusion): Accuracy = 0.72 ± 0.12, Sensitivity = 0.75, Specificity = 0.71, MCC = 0.43.<br><br>Moderate vs Not Moderate (GB, "ta-ta-ta", Prosody/Fusion): Accuracy = 1.00 ± 0.00, Sensitivity = 1.00, Specificity = 1.00, MCC = 1.00.<br><br>Stacked Multiclass Ensemble: Overall Accuracy = 72 ± 0.12.	Limitations: Small and imbalanced dataset, single-center Spanish corpus, no external validation, no longitudinal tracking, potential overfitting (MCC=1.0 for small Moderate class)
**Pah et al. (2023) [69]**	PC-GITA + SVD datasets, N=200 (50 PD from PC-GITA, 50 HC, and 50 dysphonia, 50 laryngitis from SVD)	Sustained vowels (/a/, /i/, /u/) recorded in noise-controlled soundproof booth	Machine Learning	Support Vector Machine with Gaussian kernel	Voice features (54 features: 18 features from each vowel including jitter, shimmer, pitch SD, harmonics, voice intensity, formants F1-F4, MFCCs 1-5) ∣ Relief-F feature selection (k=10) with mean/SD normalization	Leave-one-subject-out cross-validation	Binary SVM (PD vs non-PD): Accuracy: 77.46%, Recall: 94.00%, Specificity: 68.48%, F1: 74.60%	Models trained only on PD and healthy subjects may misclassify other voice pathologies, limited to binary classification performance, cross-dataset compatibility concerns between PC-GITA and SVD
**Pandey and Sahu (2025) [70]**	Three datasets:<br>Italian Parkinson's Voice and Speech (ItalianPVS), N=50 (28 PD)<br>Spanish PD Vowel Dataset, N=119 (55 PD)<br>Telephone PD Voice Dataset (UAMS) , N=81 (40 PD)	Sustained vowels /a/, /e/, /i/, /o/, /u/ (Italian), /a/ (US-English and Spanish)	Deep Learning	Hybrid Siamese Neural Network with Support Vector Machine (SNN-SVM)	Hybrid MFCC + Time-frequency features ∣ MFCC (13 coefficients, 25ms Hamming window) + Time-frequency features using STFT with Non-negative Matrix Factorization	80/20 train-test split with leave-one-speaker-out cross-validation ensuring speaker independence	Accuracy: 93% (Italian /o/),<br>82.76% (US-English /a/),<br>83.48% (Spanish /a/),<br>Cross-lingual: 82.7% (Spanish→US-English)	Limited to specific vowel tasks not representing full speech patterns, different acoustic environments affecting cross-language performance, single device recording limiting device variability testing

Author (Year)	Datasets used	Voice Task	Model Type	Specific Model	Input Data Type ∣Features Ext. Method	Training/validation Method	Best Performance Metrics	Limitations
**Qasim et al. (2021)[71]**	Istanbul PD Speech Dataset (UCI), N=252 (188 PD)	Sustained phonation of vowel /a/, Voice signals processed for acoustic feature extraction with 753 total features from sustained phonation	Machine Learning	Support Vector Machine with grid search optimization	Acoustic features (753 features: Jitter variants, Shimmer, Fundamental frequency, Harmonicity, RPDE, DFA, PPE, Intensity, Formant frequencies, Bandwidth, GQ, GNE, VFER, EMD, MFCCs, WT, TQWT) ∣ SMOTE oversampling + Recursive Feature Elimination (RFE) + Principal Component Analysis (PCA) reducing to 18 features	Train-test split with grid search hyperparameter tuning	SVM (RFE + PCA): Accuracy = 98.2%, Precision = 0.99, Sensitivity = 0.97, Specificity = 0.99, G-Mean = 0.98.<br><br>MLP: Accuracy = 96.4%, Sensitivity = 0.94. Bagging: Accuracy = 95.1%. KNN: Accuracy = 93.8%.<br><br>RFE + PCA reduced features from 753 → 18 with minimal loss and improved accuracy by ~11–13%.	Small number of patients (188 PD vs 64 HC) creating class imbalance, lack of laboratory validation results, highly distributed features causing noise and increased processing time
**Quan et al. (2022) [26]**	Two datasets:<br>PC-GITA Database, N=100 (50 PD)<br>Chinese PD Speech Dataset(GYENNO), N= 45 (30 PD)	Sustained vowel /a/, short sentence reading (/si shi si zhi shi shi zi/), multiple Spanish vowels (/a/, /u/), words (/apto/, /gato/), simple/complex sentences	Deep Learning	FedOcw (Optimized Client Weighting Federated Learning): Dynamically assigns personalized weights to each client node to adapt to data heterogeneity. Integrates a Time-Distributed 2D-CNN + 1D-CNN hybrid architecture per client.	Log Mel-spectrograms with 64 Mel-frequency bands ∣ Time-distributed 2D-CNNs for temporal feature extraction followed by 1D-CNN for dependency capture	Database-1: 80/20 train-test split, Database-2: cross-validation with 10 runs	Database 1 (Chinese): Vowel /a/: Accuracy = 81.6 %, F-score = 87.7 %, Sensitivity = 79.2 %, Specificity = 98.3 %. Sentence /si shi si zhi shi shi zi/: Accuracy = 75.3 %, F-score = 83.6 %.<br><br>Database 2 (Spanish): Vowel /a/: 85.3 %; Word /apto/: 81.5 %; Simple sentence /loslibros/: 92 %; Complex /viste/: 92 %.<br><br>Cross-database (Chinese → Spanish / Spanish → Chinese): Accuracy ≈ 50–71 %.	Cross-database evaluation showed reduced performance (49.4% Chinese→Spanish), limited to specific speech tasks, different recording conditions affecting generalizability

Author (Year)	Datasets used	Voice Task	Model Type	Specific Model	Input Data Type ∣Features Ext. Method	Training/validation Method	Best Performance Metrics	Limitations
**Quan et al. (2025) [39]**	Four datasets:<br>Italian Parkinson's Voice and Speech (ItalianPVS), N=50 (28 PD)<br>PC-GITA Dataset, N=100 (50 PD)<br>Chinese PD Speech Dataset(GYENNO), N= 45 (30 PD)<br>MDVR-KCL Dataset , N=37 (16 PD)	Sustained vowels, sentence reading, syllable repetition, spontaneous speech (varies by dataset)	Deep Learning	FedOcw (Optimized Client Weights Federated Learning) with Time-distributed 2D-CNN + 1D-CNN	Log Mel-spectrograms ∣ Librosa with 22.05 kHz resampling, 512 hop length, 55 Mel bands, zero-padding for uniform dimensions	Federated learning with 100 aggregation rounds, 80/20 train-test split, speaker-independent validation	Best Performance Metrics (FedOcw):<br>Scenario A (Spanish–Italian): Accuracy = 74.81 %, F1 = 73.35 %, MCC = 0.502.<br>Scenario B (Spanish–Chinese): Accuracy = 67.85 %, F1 = 61.8 %, MCC = 0.288.<br>Scenario C (Italian–Chinese): Accuracy = 82.78 %, F1 = 81.93 %, Specificity = 84.19 %, Sensitivity = 83.44 %, MCC = 0.676.<br>Scenario D (Spanish–Italian–Czech): Accuracy = 72.53 %, F1 = 69.8 %, MCC = 0.465.<br><br>Scenario E (Five-Language): Accuracy = 72.63 %, F1 = 68.16 %, MCC = 0.435.	Data heterogeneity across institutions with varying recording conditions and linguistic content, limited evaluation scenarios with predefined client-dataset mappings, privacy constraints limiting data sharing for validation
**Rahmatallah et al. (2025) [72]**	Two datasets:<br>Telephone PD Voice Dataset (UAMS) , N=81 (40 PD)<br>mPower Dataset, subsample, N= 398 (188 PD)	Sustained vowel /a/ recordings via analog telephone lines (UAMS) and smartphone app (mPower) trimmed to 1.5 seconds for spectrogram generation	Deep Learning	Pre-trained Inception V3 CNN with transfer learning	Mel-scale and linear-scale spectrograms ∣ Hanning window (1024 samples linear, 512 samples mel), FFT processing, normalization to Rehman-et-al.-2023-Parkinsons-Disease-Detection-Using-Hybrid-LSTM-GRU-Deep-Learning-Model-169774.pdf range	70/30 train-test split with 100 repeated cross-validation iterations	CNN (Inception V3 with transfer learning):<br>UAMS:<br>Linear spectrogram AUC = 0.95; Mel spectrogram AUC = 0.97<br><br>mPower:<br>Linear spectrogram AUC = 0.92; Mel spectrogram AUC = 0.95	Limited bandwidth of analog telephone lines affecting frequency content, small dataset sizes constraining deep learning performance, potential recording quality variations across different platforms
**Rehman et al. (2023) [73]**	Oxford PD Speech Dataset (UCI), N=31 (23 PD)	Sustained phonation of vowel /a/	Deep Learning	Hybrid LSTM-GRU model	22 acoustic features (MDVP fundamental frequency, jitter, shimmer, harmonicity measures, RPDE, DFA, spread parameters, PPE) ∣ Standard scaling with Random Oversampling and SMOTE for class balancing	70 % train / 30 % test and also whole-dataset cross-testing.	Accuracy: 100% (Random Oversampling), 98% (SMOTE), Precision: 100% (Random Oversampling), 100% (SMOTE), Recall: 100% (Random Oversampling), 97% (SMOTE), F1: 100% (Random Oversampling), 98% (SMOTE), AUC: 99% (SMOTE)	Small dataset size (N=31) limiting generalizability, highly imbalanced classes requiring extensive oversampling, lack of independent validation dataset, potential overfitting due to small sample size

Author (Year)	Datasets used	Voice Task	Model Type	Specific Model	Input Data Type ǀFeatures Ext. Method	Training/validation Method	Best Performance Metrics	Limitations
**Rey-Paredes et al. (2025) [74]**	PC-GITA Dataset, N=100 (50 PD)	Sustained phonation of vowel /a/ recorded three times per individual (300 total recordings)	Deep Learning	CDIL-CNN (Circular Dilated Convolutional Neural Network), ResNet, LSTM-FCN, InceptionTime	Raw voice waveforms trimmed to 480ms (11,520 timesteps) ǀ Min-max normalization with BigVSAN GAN for data augmentation using randomization factor 10^-5	60-20-20 speaker-independent stratified 5-fold cross-validation with synthetic data for training/validation, real data for testing	CDIL-CNN: Accuracy: 73%, Sensitivity: 68.5%, Specificity: 77.5%, F1: 71.8%, AUC: 73%	Limited dataset size constraining deep learning generalizability, high similarity between synthetic and original samples potentially limiting model learning capacity, models trained with waveforms showing inherent complexity compared to spectrogram approaches
**Scimeca et al. (2023) [29]**	Five Datasets:<br>Italian Parkinson's Voice and Speech (ItalianPVS), N=50 (28 PD)<br>Italian Torino corpus-1, N=30 (17 PD)<br>Italian Torino corpus-2, N=30 (15 PD)<br>Italian Rome corpus, *earlier version, N=102 (46 PD)<br>CzechPD-APS Dataset, N=44 (22 PD)	Sustained vowels /a/, /e/ and phonetically balanced sentences recorded via professional microphones/smartphones in variable acoustic conditions	Machine Learning	k-Nearest Neighbors (kNN), Gaussian Process (GP), Random Forest, SVM, XGBoost	Acoustic features (F0, Formants 1-3, Jitter variants, Shimmer variants, Intensity, HNR, CPP, GNE, Spectral features, MFCCs 1-13 with delta/delta-delta, PLP 1-13 with derivatives, DFA) ǀ Praat-based extraction with 25ms windows, 10ms overlap, min-max normalization	70/30 train-test split with 10-fold cross-validation, speaker-independent splits, Boruta feature selection	Experiment 1 (Speech protocol):<br><br>Best protocol = /a/ + sentence combination, achieving 91% accuracy (AUC ≈ 0.90).<br><br>Single vowels ≈ 78–84%; sentences alone ≈ 86–88%; combined tasks improved by +13.6%.<br><br>kNN: Acc = 91.3%, F1 = 88–90%; GP: Acc = 88.7–94.5%, F1 = 88.9%.<br><br>Experiment 2 (Cross-lingual robustness):<br><br>XGBoost: Accuracy = 71.8% (CV), 64.9% (test).<br><br>Random Forest: Accuracy = 70.2% (CV), 70.1% (test), F1 = 71.6%.<br><br>Including co-variables (language, gender) improved generalization.	Dataset heterogeneity impacting performance when merging corpora, small sample sizes in some individual datasets, limited to binary classification without disease staging, suboptimal recording conditions in some datasets
**Sedigh Malekroodi et al. (2025) [75]**	NeuroVoz dataset, N=112 (54 PD)	16 repeated utterances of sentences	Deep Learning	Fine-tuned Wav2Vec 2.0 and HuBERT with Supervised Contrastive Learning (SupCon)	Pre-trained ASR features (768-dimensional embeddings) ǀ Raw audio preprocessed to 16 kHz, 5-second standardization, silent segment removal, low-pass filtering augmentation	5-fold cross-validation with 70/20/10 train/validation/test split	Wav2Vec 2.0 + SupCon: F1: 90.0%, AUC: 0.92<br>HuBERT + SupCon: F1: 88.99%, AUC: 0.91	Single-language dataset (Spanish only) limiting cross-linguistic generalizability, relatively small cohort size, potential linguistic variability effects on model performance requiring further dedicated research

Author (Year)	Datasets used	Voice Task	Model Type	Specific Model	Input Data Type ǀFeatures Ext. Method	Training/validation Method	Best Performance Metrics	Limitations
**Shen et al. (2025) [76]**	Telephone PD Voice Dataset (UAMS) , N=81 (40 PD)	Voice recordings with background noise removal, decibel equalization	Deep Learning	Hybrid MLP + CNN + RNN + MKL ensemble model	Acoustic features (MFCCs 1-13, Jitter variants, Shimmer, Pitch statistics, HNR) ǀ Parselmouth/Praat feature extraction with Fourier transformation and standardization	5-fold cross-validation with 75/25 train-test splits, 150 epochs with early stopping and learning rate reduction	Accuracy: 91.11%, Precision: 89.84%, Recall: 92.50%, F1: 91.13%, AUC: 0.9125	Small dataset size (N=81) limiting generalizability, model complexity potentially causing overfitting, single-language recordings limiting cross-linguistic applicability, uncertainty about longitudinal disease progression tracking capability
**Singh and Tripathi (2024) [77]**	Three datasets:<br>Oxford PD Speech Dataset (UCI), N=31 (23 PD)<br>Istanbul PD Speech Dataset (UCI), N=252 (188 PD)<br>Parkinson Dataset with Replicated Acoustic Features-UCI, N= 80 (40 PD)	Voice recordings with sustained vowel /a/ and various acoustic feature extraction methods	Machine Learning	Ensemble Feature Selection Algorithm (EFSA) with KNN, Random Forest, Decision Tree, SVM, Bagging, MLP, Gradient Boosting	Multi-dataset features (Dataset-I: 13/23 selected features, Dataset-II: 45/754 selected features, Dataset-III: 17/46 selected features) ǀ Filter, wrapper, and embedding algorithms with SMOTE oversampling	10-fold cross-validation with hyperparameter optimization	Dataset-I: Accuracy: 97.6%, F1: 97.9%, Precision: 98%, Recall: 98% Dataset-II: Accuracy: 90.2%, F1: 90.2%, Precision: 90.2%, Recall: 90.5% Dataset-III: Accuracy: 83.3%, F1: 83.3%, Precision: 83.5%, Recall: 83.3%	High-dimensional datasets requiring extensive feature selection, class imbalance requiring SMOTE intervention, computational complexity challenges with multiple ensemble components
**Srinivasan et al. (2024) [78]**	Oxford PD Speech Dataset (UCI), N=31 (23 PD)	Voice recordings with sustained vowel /a/	Deep Learning	Feed-forward Neural Network (FNN), Kernel Support Vector Machine (KSVM), Random Forest, Decision Tree, K-Nearest Neighbor	Acoustic features (24 features: MDVP F0, Jitter variants, Shimmer variants, HNR, NHR, RPDE, DFA, spread measures, PPE) ǀ StandardScaler normalization with SelectKBest feature selection (top 8 features) and SMOTE oversampling	80/20 train-test split with RandomizedSearchCV hyperparameter optimization	FNN: Accuracy: 99.11%, Precision: 99.96%, Recall: 98.78%, F1: 99.23%<br>KSVM: Accuracy: 95.89%, Precision: 98.71%, Recall: 96.88%, F1: 97.62%	Small dataset size (N=31 patients) limiting generalizability, class imbalance requiring oversampling, lack of clinical validation, reliance solely on voice signals may not capture all PD symptom nuances
**Suppa et al. (2022) [30]**	Italian Rome Corpus, earlier version, N=223 (115 PD)	Sustained vowel /e/ (5 seconds) and standardized Italian sentence recorded	Machine Learning	Support Vector Machine with linear kernel	OpenSMILE feature extraction (6,139 features from IS ComParE 2016) ǀ Correlation Features Selection (CFS) with Information Gain Attribute Evaluation, discretization, top 30 features selected	10-fold cross-validation (stratified by class) with hyperparameter optimization using sequential minimal optimization	HS vs PD (all): AUC = 0.870 (vowel), 0.848 (sentence); Accuracy = 79.6%<br>HS vs early PD: AUC = 0.900 (vowel), 0.876 (sentence); Accuracy = 81.5%.<br>HS vs mid-advanced PD (OFF): AUC = 0.980 (vowel), 0.897 (sentence); Accuracy = 93.5%.	Age differences between subgroups, potential daily vocal fluctuations not captured, single recording per patient may not represent typical vocal patterns, uncertain association between specific dysarthria aspects and selected features
**Tougui et al. (2024) [79]**	mPower dataset, subsample, N=1841 (363 PD)	Sustained phonation of vowel /a/ recorded via smartphone app in uncontrolled everyday environments with variable recording conditions	Deep Learning	Audio Spectrogram Transformer (AST) with transfer learning fine-tuning	Log-mel spectrograms (64 mel bins, 16ms hop length) ǀ Pre-trained AudioSet weights with fine-tuning, subject-wise and record-wise validation splits	5-fold cross-validation with mean/max/majority voting aggregation strategies	Best Performance Metrics (AST Model): Subject-wise split (with transfer learning): Accuracy= 91.35%, AUC = 95.89 % ± 1.07	Self-reported data introducing environmental variability, device differences affecting recording quality, model interpretability challenges for clinical adoption, validation needed across multiple datasets and languages

Author (Year)	Datasets used	Voice Task	Model Type	Specific Model	Input Data Type ∣Features Ext. Method	Training/validation Method	Best Performance Metrics	Limitations
**Valarmathi et al. (2025) [80]**	Italian Parkinson's Voice and Speech (ItalianPVS), N=50 (28 PD)	Sustained phonations and short speech tasks	Deep Learning	Feature-Based Deep Neural Network (FB-DNN: Stacked Autoencoder + DNN)	Raw audio ∣ Modified Band Pass Filter preprocessing + Stacked Autoencoder (SAE) nonlinear hierarchical feature extraction	5-fold cross-validation with 1000-epoch DNN training and dropout regularization	Accuracy: 96.15%, Precision: 98%, Recall: 98%, F1: 98%, AUC-ROC: 95.6%	Limitations: Single small Italian dataset, no stage-specific evaluation, limited generalizability, controlled environment only, lacks cross-language validation, interpretability limited despite SHAP integration
**Vásquez-Correa et al. (2021) [81]**	Three Datasets:<br>PC-GITA Dataset, N=100 (50 PD)<br>German PD Speech Dataset, N=176 (88 PD),<br>Czech PD Speech Dataset, N= 100 (50 PD)	Onset and offset transitions (160ms segments) from voiced/unvoiced boundaries extracted from continuous speech tasks including sentences, text reading, monologues, and DDK tasks	Deep Learning	Convolutional Neural<br>Network with transfer learning (4 conv layers + max pooling + dropout + 2<br>FC layers)	Mel-scale spectrograms from STFT (256 frequency bins, 32ms window, 4ms step, 80 mel filters) forming 80×41 representations ∣ Time-frequency analysis of vocal fold transition segments	10-fold speaker-independent cross-validation with fine-tuning transfer learning strategy	Baseline (SVM):<br><br>PD–Spanish 74.0%, PD–German 69.9%, PD–Czech 70.2%, HD–Czech 85.2%, LP 94.3%.<br><br>CNN Base Models (no transfer):<br><br>PD–Spanish 71.0%, PD–German 63.1%, PD–Czech 68.5%, HD–Czech 81.3%, LP 98.2%.<br><br>Transfer Learning (Among Languages):<br><br>PD–Spanish → PD–German 77.3% Acc (AUC 0.82).<br><br>PD–Spanish → PD–Czech 76.7–72.6% Acc (AUC 0.79–0.83).<br><br>PD–Czech+German → PD–Spanish 75.0% Acc.	Transfer learning improves accuracy only when base model is robust enough, limited cross-language generalizability depending on linguistic similarities and disease severity<br>differences, medication effects not fully controlled across datasets

Author (Year)	Datasets used	Voice Task	Model Type	Specific Model	Input Data Type |Features Ext. Method	Training/validation Method	Best Performance Metrics	Limitations
**Veetil et al. (2024)[82]**	Dataset1: PC-GITA Dataset, N=100 (50 PD)<br>Dataset2: Italian Parkinson's Voice and Speech database, N=50 (28 PD)	Sustained phonations of vowels /a/, /e/, /i/, /o/, /u/ recorded in soundproof conditions with 3 repetitions per vowel for Spanish and 2 for Italian Sustained and modulated vowels at natural pitch and loudness.<br>Each utterance ~1 s segments; normalized	Deep Learning	Custom 1D-CNN classifier with raw VMD Intrinsic Mode Functions	Variational Mode Decomposition (VMD) with 10 modes (α=120 for Spanish, α=60 for Italian) | Raw IMF signals from VMD decomposition used directly as input without traditional feature extraction	10-fold cross-validation (speaker-level independence)., Gender-independent and gender-specific models with cross-lingual validation and independent dataset testing	Best Performance Metrics:<br><br>Cross-lingual:<br><br>Train (Spanish PC-GITA) → Test (Italian PD): Accuracy = 80% (gender-specific CNN).<br><br>Train (Italian PD) → Test (Spanish PC-GITA): 65–72.5%.<br><br>Same-language (within dataset):<br><br>Spanish PC-GITA (Sustained vowels): Acc = 90%.<br><br>Spanish PC-GITA (Modulated vowels): Acc = 95% (gender-specific).<br><br>Italian PD dataset: Acc = 90% (CNN, MLP).	Language dependency limiting universal applicability, small dataset sizes constraining deep learning performance, recording condition variations affecting generalizability across different acoustic environments
**Velu and Jaisankar (2025) [83]**	Istanbul PD Speech Dataset (UCI), N=252 (188 PD)	Voice recordings with 753 extracted acoustic features from sustained phonation	Machine Learning	Explainable balanced Recursive Feature Importance with Logistic Regression (XRFILR)	Acoustic features (753 features: Jitter variants, Shimmer variants, F0 measures, Formant frequencies, MFCC, TQWT, Vocal fold features, Harmonicity measures, RPDE, DFA, PPE) | KMeansSMOTE for class balancing with Recursive Feature Elimination (RFE) selecting top 50 features	80/20 train-test split with hyperparameter optimization using cross-validation	Proposed XRFILR model:<br>Accuracy = 96.46 %,<br>Precision = 96.5 %,<br>Recall = 96.5 %, F1 = 0.965, AUC = 0.99.	Class imbalance requiring oversampling techniques, high dimensionality necessitating feature selection, computational complexity of preprocessing steps, potential overfitting with small dataset size
**Wang et al. (2022) [24]**	Mandarin PD Speech Dataset (HUST), N=100 (50 PD)	Sustained vowels /a/, /e/ (6-second stable segments) and tongue twisters ("si shi si zhi shi shi zi", "yi zhi da hua wan kou zhe yi zhi da hua ha ma") recorded 3 times each using Rode NT-USB microphone at 96 kHz sampling rate	Machine Learning	Stochastic Gradient Descent (SGD), Logistic Regression (LR), K-Nearest Neighbor (KNN), Naïve Bayes	Acoustic features (Phonation: 28 features including Jitter, Shimmer, APQ, PPQ, DF0, DDF0, LogE Articulation: 488 features including BBE, MFCC, F1/F2 formants and derivatives) | LASSO, mRMR, Relief-F feature selection with spectral subtraction noise reduction	10-fold cross-validation with 90/10 train-test split	Articulation-based (tongue twisters):<br>Best combination (LASSO+SGD):<br>Accuracy: 75.76%,<br>Sensitivity: 82.44%,<br>Specificity: 73.15%,<br>Precision: 76.57%, F1: 79%	HY1-3 stage patients with relatively low dysarthria severity, high-dimensional feature sets potentially causing overfitting, Chinese language-specific characteristics affecting speech pattern analysis compared to English

Author (Year)	Datasets used	Voice Task	Model Type	Specific Model	Input Data Type ∣Features Ext. Method	Training/validation Method	Best Performance Metrics	Limitations
**Wang et al. (2024) [25]**	Chinese Mild-PD Voice Dataset, N=278 (139 PD; 60 mild, mH&Y ≤1.5)	Sustained vowels /a/, /o/, /i/ recorded using smartphone app (GYENNO PD Assistant) in quiet environment for 3 seconds each	Machine Learning	Support Vector Machine with RBF kernel	Voice features (Phonation: Jitter, Shimmer variants Articulation: MFCC, BBE Prosody: Fundamental frequency measures Representation learning: Bottleneck features from deep autoencoder, MSE features) ∣ Feature selection using Recursive Feature Elimination with cross-validation (RFECV)	Train/Test Split: 70 : 30 (train : test) for both full PD and mild PD models.<br><br>Cross-Validation: 10-fold CV × 5 repetitions for feature selection + hyperparameter tuning.	Mild PD vs HC: Accuracy: 85%, Sensitivity: 95%, Specificity: 75%, AUC: 0.93 (95% CI: 0.85-1.00) All PD vs HC: Accuracy: 93%, Sensitivity: 100%, Specificity: 85%,AUC: 0.99 (95% CI: 0.98–1.00)	Single-center study limiting generalizability, relatively small sample size for mild PD subgroup (N=60), potential recording quality variations using smartphone devices, limited to Chinese-speaking population
**Xu et al. (2020) [84]**	Istanbul PD Speech Dataset (UCI), N=252 (188 PD)	Sustained vowel phonations (/a/, /o/, /u/) recorded usi6 seconds each with 3 consecutive pronunciations, divided into 2-second fragments	Deep Learning	S-DCGAN (Spectrogram Deep Convolutional Generative Adversarial Network) with ResNet50 classifier	Spectrograms (256×256×3 resolution) generated using STFT with Hamming window, 2048-point FFT, 46.44ms frame length ∣ GAN-based data augmentation with SSIM (>0.80) and PSNR (>28dB) selection criteria	10-fold sample expansion with traditional image enhancement (horizontal/vertical flipping, brightness/noise adjustment)	Accuracy: 91.25%, Specificity: 92.5% (S-DCGAN-ResNet50 vs 85.5% DCGAN-ResNet50)	Limited dataset size requiring extensive augmentation, quality dependency on generated sample similarity metrics, potential overfitting due to synthetic data dominance
**Xu et al. (2025) [85]**	Oxford PD Speech Dataset (UCI), N=31 (23 PD)	Voice recordings (approximately 6 per patient),	Machine Learning	Random Forest, Gradient Boosting Machine (GBM), Support Vector Machine, Multi-layer Perceptron, Decision Trees, Logistic Regression, Naive Bayes, LightGBM	Acoustic features (22 features: MDVP F0/Fhi/Flo, Jitter variants, Shimmer variants, NHR, HNR, RPDE, DFA, spread1/spread2, D2, PPE) ∣ LASSO regression with independent t-test/Mann-Whitney U test, Pearson correlation analysis for feature selection reducing to 11 optimal features	70/30 train-test split with 10-fold cross-validation and SMOTE oversampling for class imbalance	Random Forest (RF): AUC = 0.967 (train) ∣ 0.936 (test) Accuracy = 87.2 % (train) ∣ 85.2 % (test) Sensitivity = 88.6 % (train) ∣ 87.5 % (test) Specificity = 96.7 % (train) ∣ 90.6 % (test) F1 = 0.863 (test)	Small dataset size (N=31) limiting generalizability, class imbalance requiring oversampling, lack of external validation on independent datasets
**Yang et al. (2025) [86]**	Istanbul PD Speech Dataset (UCI), N=252 (188 PD)	Three sustained phonations of vowel /a/ per participant	Machine Learning	XGBoost with post-mean aggregation	754 acoustic features (Time-frequency, MFCCs, Wavelet transform, Vocal fold, TQWT features) ∣ Multiple Instance Learning with four data aggregation methods (post-mean, post-max, post-min, pre-mean) and Bag Over-Sampling (BOS) for class imbalance	70/30 train-test split with 5-fold cross-validation and grid search hyperparameter optimization	Post-mean XGBoost: Accuracy: 88.0%, Precision: 90.4%, Sensitivity: 94.2%, Specificity: 69.5%, F1: 92.2%, AUC: 90.7%, MCC: 67.2%	Class imbalance requiring oversampling techniques, grouped data structure requiring specialized aggregation methods, potential information loss in bag-based aggregation approaches

Author (Year)	Datasets used	Voice Task	Model Type	Specific Model	Input Data Type ∣Features Ext. Method	Training/validation Method	Best Performance Metrics	Limitations
**Zahid et al. (2020) [87]**	PC-GITA Dataset, N=100 (50 PD)	Sustained vowels /a/, /e/, /i/, /o/, /u/, monologues, read text, and words (/apto/, /atelta/) recorded in Spanish	Deep Learning	Three approaches: (1) Transfer learning with AlexNet, (2) Deep features extracted from spectrograms using AlexNet + ML classifiers (SVM, RF, MLP), (3) Handcrafted acoustic features + ML classifiers	Spectrograms (224×224×3 RGB) generated using STFT with Hamming window, 2048-point FFT, 46.44ms frame length ∣ Signal preprocessing with spectrogram conversion and deep feature extraction using pre-trained AlexNet (features from first 5 convolutional layers)	5-fold cross-validation for machine learning approaches, transfer learning with various epoch sizes (6-10) and weighted learning rates (30-70)	Transfer Learning (AlexNet fine-tuned): Vowels: /u/ = 82.3%, /i/ = 81.4%, /o/ = 72.6%. Read text = 91%, Monologue = 86.3%.<br><br>Deep Feature + ML: MLP: Accuracy = 99.7% (vowels /e/, /i/, /o/); 99.3% (monologue, read text).<br>Random Forest: 99.1–99.4% (vowels), 97% (read text), 97.8% (monologue).<br>Transfer learning: 91% accuracy (read text)	Not reported

References

1. Orozco-Arroyave, J.R.; Arias-Londoño, J.D.; Vargas-Bonilla, J.F.; González-Rátiva, M.C.; Nöth, E. New Spanish Speech Corpus Database for the Analysis of People Suffering from Parkinson's Disease. In Proceedings of the Proceedings of the Ninth International Conference on Language Resources and Evaluation (LREC'14); Calzolari, N., Choukri, K., Declerck, T., Loftsson, H., Maegaard, B., Mariani, J., Moreno, A., Odijk, J., Piperidis, S., Eds.; European Language Resources Association (ELRA): Reykjavik, Iceland, May 2014; pp. 342–347.
2. Adnan, T.; Abdelkader, A.; Liu, Z.; Hossain, E.; Park, S.; Islam, M.S.; Hoque, E. A Novel Fusion Architecture for Detecting Parkinson's Disease Using Semi-Supervised Speech Embeddings. *npj Parkinsons Dis.* **2025**, *11*, 176, doi:10.1038/s41531-025-00956-7.
3. Naranjo, L.; Pérez, C.J.; Martín, J. Addressing Voice Recording Replications for Tracking Parkinson's Disease Progression. *Med. Biol. Eng. Comput.* **2016**, *55*, 365–373, doi:10.1007/s11517-016-1512-y.
4. Sakar, B.E.; Isenkul, M.E.; Sakar, C.O.; Sertbas, A.; Gurgen, F.; Delil, S.; Apaydin, H.; Kursun, O. Collection and Analysis of a Parkinson Speech Dataset With Multiple Types of Sound Recordings. *IEEE J. Biomed. Health Inform.* **2013**, *17*, 828–834, doi:10.1109/JBHI.2013.2245674.
5. Little, M.A.; McSharry, P.E.; Roberts, S.J.; Costello, D.A.; Moroz, I.M. Exploiting Nonlinear Recurrence and Fractal Scaling Properties for Voice Disorder Detection. *Biomed. Eng. Online* **2007**, *6*, 23, doi:10.1186/1475-925X-6-23.
6. Tsanas, A.; Little, M.A.; McSharry, P.E.; Ramig, L.O. Accurate Telemonitoring of Parkinson's Disease Progression by Noninvasive Speech Tests. *IEEE Trans. Bio-Med. Eng.* **2010**, *57*, 884–893, doi:10.1109/TBME.2009.2036000.
7. Mendes-Laureano, J.; Gómez-García, J.A.; Guerrero-López, A.; Luque-Buzo, E.; Arias-Londoño, J.D.; Grandas-Pérez, F.J.; Godino-Llorente, J.I. NeuroVoz: A Castillian Spanish Corpus of Parkinsonian Speech. *Sci. Data* **2024**, *11*, 1367, doi:10.1038/s41597-024-04186-z.
8. Dimauro, G.; Di Nicola, V.; Bevilacqua, V.; Caivano, D.; Girardi, F. Assessment of Speech Intelligibility in Parkinson's Disease Using a Speech-To-Text System. *IEEE Access* **2017**, *5*, 22199–22208, doi:10.1109/ACCESS.2017.2762475.
9. Dimauro, G.; Caivano, D.; Bevilacqua, V.; Girardi, F.; Napoletano, V. VoxTester, Software for Digital Evaluation of Speech Changes in Parkinson Disease. In Proceedings of the 2016 IEEE International Symposium on Medical Measurements and Applications (MeMeA); IEEE Press: Benevento, Italy, May 2016; pp. 1–6.
10. Bot, B.M.; Suver, C.; Neto, E.C.; Kellen, M.; Klein, A.; Bare, C.; Doerr, M.; Pratap, A.; Wilbanks, J.; Dorsey, E.R.; et al. The mPower Study, Parkinson Disease Mobile Data Collected Using ResearchKit. *Sci. Data* **2016**, *3*, 160011, doi:10.1038/sdata.2016.11.
11. Lim, W.S.; Chiu, S.-I.; Peng, P.-L.; Jang, J.-S.R.; Lee, S.-H.; Lin, C.-H.; Kim, H.-J. A Cross-Language Speech Model for Detection of Parkinson's Disease. *J. Neural Transm.* **2024**, *132*, 579–590, doi:10.1007/s00702-024-02874-z.
12. Jeong, S.-M.; Song, Y.-D.; Seok, C.-L.; Lee, J.-Y.; Lee, E.C.; Kim, H.-J. Machine Learning-Based Classification of Parkinson's Disease Using Acoustic Features: Insights from Multilingual Speech Tasks. *Comput. Biol. Med.* **2024**, *182*, 109078, doi:10.1016/j.compbiomed.2024.109078.
13. Favaro, A.; Tsai, Y.-T.; Butala, A.; Thebaud, T.; Villalba, J.; Dehak, N.; Moro-Velázquez, L. Interpretable Speech Features vs. DNN Embeddings: What to Use in the Automatic Assessment of Parkinson's Disease in Multi-Lingual Scenarios. *Comput. Biol. Med.* **2023**, *166*, 107559, doi:10.1016/j.compbiomed.2023.107559.
14. Pinto, S.; Cardoso, R.; Sadat, J.; Guimarães, I.; Mercier, C.; Santos, H.; Atkinson-Clement, C.; Carvalho, J.; Welby, P.; Oliveira, P.; et al. Dysarthria in Individuals with Parkinson's Disease: A Protocol for a Binational, Cross-Sectional, Case-Controlled Study in French and European Portuguese (FraLusoPark). *BMJ Open* **2016**, *6*, e012885, doi:10.1136/bmjopen-2016-012885.
15. Bocklet, T.; Steidl, S.; Nöth, E.; Skodda, S. Automatic Evaluation of Parkinson's Speech — Acoustic, Prosodic and Voice Related Cues. In Proceedings of the Interspeech 2013; ISCA, August 25 2013; pp. 1149–1153.

16. Rusz, J.; Cmejla, R.; Tykalova, T.; Ruzickova, H.; Klempir, J.; Majerova, V.; Picmausova, J.; Roth, J.; Ruzicka, E. Imprecise Vowel Articulation as a Potential Early Marker of Parkinson's Disease: Effect of Speaking Task. *J. Acoust. Soc. Am.* **2013**, *134*, 2171–2181, doi:10.1121/1.4816541.
17. Klempíř, O.; Příhoda, D.; Krupička, R. Evaluating the Performance of Wav2vec Embedding for Parkinson's Disease Detection. *Meas. Sci. Rev.* **2023**, *23*, 260–267, doi:10.2478/msr-2023-0033.
18. Hlavnička, J.; Čmejla, R.; Klempíř, J.; Růžička, E.; Rusz, J. Acoustic Tracking of Pitch, Modal, and Subharmonic Vibrations of Vocal Folds in Parkinson's Disease and Parkinsonism. *IEEE Access* **2019**, *7*, 150339–150354, doi:10.1109/ACCESS.2019.2945874.
19. Jeancolas, L.; Petrovska-Delacrétaz, D.; Mangone, G.; Benkelfat, B.-E.; Corvol, J.-C.; Vidailhet, M.; Lehéricy, S.; Benali, H. X-Vectors: New Quantitative Biomarkers for Early Parkinson's Disease Detection From Speech. *Front. Neuroinf.* **2021**, *15*, 578369, doi:10.3389/fninf.2021.578369.
20. Virmani, T.; Lotia, M.; Glover, A.; Pillai, L.; Kemp, A.S.; Iyer, A.; Farmer, P.; Syed, S.; Larson-Prior, L.J.; Prior, F.W. Feasibility of Telemedicine Research Visits in People with Parkinson's Disease Residing in Medically Underserved Areas. *J. Clin. Transl. Sci.* **2022**, *6*, e133, doi:10.1017/cts.2022.459.
21. Vaiciukynas, E.; Verikas, A.; Gelzinis, A.; Bacauskiene, M. Detecting Parkinson's Disease from Sustained Phonation and Speech Signals. *PLOS One* **2017**, *12*, e0185613, doi:10.1371/journal.pone.0185613.
22. Jaeger, H.; Dhaval Trivedi; Stadtschnitzer, M. Mobile Device Voice Recordings at King's College London (MDVR-KCL) from Both Early and Advanced Parkinson's Disease Patients and Healthy Controls 2019.
23. Viswanathan, R.; Khojasteh, P.; Aliahmad, B.; Arjunan, S.P.; Ragnav, S.; Kempster, P.; Wong, K.; Nagao, J.; Kumar, D.K. Efficiency of Voice Features Based on Consonant for Detection of Parkinson's Disease. In Proceedings of the 2018 IEEE Life Sciences Conference (LSC); Montreal, QC, October 2018; pp. 49–52.
24. Wang, Q.; Fu, Y.; Shao, B.; Chang, L.; Ren, K.; Chen, Z.; Ling, Y. Early Detection of Parkinson's Disease from Multiple Signal Speech: Based on Mandarin Language Dataset. *Front. Aging Neurosci.* **2022**, *14*, 1036588, doi:10.3389/fnagi.2022.1036588.
25. Wang, M.; Zhao, X.; Li, F.; Wu, L.; Li, Y.; Tang, R.; Yao, J.; Lin, S.; Zheng, Y.; Ling, Y.; et al. Using Sustained Vowels to Identify Patients with Mild Parkinson's Disease in a Chinese Dataset. *Front. Aging Neurosci.* **2024**, *16*, 1377442, doi:10.3389/fnagi.2024.1377442.
26. Quan, C.; Ren, K.; Luo, Z.; Chen, Z.; Ling, Y. End-to-End Deep Learning Approach for Parkinson's Disease Detection from Speech Signals. *J. Appl. Biomed.* **2022**, *42*, 556–574, doi:10.1016/j.bbe.2022.04.002.
27. Quan, C.; Chen, Z.; Ren, K.; Luo, Z. FedOcw: Optimized Federated Learning for Cross-Lingual Speech-Based Parkinson's Disease Detection. *npj Digital Med.* **2025**, *8*, 357, doi:10.1038/s41746-025-01763-3.
28. Amato, F.; Borzi, L.; Olmo, G.; Artusi, C.A.; Imbalzano, G.; Lopiano, L. Speech Impairment in Parkinson's Disease: Acoustic Analysis of Unvoiced Consonants in Italian Native Speakers. *IEEE Access* **2021**, *9*, 166370–166381, doi:10.1109/ACCESS.2021.3135626.
29. Scimeca, S.; Amato, F.; Olmo, G.; Asci, F.; Suppa, A.; Costantini, G.; Saggio, G. Robust and Language-Independent Acoustic Features in Parkinson's Disease. *Front. Neurol.* **2023**, *14*, 1198058, doi:10.3389/fneur.2023.1198058.
30. Suppa, A.; Costantini, G.; Asci, F.; Di Leo, P.; Al-Wardat, M.S.; Di Lazzaro, G.; Scalise, S.; Pisani, A.; Saggio, G. Voice in Parkinson's Disease: A Machine Learning Study. *Front. Neurol.* **2022**, *13*, 831428, doi:10.3389/fneur.2022.831428.
31. Giuliano, M.; Fernandez, L.; Pérez, S. Selección de Medidas de Disfonía Para La Identificación de Enfermos de Parkinson [Not Available in English]. In Proceedings of the 2020 IEEE Congreso Bienal de Argentina (ARGENCON); Resistencia, Argentina, December 1 2020; pp. 1–8.
32. Alalayah, K.M.; Senan, E.M.; Atlam, H.F.; Ahmed, I.A.; Shatnawi, H.S.A. Automatic and Early Detection of Parkinson's Disease by Analyzing Acoustic Signals Using Classification Algorithms Based on Recursive Feature Elimination Method. *Diagnostics* **2023**, *13*, 1924, doi:10.3390/diagnostics13111924.

33. Ali, L.; Javeed, A.; Noor, A.; Rauf, H.T.; Kadry, S.; Gandomi, A.H. Parkinson's Disease Detection Based on Features Refinement through L1 Regularized SVM and Deep Neural Network. *Sci. Rep.* **2024**, *14*, 1333, doi:10.1038/s41598-024-51600-y.
34. Alshammri, R.; Alharbi, G.; Alharbi, E.; Almubark, I. Machine Learning Approaches to Identify Parkinson's Disease Using Voice Signal Features. *Front. Artif. Intell.* **2023**, *6*, 1084001, doi:10.3389/frai.2023.1084001.
35. Bhatt, K.; Jayanthi, N.; Kumar, M. High-Resolution Superlet Transform Based Techniques for Parkinson's Disease Detection Using Speech Signal. *Appl. Acoust.* **2023**, *214*, 109657, doi:10.1016/j.apacoust.2023.109657.
36. Celik, G.; Başaran, E. Proposing a New Approach Based on Convolutional Neural Networks and Random Forest for the Diagnosis of Parkinson's Disease from Speech Signals. *Appl. Acoust.* **2023**, *211*, 109476, doi:10.1016/j.apacoust.2023.109476.
37. Chintalapudi, N.; Battineni, G.; Hossain, M.A.; Amenta, F. Cascaded Deep Learning Frameworks in Contribution to the Detection of Parkinson's Disease. *Bioengineering* **2022**, *9*, 116, doi:10.3390/bioengineering9030116.
38. Costantini, G.; Cesarini, V.; Di Leo, P.; Amato, F.; Suppa, A.; Asci, F.; Pisani, A.; Calculli, A.; Saggio, G. Artificial Intelligence-Based Voice Assessment of Patients with Parkinson's Disease Off and On Treatment: Machine vs. Deep-Learning Comparison. *Sens.* **2023**, *23*, 2293, doi:10.3390/s23042293.
39. Dao, Q.; Jeancolas, L.; Mangone, G.; Sambin, S.; Chalançon, A.; Gomes, M.; Lehéricy, S.; Corvol, J.-C.; Vidailhet, M.; Arnulf, I.; et al. Detection of Early Parkinson's Disease by Leveraging Speech Foundation Models. *IEEE J. Biomed. Health Inform.* **2025**, *29*, 5181–5190, doi:10.1109/JBHI.2025.3548917.
40. Escobar-Grisales, D.; Ríos-Urrego, C.D.; Orozco-Arroyave, J.R. Deep Learning and Artificial Intelligence Applied to Model Speech and Language in Parkinson's Disease. *Diagnostics* **2023**, *13*, 2163, doi:10.3390/diagnostics13132163.
41. Gimeno-Gómez, D.; Botelho, C.; Pompili, A.; Abad, A.; Martínez-Hinarejos, C.-D. Unveiling Interpretability in Self-Supervised Speech Representations for Parkinson's Diagnosis. *IEEE J. Sel. Top. Sign. Proces.* **2025**, 1–14, doi:10.1109/JSTSP.2025.3539845.
42. Hadjaidji, E.; Amara Korba, M.C.; Khelil, K. Improving Detection of Parkinson's Disease with Acoustic Feature Optimization Using Particle Swarm Optimization and Machine Learning. *Mach. Learn. Sci. Technol.* **2025**, *6*, 015026, doi:10.1088/2632-2153/adadc3.
43. Hawi, S.; Alhozami, J.; AlQahtani, R.; AlSafran, D.; Alqarni, M.; Sahmarany, L.E. Automatic Parkinson's Disease Detection Based on the Combination of Long-Term Acoustic Features and Mel Frequency Cepstral Coefficients (MFCC). *Biomed. Signal Process. Control* **2022**, *78*, 104013, doi:10.1016/j.bspc.2022.104013.
44. He, T.; Chen, J.; Xu, X.; Wang, W. Exploiting Smartphone Voice Recording as a Digital Biomarker for Parkinson's Disease Diagnosis. *IEEE Trans. Instrum. Meas.* **2024**, *73*, 1–12, doi:10.1109/TIM.2024.3391339.
45. Hireš, M.; Gazda, M.; Drotár, P.; Pah, N.D.; Motin, M.A.; Kumar, D.K. Convolutional Neural Network Ensemble for Parkinson's Disease Detection from Voice Recordings. *Comput. Biol. Med.* **2022**, *141*, 105021, doi:10.1016/j.compbiomed.2021.105021.
46. Hireš, M.; Drotár, P.; Pah, N.D.; Ngo, Q.C.; Kumar, D.K. On the Inter-Dataset Generalization of Machine Learning Approaches to Parkinson's Disease Detection from Voice. *Int. J. Med. Inf.* **2023**, *179*, 105237, doi:10.1016/j.ijmedinf.2023.105237.
47. Hoq, M.; Uddin, M.N.; Park, S.-B. Vocal Feature Extraction-Based Artificial Intelligent Model for Parkinson's Disease Detection. *Diagnostics* **2021**, *11*, 1076, doi:10.3390/diagnostics11061076.
48. Hossain, M.A.; Amenta, F. Machine Learning-Based Classification of Parkinson's Disease Patients Using Speech Biomarkers. *Journal of Parkinson's Disease* **2023**, *14*, 95–109, doi:10.3233/JPD-230002.
49. Ibarra, E.J.; Arias-Londoño, J.D.; Zañartu, M.; Godino-Llorente, J.I. Towards a Corpus (and Language)-Independent Screening of Parkinson's Disease from Voice and Speech through Domain Adaptation. *Bioengineering* **2023**, *10*, 1316, doi:10.3390/bioengineering10111316.

50. Iyer, A.; Kemp, A.; Rahmatallah, Y.; Pillai, L.; Glover, A.; Prior, F.; Larson-Prior, L.; Virmani, T. A Machine Learning Method to Process Voice Samples for Identification of Parkinson's Disease. *Sci. Rep.* **2023**, *13*, 20615, doi:10.1038/s41598-023-47568-w.
51. Karabayir, I.; Goldman, S.M.; Pappu, S.; Akbilgic, O. Gradient Boosting for Parkinson's Disease Diagnosis from Voice Recordings. *BMC Med. Inf. Decis. Making* **2020**, *20*, 228, doi:10.1186/s12911-020-01250-7.
52. Karaman, O.; Çakın, H.; Alhudhaif, A.; Polat, K. Robust Automated Parkinson Disease Detection Based on Voice Signals with Transfer Learning. *Expert Syst. Appl.* **2021**, *178*, 115013, doi:10.1016/j.eswa.2021.115013.
53. Karapinar Senturk, Z. Early Diagnosis of Parkinson's Disease Using Machine Learning Algorithms. *Med. Hypotheses* **2020**, *138*, 109603, doi:10.1016/j.mehy.2020.109603.
54. Kiran Reddy, M.; Alku, P. Automatic Detection of Parkinsonian Speech Using Wavelet Scattering Features. *JASA Express Lett.* **2025**, *5*, 055202, doi:10.1121/10.0036660.
55. Klempíř, O.; Krupička, R. Analyzing Wav2Vec 1.0 Embeddings for Cross-Database Parkinson's Disease Detection and Speech Features Extraction. *Sens.* **2024**, *24*, 5520, doi:10.3390/s24175520.
56. Klempir, O.; Skryjova, A.; Tichopad, A.; Krupicka, R. Ranking Pre-Trained Speech Embeddings in Parkinson's Disease Detection: Does Wav2Vec 2.0 Outperform Its 1.0 Version across Speech Modes and Languages? *Comput. Struct. Biotechnol. J.* **2025**, *27*, 2584–2601, doi:10.1016/j.csbj.2025.06.022.
57. Kumar, A.; Singh, J.P.; Paygude, P.; Daimary, R.; Prasad, S. Advanced Comparative Analysis of Machine Learning Algorithms for Early Parkinson's Disease Detection Using Vocal Biomarkers. *DIGITAL HEALTH* **2025**, *11*, 20552076251342878, doi:10.1177/20552076251342878.
58. Kumari, R.; Ramachandran, P. Novel Multistage Deep Convolution Neural Network-Based Parkinson's Disease Detection and Severity Grading of Running Speech Using LSF Spectrums for Detection and STFT Spectrums for Grading. *Results Eng.* **2025**, *27*, 106642, doi:10.1016/j.rineng.2025.106642.
59. Meral, M.; Ozbilgin, F.; Durmus, F. Fine-Tuned Machine Learning Classifiers for Diagnosing Parkinson's Disease Using Vocal Characteristics: A Comparative Analysis. *Diagnostics* **2025**, *15*, 645, doi:10.3390/diagnostics15050645.
60. Mohammadi, A.G.; Mehralian, P.; Naseri, A.; Sajedi, H. Parkinson's Disease Diagnosis: The Effect of Autoencoders on Extracting Features from Vocal Characteristics. *Array* **2021**, *11*, 100079, doi:10.1016/j.array.2021.100079.
61. Mohapatra, S.; Swain, B.K.; Mishra, M. Early Parkinson's Disease Identification via Hybrid Feature Selection from Multi-Feature Subsets and Optimized CatBoost with SMOTE. *Syst. Sci. Control Eng.* **2025**, *13*, doi:10.1080/21642583.2025.2498909.
62. Momeni, N.; Whitling, S.; Jakobsson, A. Interpretable Parkinson's Disease Detection Using Group-Wise Scaling. *IEEE Access* **2025**, *13*, 29147–29161, doi:10.1109/ACCESS.2025.3540600.
63. Motin, M.A.; Pah, N.D.; Raghav, S.; Kumar, D.K. Parkinson's Disease Detection Using Smartphone Recorded Phonemes in Real World Conditions. *IEEE Access* **2022**, *10*, 97600–97609, doi:10.1109/ACCESS.2022.3203973.
64. Naeem, I.; Ditta, A.; Mazhar, T.; Anwar, M.; Saeed, M.M.; Hamam, H. Voice Biomarkers as Prognostic Indicators for Parkinson's Disease Using Machine Learning Techniques. *Sci. Rep.* **2025**, *15*, 12129, doi:10.1038/s41598-025-96950-3.
65. Narendra, N.P.; Schuller, B.; Alku, P. The Detection of Parkinson's Disease From Speech Using Voice Source Information. *IEEE ACM Trans. Audio, Speech, Language Process.* **2021**, *29*, 1925–1936, doi:10.1109/TASLP.2021.3078364.
66. Nijhawan, R.; Kumar, M.; Arya, S.; Mendirtta, N.; Kumar, S.; Towfek, S.K.; Khafaga, D.S.; Alkahtani, H.K.; Abdelhamid, A.A. A Novel Artificial-Intelligence-Based Approach for Classification of Parkinson's Disease Using Complex and Large Vocal Features. *Biomimetics* **2023**, *8*, 351, doi:10.3390/biomimetics8040351.
67. Noaman Kadhim, M.; Al-Shammary, D.; Sufi, F. A Novel Voice Classification Based on Gower Distance for Parkinson Disease Detection. *Int. J. Med. Inf.* **2024**, *191*, 105583, doi:10.1016/j.ijmedinf.2024.105583.

68. Oliveira, G.C.; Pah, N.D.; Ngo, Q.C.; Yoshida, A.; Gomes, N.B.; Papa, J.P.; Kumar, D. A Pilot Study for Speech Assessment to Detect the Severity of Parkinson's Disease: An Ensemble Approach. *Comput. Biol. Med.* **2025**, *185*, 109565, doi:10.1016/j.compbiomed.2024.109565.
69. Pah, N.D.; Indrawati, V.; Kumar, D.K. Voice-Based SVM Model Reliability for Identifying Parkinson's Disease. *IEEE Access* **2023**, *11*, 144296–144305, doi:10.1109/ACCESS.2023.3344464.
70. Pandey, P.V.K.; Sahu, S.S. Parkinson's Disease Detection Using Hybrid Siamese Neural Network and Support Vector Machine in Multilingual Voice Signal. *J. Voice* **2025**, S0892-1997(25)00261-9, doi:10.1016/j.jvoice.2025.06.036.
71. Qasim, H.M.; Ata, O.; Ansari, M.A.; Alomary, M.N.; Alghamdi, S.; Almehmadi, M. Hybrid Feature Selection Framework for the Parkinson Imbalanced Dataset Prediction Problem. *Med. Lith.* **2021**, *57*, 1217, doi:10.3390/medicina57111217.
72. Rahmatallah, Y.; Kemp, A.S.; Iyer, A.; Pillai, L.; Larson-Prior, L.J.; Virmani, T.; Prior, F. Pre-Trained Convolutional Neural Networks Identify Parkinson's Disease from Spectrogram Images of Voice Samples. *Sci. Rep.* **2025**, *15*, 7337, doi:10.1038/s41598-025-92105-6.
73. Rehman, A.; Saba, T.; Mujahid, M.; Alamri, F.S.; ElHakim, N. Parkinson's Disease Detection Using Hybrid LSTM-GRU Deep Learning Model. *Electronics* **2023**, *12*, 2856, doi:10.3390/electronics12132856.
74. Rey-Paredes, M.; Pérez, C.J.; Mateos-Caballero, A. Time Series Classification of Raw Voice Waveforms for Parkinson's Disease Detection Using Generative Adversarial Network-Driven Data Augmentation. *IEEE Open J. Comput. Soc.* **2025**, *6*, 72–84, doi:10.1109/OJCS.2024.3504864.
75. Sedigh Malekroodi, H.; Madusanka, N.; Lee, B.; Yi, M. Speech-Based Parkinson's Detection Using Pre-Trained Self-Supervised Automatic Speech Recognition (ASR) Models and Supervised Contrastive Learning. *Bioengineering* **2025**, *12*, 728, doi:10.3390/bioengineering12070728.
76. Shen, M.; Mortezaagha, P.; Rahgozar, A. Explainable Artificial Intelligence to Diagnose Early Parkinson's Disease via Voice Analysis. *Sci. Rep.* **2025**, *15*, 11687, doi:10.1038/s41598-025-96575-6.
77. Singh, N.; Tripathi, P. An Ensemble Technique to Predict Parkinson's Disease Using Machine Learning Algorithms. *Speech Commun.* **2024**, *159*, 103067, doi:10.1016/j.specom.2024.103067.
78. Srinivasan, S.; Ramadass, P.; Mathivanan, S.K.; Panneer Selvam, K.; Shivahare, B.D.; Shah, M.A. Detection of Parkinson Disease Using Multiclass Machine Learning Approach. *Sci. Rep.* **2024**, *14*, 13813, doi:10.1038/s41598-024-64004-9.
79. Tougui, I.; Zakroum, M.; Karrakchou, O.; Ghogho, M. Transformer-Based Transfer Learning on Self-Reported Voice Recordings for Parkinson's Disease Diagnosis. *Sci. Rep.* **2024**, *14*, 30131, doi:10.1038/s41598-024-81824-x.
80. Valarmathi, P.; Suganya, Y.; Saranya, K.R.; Shanmuga Priya, S. Enhancing Parkinson Disease Detection through Feature Based Deep Learning with Autoencoders and Neural Networks. *Sci. Rep.* **2025**, *15*, 8624, doi:10.1038/s41598-025-88293-w.
81. Vásquez-Correa, J.C.; Fritsch, J.; Orozco-Arroyave, J.R.; Nöth, E.; Magimai-Doss, M. On Modeling Glottal Source Information for Phonation Assessment in Parkinson’s Disease. In Proceedings of the Interspeech 2021; ISCA, August 30 2021; pp. 26–30.
82. Veetil, I.K.; V., S.; Orozco-Arroyave, J.R.; Gopalakrishnan, E.A. Robust Language Independent Voice Data Driven Parkinson's Disease Detection. *Eng. Appl. Artif. Intell.* **2024**, *129*, 107494, doi:10.1016/j.engappai.2023.107494.
83. Velu, K.; Jaisankar, N. Design of an Early Prediction Model for Parkinson's Disease Using Machine Learning. *IEEE Access* **2025**, *13*, 17457–17472, doi:10.1109/ACCESS.2025.3533703.
84. Xu, Z.-J.; Wang, R.-F.; Wang, J.; Yu, D.-H. Parkinson's Disease Detection Based on Spectrogram-Deep Convolutional Generative Adversarial Network Sample Augmentation. *IEEE Access* **2020**, *8*, 206888–206900, doi:10.1109/ACCESS.2020.3037775.
85. Xu, H.; Xie, W.; Pang, M.; Li, Y.; Jin, L.; Huang, F.; Shao, X. Non-Invasive Detection of Parkinson's Disease Based on Speech Analysis and Interpretable Machine Learning. *Front. Aging Neurosci.* **2025**, *17*, 1586273, doi:10.3389/fnagi.2025.1586273.

86. Yang, Z.; Zhou, H.; Srivastav, S.; Shaffer, J.G.; Abraham, K.E.; Naandam, S.M.; Kakraba, S. Optimizing Parkinson's Disease Prediction: A Comparative Analysis of Data Aggregation Methods Using Multiple Voice Recordings via an Automated Artificial Intelligence Pipeline. *Data* **2025**, *10*, 4, doi:10.3390/data10010004.
87. Zahid, L.; Maqsood, M.; Durrani, M.Y.; Bakhtyar, M.; Baber, J.; Jamal, H.; Mehmood, I.; Song, O.-Y. A Spectrogram-Based Deep Feature Assisted Computer-Aided Diagnostic System for Parkinson's Disease. *IEEE Access* **2020**, *8*, 35482–35495, doi:10.1109/ACCESS.2020.2974008.
